# A Dibenzotetrathiafulvalene-Bridged Bis(alkenylruthenium) Complex and Its One- and Two-Electron-Oxidized Forms

**DOI:** 10.1021/acs.inorgchem.3c03184

**Published:** 2023-11-03

**Authors:** Franciska S. Gogesch, Lukas S. Laininger, Nick Sokov, Stefan M. Schupp, Laura Senft, Hipassia M. Moura, Michael Linseis, Lukas Schmidt-Mende, Ivana Ivanović-Burmazović, Miriam M. Unterlass, Rainer F. Winter

**Affiliations:** † Fachbereich Chemie Universität Konstanz Universitätsstraße 10, 78457 Kostanz, Germany; ‡ Universität Konstanz Universitätsstraße 10, 78457 Konstanz, Germany; § Department Chemie Ludwig-Maximilians-Universität München Butenandstraße 5−13, Haus D, 81377 München, Germany

## Abstract

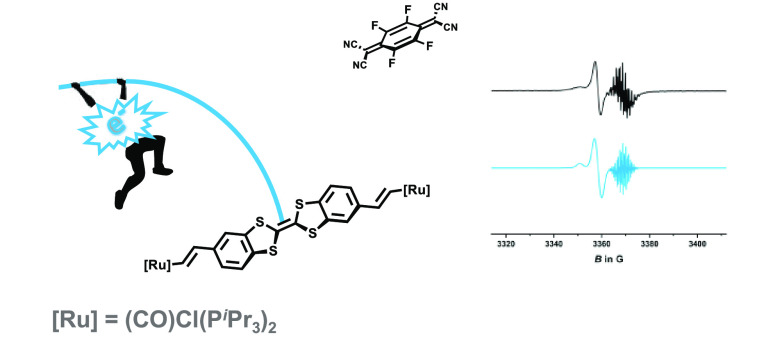

We report on the synthesis of the new bis(alkenylruthenium) complex **DBTTF-(ViRu)**_**2**_ with a longitudinally extended, π-conjugated dibenzotetrathiafulvalene (DBTTF) bridge, characterized by multinuclear NMR, IR, and UV/vis spectroscopy, mass spectrometry, and single-crystal X-ray diffraction. Cyclic and square-wave voltammetry revealed that **DBTTF-(ViRu)**_**2**_ undergoes four consecutive oxidations. IR, UV/vis/near-IR, and electron paramagnetic resonance spectroscopy indicate that the first oxidation involves the redox-noninnocent DBTTF bridge, while the second oxidation is biased toward one of the peripheral styrylruthenium entities, thereby generating an electronically coupled mixed-valent state ({Ru}-CH=CH)^•+^-DBTTF^•+^-(CH=CH-{Ru}) [{Ru} = Ru(CO)Cl(P^*i*^Pr_3_)_2_]. The latter is apparently in resonance with the ({Ru}-CH=CH)^•+^-DBTTF-(CH=CH-{Ru})^•+^ and ({Ru}-CH=CH)-DBTTF^2+^-(CH=CH-{Ru}) forms, which are calculated to lie within 19 kJ/mol. Higher oxidized forms proved too unstable for further characterization. The reaction of **DBTTF-(ViRu)**_**2**_ with the strong organic acceptors 2,3-dichloro-5,6-dicyano-1,4-benzoquinone, tetracyano-*p*-benzoquinodimethane (TCNQ), and F_4_TCNQ resulted in formation of the **DBTTF-(ViRu)**_**2**_^**•+**^ radical cation, as shown by various spectroscopic techniques. Solid samples of these compounds were found to be highly amorphous and electrically insulating.

## Abstract 2

The title dibenzotetrathiafulvalene (DBTTF)-bridged diruthenium complex undergoes four consecutive one-electron oxidations. Spectroscopic investigations and quantum-chemical studies identify the DBTTF ligand as the primary oxidation site. The dication possesses an intricate electronic structure, where the triplet state with the DBTTF ligand and one {Ru} moiety is oxidized, which is only slightly below the open-shell singlet state with two oxidized {Ru} entities and the singlet state with a dioxidized DBTTF ligand.

## Introduction

Tetrathiafulvalene (TTF) is the original donor used in the first studies on electrically conductive charge-transfer (CT) compounds.^1−5^ TTF and its many derivatives combine the advantages of being electron-rich and showing two reversible stepwise oxidations to first the radical cation and then the closed-shell dication, both of which are chemically stable. Moreover, their extended, planar π system offers a structural template well-poised for π stacking, while the sulfur atoms may engage in intermolecular S···X and S···S interactions. All of these assets have made TTFs favorite donor constituents of CT compounds when combined with a strong electron acceptor.^1,6−17^

**Figure 1:**
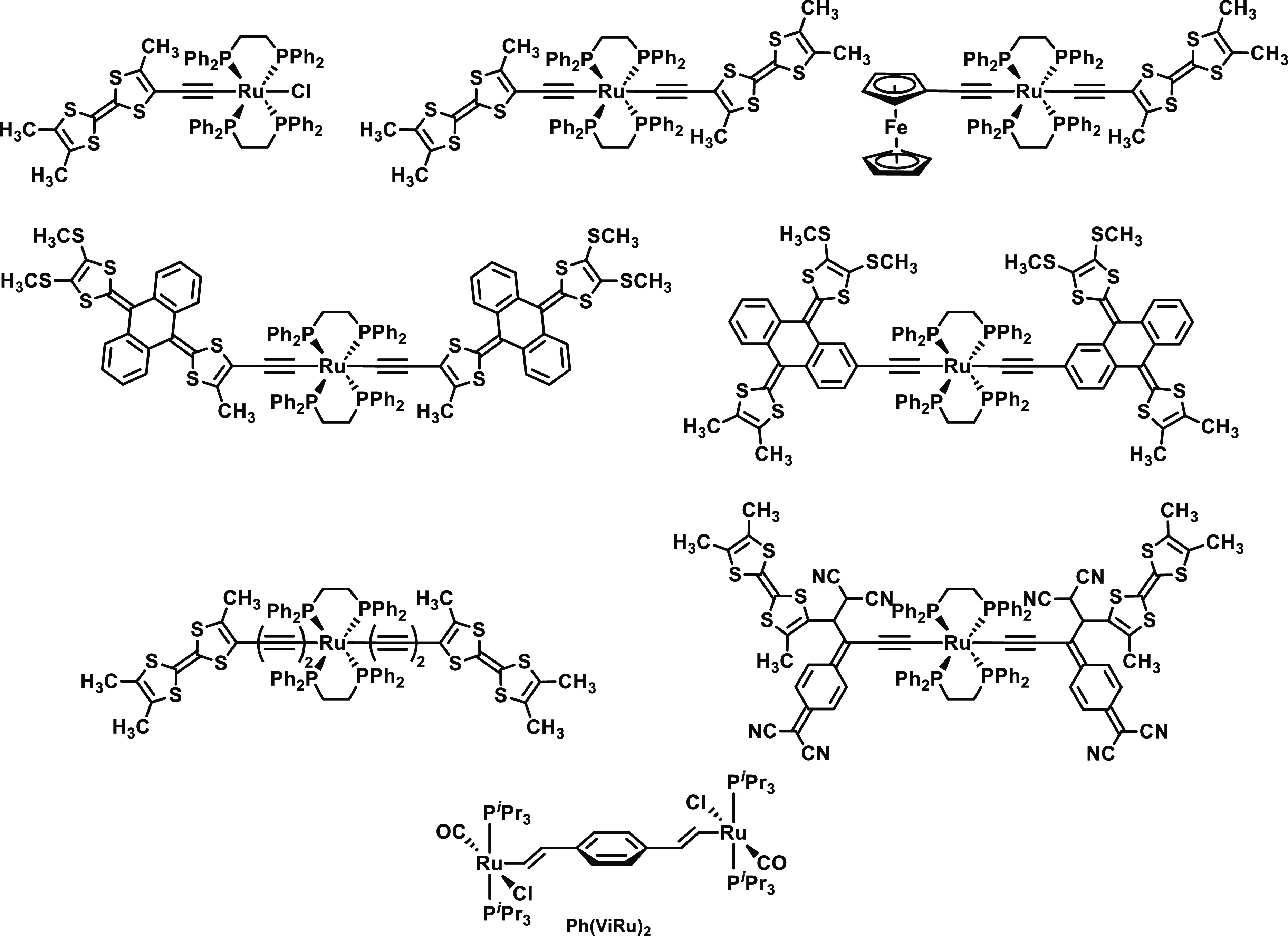
Previously reported (ex)TTF ruthenium complexes and the divinylphenylene-bridged diruthenium complex **Ph(ViRu)**_**2**_.

Despite the broad availability of organic TTFs, comparatively few representatives of organometallic TTFs were designed. Best represented are ferrocenyl (Fc) derivatives with direct Fc-TTF connectivities or Fc-Arylene-TTF dyads.^18−29^ In addition, the Lorcy group has published a series of elegant studies on ruthenium, platinum, and mercury complexes with one or two ethynyl-TTF or extended TTF (exTTF) ligands. Structures of the ruthenium complexes of relevance to the present work are summarized in Figure 1.^30−36^ Mono- and bis-ethynyl (ex)TTF ruthenium complexes possess a rich electrochemistry with one chemically reversible redox wave per Ru(dppe)_2_ and TTF constituent and significantly lower first oxidation potentials than any of their individual redox-active entities. Moreover, the unpaired spin densities and charge(s) of their one-electron-oxidized or one- and two-electron-oxidized forms are delocalized over all redox sites. This makes these compounds attractive electron donors. However, to the best of our knowledge, no dinuclear metal complexes with diethynyl-TTF or benzannulated TTF ligands bridging two metal-coligand entities and CT compounds derived from such donors have been reported to date.

In this study, we report first forays into this subject, employing dibenzotetrathiafulvalene (DBTTF) as the linker. Instead of the commonly employed metal-acetylide connector, we use the metal-alkenyl functionality, which often provides even stronger electronic interactions. The insertion of a terminal alkyne into the ruthenium hydride bond of complexes [HRu(CO)Cl(PR_3_)_*n*_] (R = Ph, *n* = 2 or 3; R = PCy_3_, P^*i*^Pr_3_, or P^*t*^Bu_2_Me, *n* = 2) provides a convenient, high-yielding route to such complexes.^37−50^ This was done with the aim of exploring the impact of an increased lateral extension and benzannulation of the linker on the ligand/metal character of the individual oxidations and the extent of electronic interaction between the appended {Ru(CO)Cl(P^*i*^Pr_3_)_2_} entities (henceforth denoted as {Ru}). We further speculated that a larger lateral extension of the TTF template would provide a central cleft of sufficient size to allow for π-stacking interactions with a suitable organic acceptor. Previous work on bis(alkenyl)arylene-bridged diruthenium complexes [{(PR_3_)_2_(L)(X)(CO)Ru}_2_(μ-CH=CH-Arylene-CH=CH)] [L = PR_3_, pyridine derivative, or vacant coordination site; X = Cl^–^ or (L)(X) = a bidentate monoanionic carboxylate, β-ketoenolate, or benzothiadiazolate four-electron donor ligand] has shown that these complexes exhibit favorable electron-transfer properties, such as two or more stepwise oxidations, low oxidation potentials, and strong polyelectrochromism with intense absorptions of their oxidized forms in the visible (Vis) and near-infrared (NIR).^42,48,49,51−72^ The *p*-divinylphenylene-bridged diruthenium complex [{(PR_3_)_2_(L)(X)(CO)Ru}_2_(μ-CH=CH-*p*-C_6_H_4_–CH=CH)] [**Ph(ViRu)**_**2**_] was found to react with several organic acceptors, but the resulting CT compounds were unstable and decomposed rapidly, while CT salts of complexes with laterally extended, π-conjugated polycyclic aromatic hydrocarbyl ligands as the linkers offered superior benchtop lifetimes.^64^ Our present work expands on these earlier studies, employing the (*E*)-5,6′- or (*Z*)-5,5′-DBTTF-bridged bis(alkenylruthenium) complex **DBTTF-(ViRu)**_**2**_ as the donor.

## Results and Discussion

### Synthesis and Characterization

**Scheme 1:**
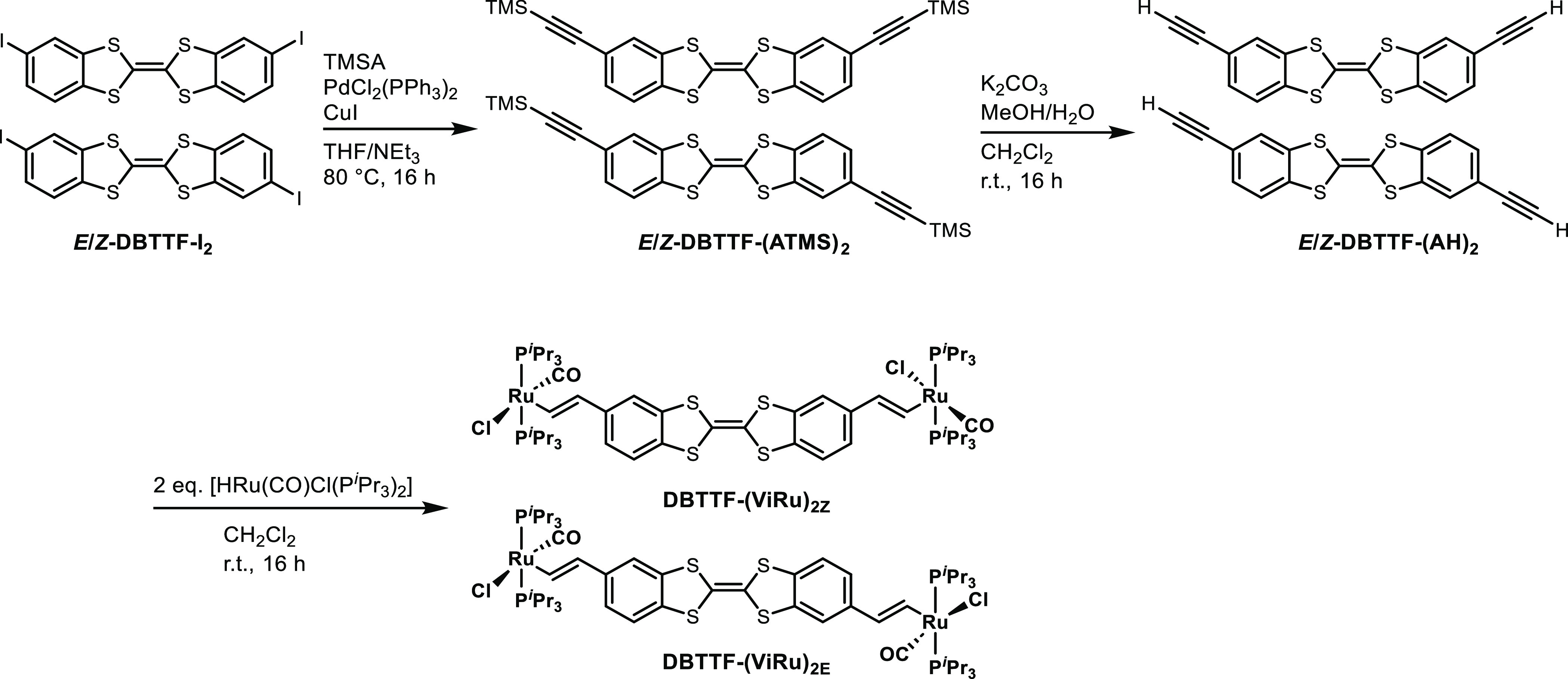
Synthesis of **DBTTF-(ViRu)**_**2E/Z**_ and Its Organic Precursors

The DBTTF-bridged bis(alkenylruthenium) complex **DBTTF-(ViRu)**_**2**_ was obtained in a six-step synthesis according to Scheme 1. The first two steps involve the conversion of commercially available 2-amino-4-iodobenzoic acid to literature-known **DBTTF-I**_**2**_.^73^ A further reaction with Me_3_Si-C≡CH (TMSA) under Sonogashira coupling conditions yielded the protected dialkyne **DBTTF-(ATMS)**_**2**_ (“A” denotes the alkynyl functionality) in 81% yield. The diterminal dialkyne **DBTTF-(AH)**_**2**_ was obtained by removal of the Me_3_Si protecting groups under basic conditions and then reacted with 2 equiv of the hydride complex [HRu(CO)Cl(P^*i*^Pr_3_)_2_]([H-{Ru}]) to provide the bis(alkenyl)-DBTTF-bridged diruthenium title complex as an (*E*)-5,6′/(*Z*)-5,5′ isomeric mixture in an isolated yield of 70%. Synthetic procedures are provided in the Experimental Methods and Materials section. **DBTTF-(ViRu)**_**2**_ is air- and moisture-stable in the solid state as well as in solution.

Multinuclear NMR spectroscopy and mass spectrometry of the precursors and of **DBTTF-(ViRu)**_**2**_ confirmed their purities (Figures S1–S11). Close inspection of the ^1^H NMR spectra revealed that **DBTTF-(ViRu)**_**2**_ exists as two isomers that differ with respect to the mutual arrangement of the vinyl-{Ru} moieties. The latter are in either a transoid [**DBTTF-(ViRu)**_**2E**_, the 5,6′-DBTTF isomer] or a cisoid [**DBTTF-(ViRu)**_**2Z**_, the 5,5′-DBTTF isomer] orientation. The two isomers are only distinguished by slight shift differences of the protons on the benzene rings annulated with the TTF core but by neither their ^13^C{^1^H} nor their ^31^P{^1^H} NMR resonance shifts. The isomeric ratio of the as-isolated product is 1.5:1 (*E*/*Z*) but changes upon crystallization because the *E* isomer crystallizes preferentially (Figures S4, S5, and S9). Quantum-chemical calculations indicate that the energy barrier for rotation around the central C=C bond in **DBTTF-(ViRu)**_**2**_ of 180 kJ/mol is prohibitively high. Moreover, the entire reaction pathway from **DBTTF-I**_**2**_ to the final complex involves no step that could possibly trigger *E*/*Z* isomerization at this bond. We therefore assumed that the isomeric mixture already exists at the stage of **DBTTF-I**_**2**_. This was confirmed by comparing the ^1^H NMR spectra of the precursor **DBTTF-(ATMS)**_**2**_ recorded on 400 and 800 MHz NMR instruments (Figure S8). The formation of *E*/*Z* isomeric mixtures of disubstituted DBTTFs was suspected previously.^73^ For simplicity reasons, all graphical representations except for Scheme 1 only show the *E* isomer **DBTTF-(ViRu)**_**2E**_.

In the ^1^H NMR spectrum, the α- and β-vinyl protons of the title complex give rise to two doublet resonances located at δ = 8.99 ppm (Ru-C*H*) and 6.17 ppm (Ru-CH=C*H*) with additional ^4^*J*_PH_ couplings for the latter. The ^3^*J*_HH_ coupling constant of 13.4 Hz agrees with the expected *E* configuration. The ^13^C{^1^H} NMR spectrum of **DBTTF-(ViRu)**_**2**_ displays triplet or singlet resonances for the carbonyl and vinyl carbon atoms at δ = 203.3 ppm (*C*O, ^2^*J*_CP_ = 12.9 Hz), 152.1 ppm (t, ^2^*J*_CP_ = 10.5 Hz, Ru-*C*H), and 133.3 ppm (Ru-CH=*C*H), in addition to those of DBTTF and P^*i*^Pr_3_ ligands. The phosphorus atoms of the P^*i*^Pr_3_ ligands give rise to one sharp singlet resonance in the ^31^P{^1^H} spectrum at δ = 38.50 ppm.

**Figure 2:**
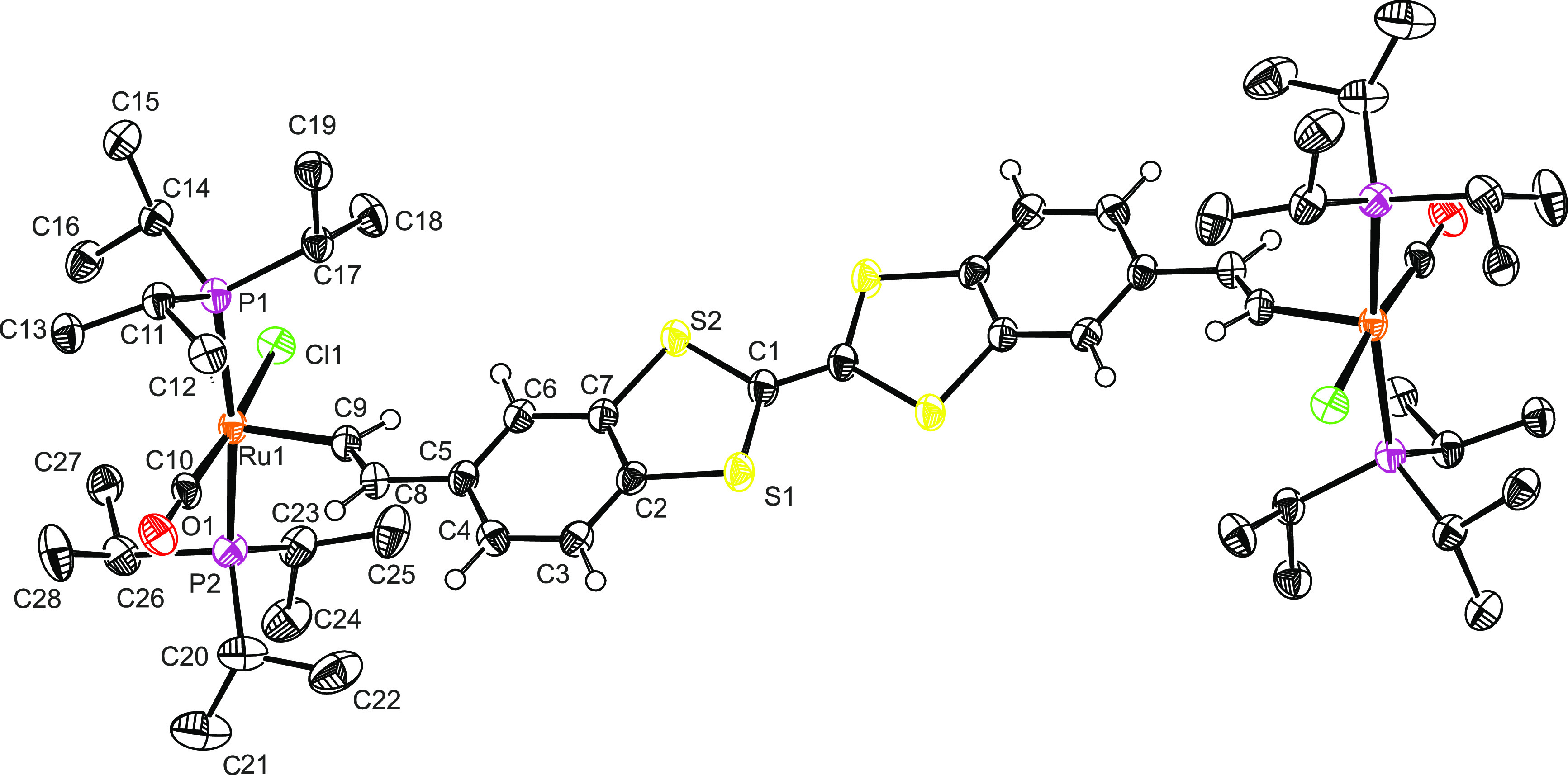
ORTEP of the molecular structure of **DBTTF-(ViRu)**_**2E**_ in the crystalline state with atomic numbering. Hydrogen atoms of the phosphane ligands have been omitted for clarity. Ellipsoids are displayed at the 50% probability level.

Unequivocal confirmation of the identity of **DBTTF-(ViRu)**_**2**_ was obtained by single-crystal X-ray diffraction analysis. Single crystals of the *E* isomer were obtained from a dichloromethane/*n*-pentane solvent mixture. Its molecular structure in the crystalline state is shown in Figure 2. Multiple attempts to also obtain single crystals of the *Z* isomer failed because the latter formed conglomerates of small microcrystals that unfortunately did not diffract. NMR spectroscopic investigations showed the crystallized material to be enriched with the *E* isomer, while the mother liquors showed a concomitant increase of the proportion of the *Z* isomer. This allowed for the assignment of different proton resonances to the individual isomers.

**DBTTF-(ViRu)**_**2E**_ crystallizes in the monoclinic space group *P*2_1_/*n*. Details for the data collection, structure solution, and refinement, as well as listings of interatomic distances, bond angles, and torsion angles are provided in Tables S1–S4. The five-coordinated ruthenium atoms adopt a slightly distorted square-pyramidal coordination geometry, with the alkenyl ligand occupying the apical site and the carbonyl, chloro, and phosphane ligands in mutual trans positions at the basal sites. This preserves the favorable trans arrangement of the Cl π-donor and CO π-acceptor ligands and places the ligand with the strongest σ trans influence opposite to the vacant coordination site. Deviations from a perfect square pyramid consist of a slight compression of the bond angles between the chloro and phosphane ligands to 88.28(3)° (Cl1–Ru1–P1) and 88.49(3)° (Cl1–Ru1–P2) and of one C(CO)–Ru–P bond angle C10–Ru–P2 to 87.75(9)° as well as the concomitant opening of the bond angle C10–Ru1–P1 to 94.27(9)°. The bond angles between the alkenyl carbon atom C9 at the apical site and the chloro ligand [C9–Ru1–Cl1 = 98.23(9)°] and one of the phosphane ligands [C9–Ru1–P2 = 98.57(8)°] are also obtuse, while those to the other two basal ligands approach the ideal angle of 90° [C9–Ru1–C10 = 89.58(13)°; C9–Ru1–P1 = 90.72(8)°]. The bond between the ruthenium atom and the vinyl carbon atom C9 of 1.989(3) Å is appreciably longer than the Ru–C10 bond of 1.814(3) Å, as the combined result of a lower Ru–C9 bond order and the larger atomic radius of a sp^2^- compared to a sp-hybridized carbon atom. Bond lengths C9–C8 of 1.337(4) Å and C1–C1a of 1.349(6) Å agree with the C=C bonds.^74^ Within the TTF core, the C2–S1 and C7–S2 bonds of 1.760(3) and 1.753(3) Å are nearly identical, despite the asymmetric substitution at the annulated benzene rings. The benzodithiene units are not entirely planar but are kinked along the S···S vector with an interplanar angle of 11.57° between the phenyl and the S1–C1–S2 planes. The phenyl planes at the two sides of the DBTTF core are exactly parallel to each other but displaced with a vertical offset of 0.466 Å due to a torsion C7–S2–C1–C1a of 168.6(4)° (Figure S12). The Ru-CH=CH unit is rotated out of the phenyl plane by an even larger angle of 23.78°.

In the crystal, coparallel-aligned complex molecules **DBTTF-(ViRu)**_**2E**_ arrange into rows. Molecules that belong to neighboring rows are tilted along their Ru···Ru vectors by 76.77° (Figure S13). Individual molecules associate via S···Cl interactions of 3.348 Å, which is by 0.202 Å shorter than the sum of the van der Waals radii, and by several CH···π interactions of 2.753–2.871 Å between methyl protons at the P^*i*^Pr_3_ ligands and carbon atoms of the phenyl rings of the DBTTF backbone. These interactions are shown in Figure S14.

### Electrochemistry

**Figure 3:**
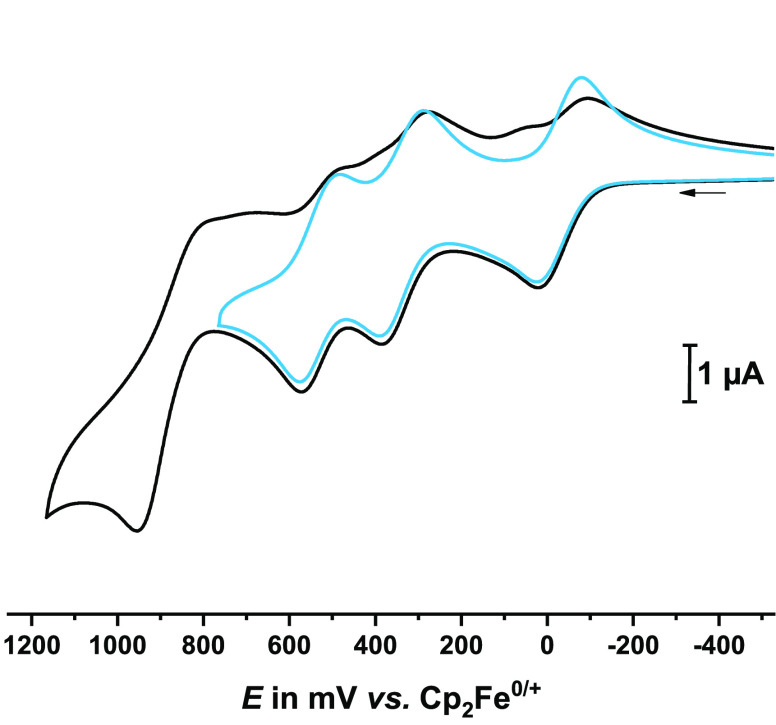
Cyclic voltammogram of all oxidations (black line) and of the first three oxidations (blue line) of **DBTTF-(ViRu)**_**2**_ at *v* = 100 mV/s.

The electrochemical properties of **DBTTF-(ViRu)**_**2**_ were investigated by cyclic and square-wave voltammetry. Measurements were conducted in dichloromethane in the presence of 0.1 M tetrabutylammonium hexafluorophosphate (^*n*^Bu_4_N^+^PF_6_^–^) as the supporting electrolyte. The results are shown in Figures 3 and S16. For subsequent forays into its propensity to react as an electron donor, we also recorded voltammograms of common acceptors under identical conditions. The results are shown in Figures S17–S23, while pertinent data are collected in Table 1.

**Table 1:** Half-Wave Potentials of **DBTTF-(ViRu)**_**2**_, Its TMS-Protected Dialkyne Precursor **DBTTF-(ATMS)**_**2**_, Divinylphenylene-Bridged Diruthenium Complex **Ph(ViRu)**_**2**_, and the Organic Acceptors Used in This Study Measured in 0.1 M CH_2_Cl_2_/^*n*^Bu_4_N^+^PF_6_^–^ at rt. Potentials are given in Millivolts and Calibrated against the Cp_2_Fe^0/+^ Standard

Donors D				
	*E*_1/2_^0/+^	*E*_1/2_^+/2+^	*E*_1/2_^2+/3+^	*E*_1/2_^3+/4+^
**DBTTF-(ViRu)**_**2**_	–22	338	530	890
**DBTTF-(ATMS)**_**2**_	244	700		
**Ph(ViRu)**_**2**_	–75	175		

Acceptors A		
	*E*1/2^0/–^	*E*1/2^–/2–^
F_4_TCNQ	153	–484
DDQ	138	–746
TCNQ	–270	–850
F_4_BQ	–448	–1336
Cl_4_BQ	–452	–1236
Br_4_BQ	–464	–1224

The diruthenium complex **DBTTF-(ViRu)**_**2**_ displays four consecutive, well-separated one-electron oxidations at half-wave potentials *E*_1/2_ of −22, 338, and 530 mV and an anodic peak potential of 955 mV, respectively. The first three redox processes are chemically and electrochemically reversible (Figure 3). Further oxidation to the tetracation, however, triggers chemical follow-up processes, as revealed by the loss of the cathodic peak currents associated with the individual oxidations and the appearance of additional cathodic peaks on the reverse scan. At lower temperature *T* and/or higher sweep rates *v*, the associated counter peak of the 3+/4+ wave can still be detected, which allows us to determine the half-wave potential of this process as 890 mV (Table 1). No suspicious splitting or broadening of the individual redox waves can be discerned, implying that the *E* and *Z* isomers oxidize at the same potential (or very nearly so). Quantum-chemical calculations place the respective frontier orbitals of the two isomers at the same energy.

A comparison with the TMS-protected dialkyne precursor **DBTTF-(ATMS)**_**2**_ reveals that the half-wave potentials of the first two oxidations of **DBTTF-(ViRu)**_**2**_ are lowered by 266 or 362 mV. Cathodic shifts of similar magnitude were previously observed in ruthenium complexes with one or two ethynyl-TTF ligands.^30,31,36^ On the other hand, they are shifted to slightly or significantly more positive values than in the 1,4-divinylphenylene-bridged diruthenium complex {Ru}-CH=CH-*p*-C_6_H_4_–CH=CH-{Ru} [**Ph(ViRu)**_**2**_].^72^ The presence of three interlinked redox-active constituents, two of which are chemically different, in direct π conjugation renders an a priori assignment of the individual redox waves to any specific redox site impossible. This aspect will be elaborated on in the following by applying a combined experimental and quantum-chemical approach.

### Electronic Structures of the Oxidized Forms of **DBTTF-(ViRu)_2_** as Probed by Spectroelectrochemistry, Electron Paramagnetic Resonance (EPR) Spectroscopy, and Quantum Chemistry

**Figure 4:**
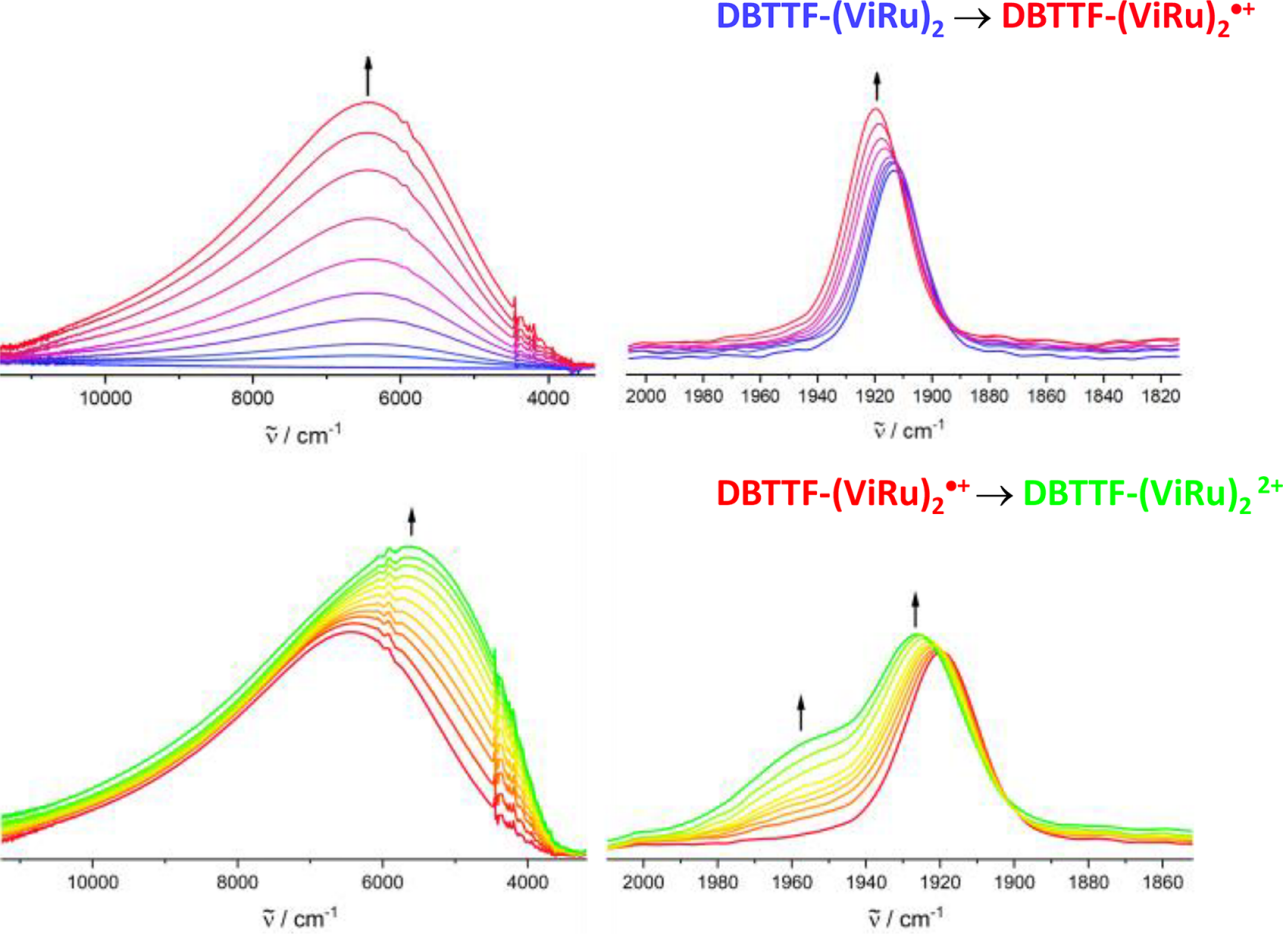
Changes of the IR/NIR spectra in the region of CT (left) and the Ru(CO) bands (right) during electrochemical oxidation of **DBTTF-(ViRu)**_**2**_ to its monocation (top) and during further oxidation to its dication (bottom) in 0.2 M 1,2-C_2_H_4_Cl_2_/^*n*^Bu_4_N^+^PF_6_^–^ at rt.

In order to experimentally probe the identity of the redox sites for the individual oxidations and investigate how the positive charge(s) and spin densities distribute over the π-conjugated {Ru}-CH=CH-DBTTF-CH=CH-{Ru} backbone, we generated the accessible oxidized forms of **DBTTF-(ViRu)**_**2**_ by using electrochemical as well as chemical methods and investigated them spectroscopically. The electrochemical approach employs *in situ* spectroelectrochemistry, i.e., oxidation inside an optically and IR-transparent thin-layer electrolysis cell, with simultaneous monitoring of the ensuing changes in IR/NIR or UV/vis/NIR spectra. The carbonyl ligand at each terminally appended ruthenium atom serves as a sensitive IR tag with an inherently high oscillator strength. Due to the synergistic nature of the M–CO bond, charge density loss from a {Ru} entity causes a blue shift of the CO stretching vibration, whose magnitude scales with metal contributions to the respective oxidation. *In situ* studies were complemented by chemical oxidation of **DBTTF-(ViRu)**_**2**_ with a suitable oxidizing agent.^75^ All spectroelectrochemical experiments were conducted in 1,2-dichloroethane in the presence of 0.2 M ^*n*^Bu_4_N^+^PF_6_^–^ as the supporting electrolyte. The latter electrolyte has properties very similar to those of the dichloromethane-based one used in the electrochemical studies but has a higher boiling point. This counteracts solvent evaporation or the formation of gas bubbles at the working electrode as a response to the locally generated heat. The results of the IR spectroelectrochemical measurements on **DBTTF-(ViRu)**_**2**_ are shown in Figure 4; relevant data are summarized in Table 2.

**Table 2:** Relevant Spectroscopic IR/NIR and UV/Vis/NIR Data of Precursors **DBTTF-I**_**2**_ and **DBTTF-(ATMS)**_**2**_ in the Neutral and One-Electron-Oxidized States and of Complex **DBTTF-(ViRu)**_**2**_ in Its Neutral, Monocationic, and Dicationic States, Measured in 1,2-C_2_H_4_Cl_2_/0.2 M NBu_4_^+^PF_6_^–^ at rt

	ν̃_CO_, cm^–1^	λ, nm (ε_max_, ×10^3^ M^–1^ cm^–1^)
**DBTTF-I**_**2**_		232 (48.6), 350 (9.2)
**DBTTF-(ATMS)**_**2**_		232 (42.6), 278 (59.8), 314 (25.6), 365 (9.1)
**DBTTF-(ATMS)**_**2**_^**•+**^		289 (15.3), 428 (15.2), 452 (15.9), 716 (13.6)
**DBTTF-(ViRu)**_**2**_	1913	226 (57.4), 331 (54.0), 367 (35.9), 505 (2.8)
**DBTTF-(ViRu)**_**2**_^**•+**^	1919	277 (18.9), 366 (34.3), 437 (17.0), 460 (16.4), 604 (5.1), 1530 (12.7)
**DBTTF-(ViRu)**_**2**_^**2+**^ a	1925, 1952	279 (26.7), 380 (23.7), 415 (24.6), 540 (8.7), 1710 (18.9)

a Extinction coefficients are lower estimates due to gradual decomposition, as observed after chemical oxidation.

Neutral **DBTTF-(ViRu)**_**2**_ exhibits a single Ru(CO) band at 1913 cm^–1^. During the first oxidation to **DBTTF-(ViRu)**_**2**_^**•+**^, the CO band position changes to 1919 cm^–1^ with a concomitant increase in absorptivity. The presence of only one Ru(CO) band in **DBTTF-(ViRu)**_**2**_^**•+**^ indicates that the charge density spreads symmetrically over the π-conjugated backbone and that both {Ru} entities remain electronically equivalent. However, the shift of merely 6 cm^–1^ is considerably smaller than that of 22 cm^–1^ for **Ph(ViRu)**_**2**_^**0→•+**^,^72^ those of 25–28 cm^–1^ in 2,5-divinylpyrrole-, -furan-, or -thiophene-bridged diruthenium complexes,^62^ or that of 43 cm^–1^ in an anthracene-1,8-diyl-bridged analogue,^68^ which all show complete charge delocalization. This indicates that the first oxidation is strongly biased toward the bridging DBTTF ligand. Bridge-based oxidations in bis(alkenylruthenium) complexes were already observed for other π-extended linkers such as a squaraine^76^ and, somwewhat surprisingly, 2,2′-bipyridine-4,4′-diyl.^67^

The bridge-based character of one-electron-oxidized **DBTTF-(ViRu)**_**2**_^**•+**^ was further corroborated by EPR spectroscopy. Samples containing the radical cation were generated by the addition of substoichiometric amounts of ferrocenium hexafluorophosphate to the corresponding neutral complex. As shown in Figure S24, **DBTTF-(ViRu)**_**2**_^**•+**^ produces an isotropic EPR signal with poorly resolved hyperfine splitting (hfs) to other nuclei with a *g* value of 2.000. Digital simulations with hfs constants *A*(^32^S) of 4.3 G, *A*(^31^P) of 1.1 G, and *A*(^99/101^Ru) of 0.9 G reproduced the experimental spectrum well. The EPR parameters match nearly perfectly with those of the pristine **DBTTF**^**•+**^ radical cation [*g* = 2.0068, *A*(^32^S) = 4.3 G].^77^ The EPR signal intensity decreases drastically upon cooling (Figure S24). Such behavior points to the reversible formation of spin-paired, EPR-silent radical cation dimers at low temperature, as was observed for other **DBTTF**^**•+**^ salts.^78−80^

Upon further oxidation to **DBTTF-(ViRu)**_**2**_^**2+**^, the single CO band at 1919 cm^–1^ evolves into a pair of bands consisting of more and less intense CO stretching vibrations at 1925 and 1952 cm^–1^, respectively (Figure 4). The increased average blue shift of ca. 20 cm^–1^ signals a larger involvement of the {Ru} entities in this second redox process. The pattern of two distinct Ru(CO) bands with an energy difference of 27 cm^–1^ characterizes **DBTTF-(ViRu)**_**2**_^**2+**^ as a mixed-valent system of Class II according to the scheme of Robin and Day with an unsymmetrical distribution of the second positive charge over the two {Ru} sites.^81^ Its electronic structure is, hence, best described as ({Ru}-CH=CH)^•+^-DBTTF^•+^-(CH=CH-{Ru}). This means that the second oxidation affects one of the {Ru} entities more strongly than the other and that the DBTTF bridge acts as an only moderately efficient electronic conduit, which is likely due to its large lateral extension.

In contrast to **DBTTF-(ViRu)**_**2**_^**•+**^, which persists for hours in solution with no perceptible changes, the corresponding dication has only limited stability. When **DBTTF-(ViRu)**_**2**_^**2+**^ was generated by treating **DBTTF-(ViRu)**_**2**_ with 2.1 equiv of 1,1′-diacetylferrocenium hexafluoroantimonate (*E*_1/2_^0/+^ = 490 mV), the same Ru(CO) and characteristic NIR bands (*vide infra*) as those in the spectroelectrochemical studies were observed (Figure S25). Monitoring by IR/NIR spectroscopy, however, indicated an irreversible loss of band intensities with a ca. 50% intensity decrease within 35 min at rt. Chemical lability is further amplified in the higher oxidized trication. Although the **DBTTF-(ViRu)**_**2**_^**2+/3+**^ redox couple is well-behaved on the voltammetric time scale, repeated attempts to generate this species either electrochemically inside our optically transparent thin-layer electrolysis cell or chemically resulted in rapid, irreversible spectral changes with a loss of CO band intensities. This unfortunately precludes its spectroscopic characterization.

**Figure 5:**
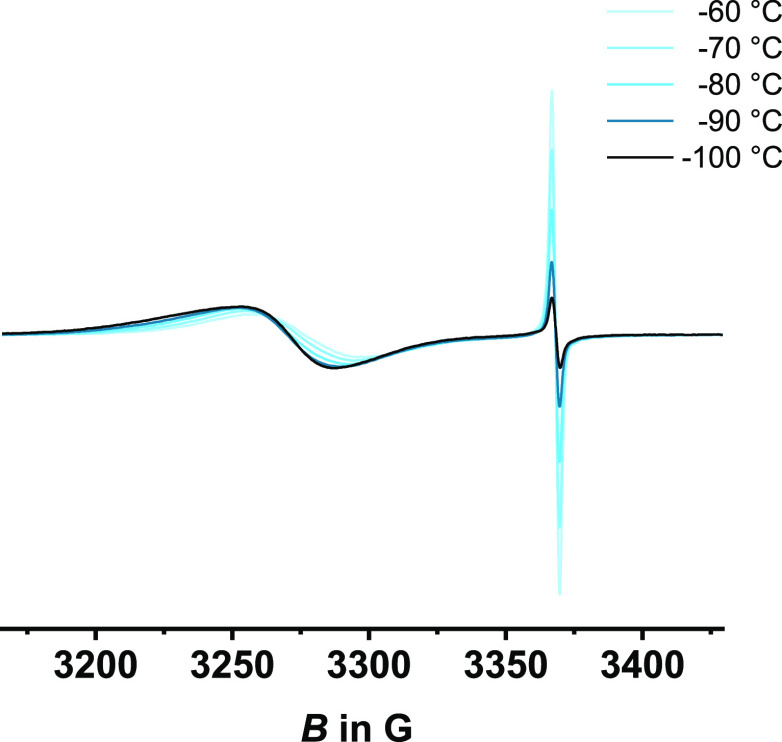
Temperature-dependent EPR spectra of **DBTTF-(ViRu)**_**2**_^**2+**^, chemically prepared by the oxidation of **DBTTF-(ViRu)**_**2**_ with 2.1 equiv of 1,1′-diacetylferrocenium hexafluoroantimonate at 0 °C.

EPR spectra of freshly prepared samples of the two-electron-oxidized species provided two separate resonance signals, as shown in Figure 5. The first isotropic signal at *g* = 2.009 is identical with that of **DBTTF-(ViRu)**_**2**_^**•+**^. The second one has a higher *g* value and is assigned to the {Ru}-CH=CH^•+^ entity. The latter resonance shows some positional drift from *g* = 2.058 at −60 °C to *g* = 2.070 at −80 °C and *g* = 2.071 in the frozen glass. Studies at higher temperatures were thwarted by the onset of degradation, setting in at −40 °C. Like for the **DBTTF-(ViRu)**_**2**_^**•+**^ cation, the intensity of the resonance assigned to the oxidized DBTTF linker gradually decreases upon cooling. As shown in Figure S24, the *T* induced changes are fully reversed upon rewarming, which excludes the possibility that the observed alterations result from chemical decomposition. As for the radical cation, the EPR signal of the DBTTF-based spin nearly vanishes at *T* = −150 °C in a frozen solvent matrix (Figure S24), possibly again as the result of dimerization via the DBTTF^•+^ constituent.

**Figure 6:**
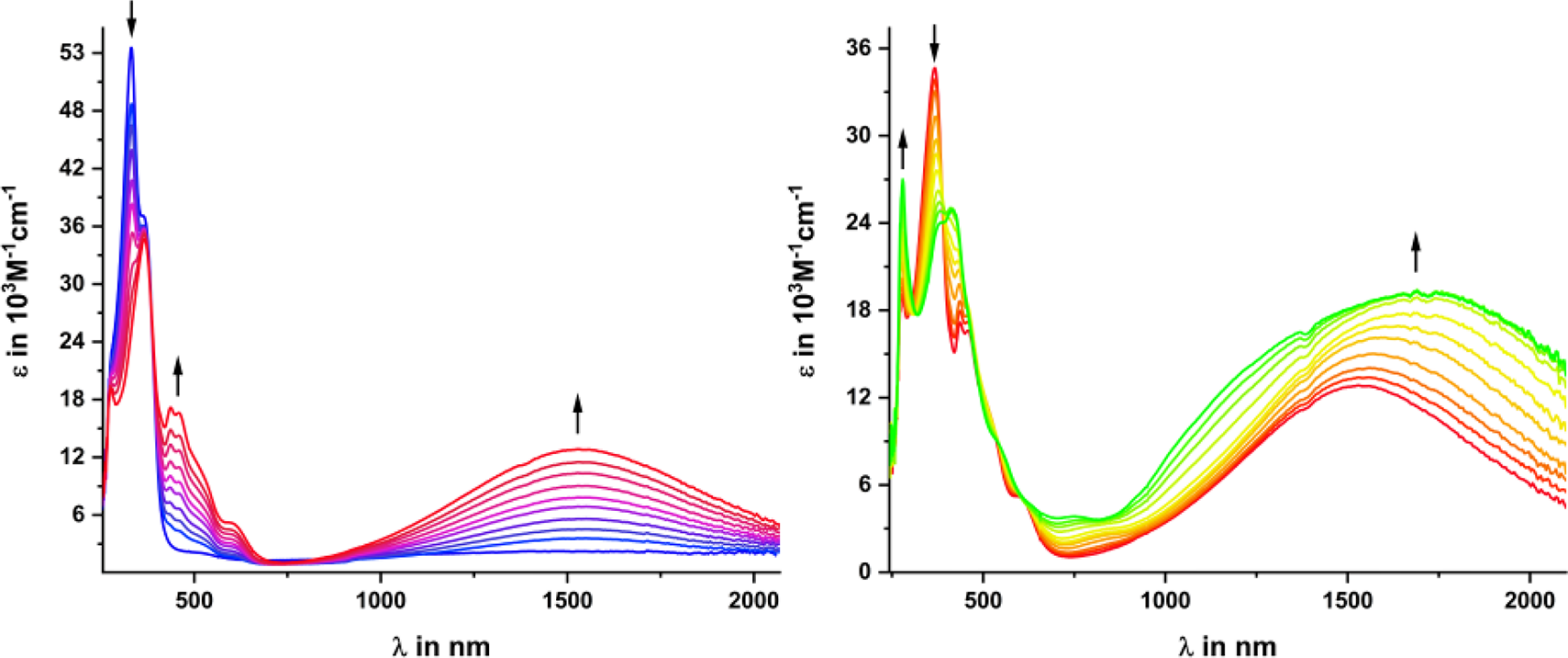
Changes in the UV/vis/NIR spectra upon the stepwise oxidation of **DBTTF-(ViRu)** to its monocation (left) and dication **DBTTF-(ViRu)**_**2**_^**2+**^ (right) in 1,2-C_2_H_4_Cl_2_/^*n*^Bu_4_N^+^PF_6_^–^ (0.2 M).

Revealing spectroscopic changes are also seen in the electronic spectra, as summarized in Figure 6. Neutral **DBTTF-(ViRu)**_**2**_ is characterized by two intense UV bands at 226 and 331 nm, both with distinct superimposed shoulders at higher wavelengths, and a low-energy tail that extends to ca. 540 nm. The latter is a characteristic asset of five-coordinated, 16-valence-electron alkenyl-{Ru} entities and results from d/d and alkenyl ligand-to-metal charge-transfer (LMCT) transitions, which target the d orbital that is directed toward the vacant coordination site opposite to the alkenyl ligand.^72,82^ A comparison with the UV/vis spectra of **DBTTF-I**_**2**_ and **DBTTF-(ATMS)**_**2**_ (Table 2 and Figures S26–S28) reveals a sizable red shift of the near-UV band and a partial loss of the band fine structure as the {Ru} entities are incorporated into the π-extended chromophore.

The first oxidation of **DBTTF-(ViRu)**_**2**_ is accompanied by a loss of the intensity and a red shift of the intense π → π* absorptions of the {Ru}-CH=CH-DBTTF-CH=CH-{Ru} chromophore and the emergence of a richly structured absorption feature with distinct peaks at 437 and 460 nm, as well as a weaker Vis band at 604 nm. A comparison with **DBTTF-(ATMS)**_**2**_^**•+**^ (Figure S29) and the reported spectrum of DBTTF^•+^^78^ signifies the structured Vis absorption as characteristic of the oxidized DBTTF chromophore and confirms the bridge character of the first oxidation in **DBTTF-(ViRu)**_**2**_. Particularly striking is the emergence of an intense absorption in the NIR at 1530 nm. This feature is also seen in the IR/NIR spectra, with a well-defined peak at 6440 cm^–1^ (1552 nm; Figure 4). It has no direct counterpart in **DBTTF-(ATMS)**_**2**_^**•+**^, which absorbs only weakly in that energy range (Figure S29 and S30) and must, hence, be connected to the appended vinyl-{Ru} entities.

During the second oxidation, the UV intensity decreases further, while the Vis and NIR absorption features broaden and intensify with concomitant shifts to higher or lower energies. The same behavior was also seen in the IR/NIR spectroelectrochemical experiments, as shown in Figure 4, where the main peak in the NIR now appears at 5840 cm^–1^ (1710 nm). The asymmetric band shape and an inflection at the higher-energy tail suggest that the NIR absorption of **DBTTF-(ViRu)**_**2**_^**2+**^ involves more than one electronic transition. This was verified by spectrum deconvolution (Figure S31).

In summary, our spectroscopic studies of **DBTTF-(ViRu)**_**2**_ and its oxidized forms indicate that the highest occupied molecular orbital (HOMO) of the neutral complex is dominated by the DBTTF linker with only limited contributions from the peripheral {Ru} entities and that a symmetric charge distribution is preserved after oxidation to **DBTTF-(ViRu)**_**2**_^**•+**^. The second oxidation then involves one of the {Ru} entities and results in an unsymmetric charge distribution over the two {Ru} sites, as verified by the presence of two separate CO stretches for the carbonyl ligands. **DBTTF-(ViRu)**_**2**_^**2+**^ is, hence, a moderately coupled mixed-valent system of Class II with a partially localized electronic ground state.

**Figure 7:**
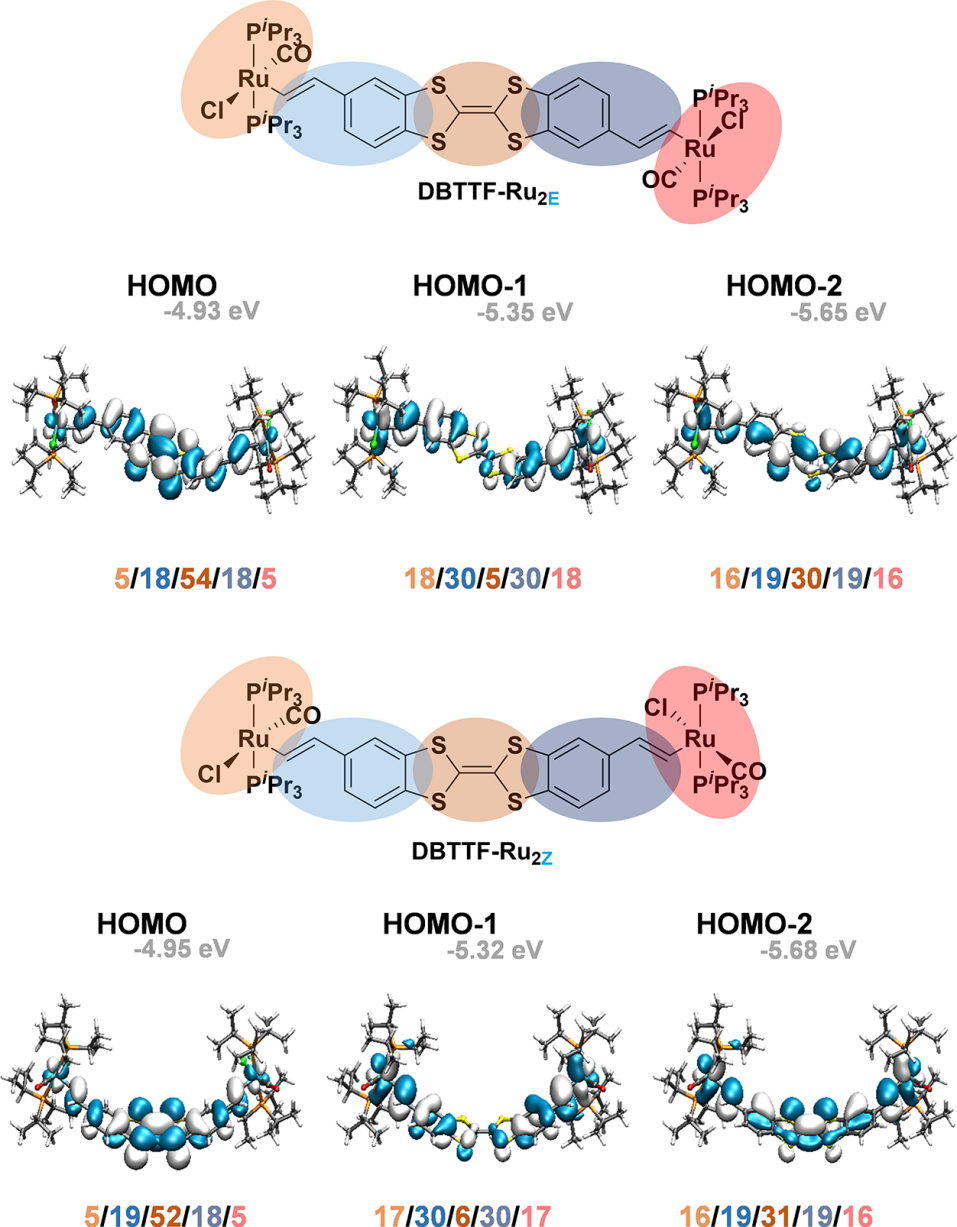
Contour diagrams of HOMO to HOMO–2 of **DBTTF-(ViRu)**_**2E**_ (top) and **DBTTF-(ViRu)**_**2Z**_ (bottom) along with fragment contributions.

Quantum-chemical calculations [density functional theory (DFT) at the pbe1pbe or M062X levels of theory] support our experimental findings and provide additional insight into the nature of the electronic structures and transitions of the neutral complex and its one- and two-electron-oxidized forms. Our computational studies considered the full models of both isomers, *E* and *Z*, and verified that the differences between them are negligible. The HOMO of **DBTTF-(ViRu)**_**2E/Z**_ spreads over the entire π-conjugated backbone, with dominant contributions from the DBTTF ligand, in particular, from its TTF core (54%/52%). Computed ruthenium contributions of 10% for both isomers agree with the observed small experimental blue shift of the Ru(CO) band during the first oxidation, which was matched by our calculations (1902 → 1908 cm^–1^). The lower-lying HOMO–1 and HOMO–2 either are based on the {Ru}-styryl-type subunits (96%/94% for the *E*/*Z* isomers) or are uniformly distributed over all constituents. Figure 7 provides contour diagrams of these molecular orbitals (MOs) of both isomers along with the contributions from the {Ru}, styryl, and central TTF entities, as computed by Mulliken analysis. Relevant numbers are collected in Tables S5–S7. The lowest unoccupied molecular orbital (LUMO) and LUMO+1 are shown in Figure S33.

Changes in the natural bond orbital (NBO)-calculated atomic partial charges and the evolution of the frontier spin orbitals are a means to follow the course of stepwise oxidations. Tables S8–S19 summarize the relevant data up to the dication level; graphical representations are provided as Figures S40–S45. Upon the first oxidation, 60%/64% of the computed charge loss come from the central TTF unit, while the annelated benzene rings and vinyl groups contribute 26%/24% and the {Ru} moieties 8%/6% each.

Dioxidized **DBTTF-(ViRu)**_**2**_^**2+**^ raises ambiguities about its electronic ground state, which could be a closed-shell singlet (S), a triplet (T), or an open-shell singlet (OSS). Calculations at the pbe1pbe level of theory indicate that the OSS state is energetically preferred by 1.8 kJ/mol (*E*) or 1.7 kJ/mol (*Z*) over the T state and by 7.5 or 17.2 kJ/mol over the S state. Such small energy differences suggest that all of these states may become thermally accessible and coexist at room temperature (rt). However, the pbe1pbe functional fails to reproduce the inherently unsymmetrical charge distribution, as derived from the experimentally observed pattern of two Ru(CO) stretching vibrations. For all three electronic states, only a single Ru(CO) band was computed. Differences of ca. 8 cm^–1^ for the computed CO stretches are significantly smaller than the observed band splitting of 27 cm^–1^, which implies that the pattern of two CO bands is unlikely rooted in different coexisting electronic states. Additional calculations on **DBTTF-(ViRu)**_**2E**_^**2+**^ with the BLYP35 functional, which proved successful for other mixed-valent compounds,^83,84^ as well as the CAM-B3LYP functional likewise produced centrosymmetric geometric structures and electronically equivalent {Ru} sites for the triplet state. The wB97XD, LC-wHPBE, and M062X functionals, however, led to noncentrosymmetric structures. The best qualitative agreement between experiment and theory was obtained for the M062X functional, which provided two equally intense CO bands spaced by 70 cm^–1^. Again, the open-shell states are energetically preferred over the singlet state, with the triplet state being 6.4 kJ/mol below the open-shell singlet and 18.5 kJ/mol below the singlet state. The latter two states possess symmetrical structures with equivalent bond parameters for the vinylruthenium moieties. Computed electronic spectra [pbe1pbe time-dependent DFT (TD-DFT) at the M062X-optimized structures] together with an analysis of the individual transitions based on electron density difference map (EDDM) plots and a comparison with the experimental data are provided as Figures S43–S45. Differences in the shapes and band intensities of calculated spectra from those computed with the pure pbe1pbe functional are only marginal.

TD-DFT calculations on neutral **DBTTF-(ViRu)**_**2E/Z**_ identify the two prominent Vis bands as π → π* transitions within the extended metal–organic chromophore. As indicated by the corresponding EDDMs in Figure S38, they are accompanied by a shift of the electron density from the TTF core and, to a varying extent, the {Ru} entities to the annulated benzene rings. The weak absorption near 520 nm is of LMCT origin with the degenerate in- and out-of-phase combinations of metal d orbitals (the LUMO and LUMO+1) that are directed toward the vacant coordination sites as the acceptor MOs.

**Figure 8:**
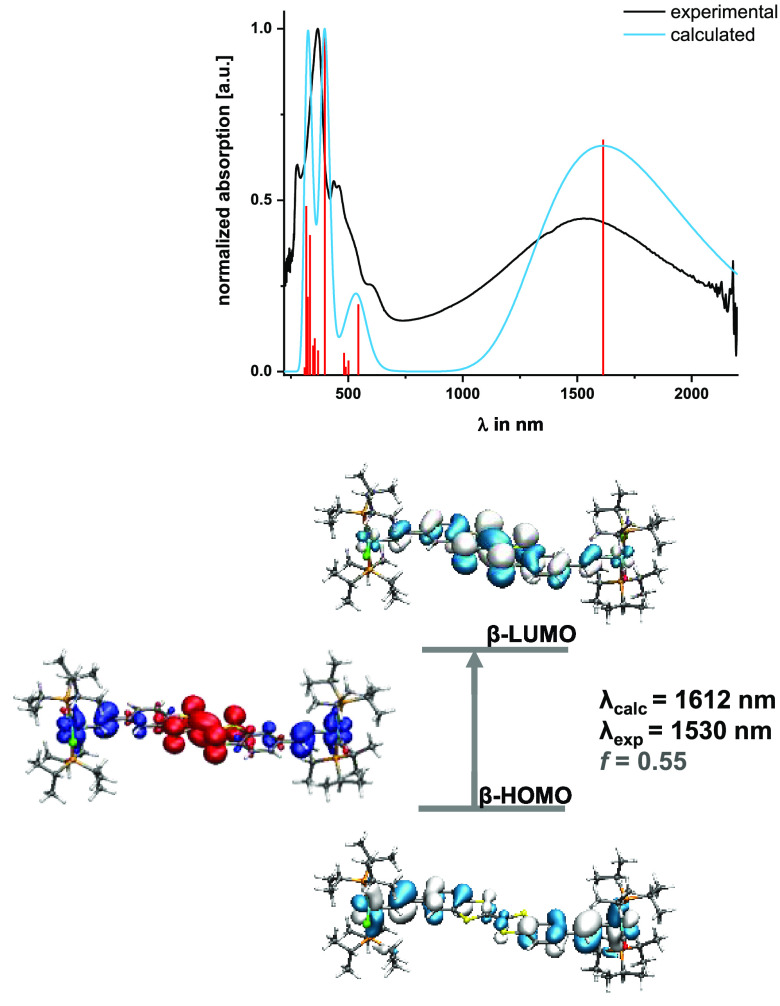
Top: Comparison of the experimental (black line) and pbe1pbe TD-DFT-computed (blue line) electronic spectrum of **DBTTF-(ViRu)**_**2E**_^**•+**^. Individual transitions are indicated by red bars. Bottom: Contour diagrams of the acceptor and donor MOs involved in the NIR transition along with the corresponding EDDM plot. Blue indicates an electron density decrease, and red indicates an electron density increase.

Our calculations reproduce the electronic spectra of **DBTTF-(ViRu)**^**•+**^ gratifyingly well. Figures 8 and S39 compare the experimental and TD-DFT-computed spectra and compile the MOs that contribute mainly to the individual excitations along with the EDDMs. The intense NIR band at a calculated wavelength λ_calc_ of 1612 nm (λ_exp_ = 1530 nm) corresponds to the β-HOMO → β-LUMO transition and has mixed intraligand (benzene → TTF) and {Ru} → TTF CT character with dominant electron flow from the {Ru}-CH=CH– groups. The Vis absorptions at 600 and ca. 460 nm (λ_calc_ = 544 and 501 nm) have the same character and also target the β-LUMO acceptor MO, with lower-lying π-MOs of mixed metal/ligand character as the donor MOs. The UV absorption retains its π → π* character but is associated with a higher degree of CT from the outer {Ru} sites compared to the neutral state.

**Figure 9:**
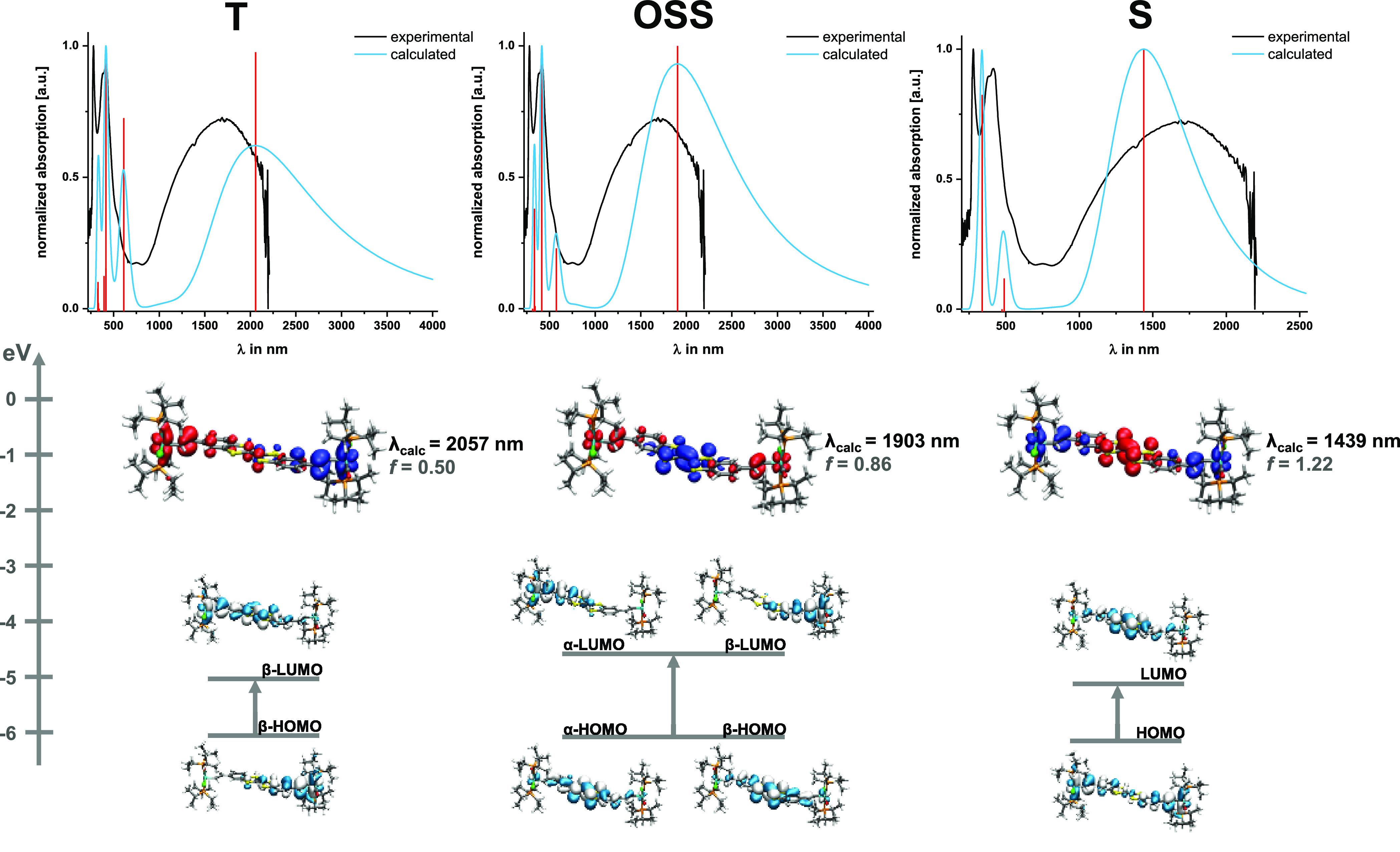
Top: Comparison of the experimental (black line) and pbe1pbe-M062X TD-DFT-computed (blue line) electronic spectra of **DBTTF-(ViRu)**_**2E**_^**2+**^ in the triplet state (T, left), open-shell singlet state (OSS, middle), and singlet state (S, right). Calculated transitions are indicated by red bars. Bottom: Contour diagrams of the acceptor and donor MOs involved in the NIR transition with the corresponding EDDMs. Blue indicates an electron density decrease, and red indicates an electron density increase.

The experimental spectra **DBTTF-(ViRu)**_**2**_^**2+**^ are equally well matched by the pbe1pbe-computed spectra employing the pbe1pbe- or M062X-optimized geometric structures. This holds particularly true for the energetically preferred triplet and open-shell singlet states, whereas those computed for the closed-shell singlet state lack the intense band near 415 nm (Figures 9 and S43–S45). The further discussion is based on the M062X computations because the predicted noncentrosymmetric structure of the T state matches with our experimental findings. For all electronic configurations, a low-energy transition located in the NIR with an oscillator strength higher than that for the radical cation is predicted. It is associated with CT involving the {Ru}-CH=CH– pendents but differs with respect to the identity of the donor and acceptor sites. In the S state, the {Ru}, styryl, and TTF moieties contribute almost equally to the second oxidation, so that the TTF core remains the primary acceptor and the NIR band is of metal-to-ligand charge-transfer (MLCT) origin. The T state is adequately described as having an oxidized TTF and one oxidized styrylruthenium moiety. Here, the NIR band results from intervalence charge transfer (IVCT) from the reduced to the oxidized styrylruthenium entity. In the OSS state, both styrylruthenium units are oxidized. In this electronic state, the TTF unit accumulates a lower positive charge than that in the radical cation, indicating a shift of the electron density from the periphery to the core during the second oxidation. This alters the character of the NIR band to LMCT.

**Figure 10:**
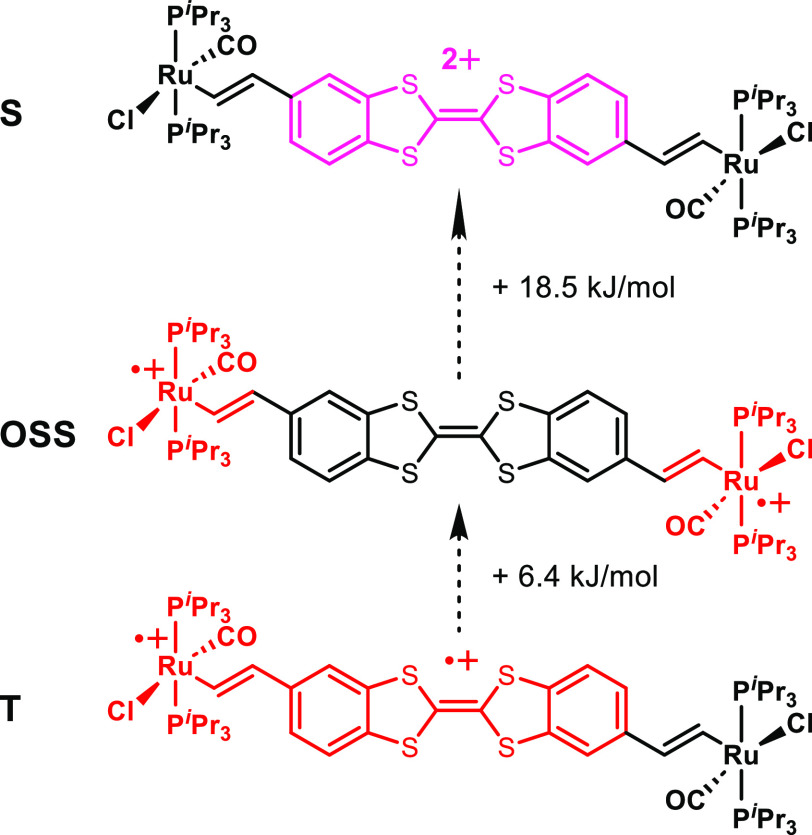
Schematic drawing of the charge assignments and M062X-computed energies of the triplet (T), open-shell singlet (OSS), and singlet (S) states of the **DBTTF-(ViRu)**_**2E**_^**2+**^ dication.

The triplet, open-shell singlet, and closed-shell singlet states can thus be viewed as representing the three conceivable alternative electronic descriptions of the dication shown in Figure 10: ({Ru}-CH=CH})^•+^-DBTTF^•+^-(CH=CH-{Ru}), ({Ru}-CH=CH})^•+^-DBTTF-(CH=CH-{Ru})^•+^, and ({Ru}-CH=CH})-DBTTF^2+^-(CH=CH-{Ru}). Revealingly, the calculated positions of the NIR band differ between 2057 nm (4850 cm^–1^) for the T state, 1903 nm (5260 cm^–1^) for the OSS state, and 1439 nm (6950 cm^–1^) for the S state. Spectral deconvolution of the NIR absorption indeed provided two separate NIR bands, a less intense one at 8750 cm^–1^ and a more intense one at 5930 cm^–1^, with their areas in the ratio of 1:3. The asymmetric, non-Gaussian shape of the NIR band might thus reflect coexisting electronic configurations for **DBTTF-(ViRu)**^**2+**^, which would agree with the small energy difference between them.

A similar dependence of the band assignment on the electronic configuration also applies to the Vis bands at 540 and 400 nm. Our TD-DFT data for the S state attribute the excitation at 500 nm to CT from the annelated benzene rings and the vinylruthenium moieties to the dioxidized TTF core such that they assume intraligand charge-transfer (ILCT) or MLCT character. In the OSS state, the identities of the donor and acceptor units are exactly reversed, which renders these bands LMCT and ILCT (TTF → Ph) in character. In the energetically preferred T state, however, all absorption bands are associated with charge transfer to acceptor orbitals, which are either delocalized over one vinylruthenium moiety and the DBTTF core or localized at one vinylruthenium moiety (Figures S43–S45).

In summary, our combined spectroscopic and quantum-chemical investigations on **DBTTF-(ViRu)**_**2**_^**•+**^ agree in assigning the first oxidation to the DBTTF ligand, in particular, its TTF substructure. Tokens are the small Ru(CO) band shift of only 6 cm^–1^, the close resemblence of the EPR spectrum to that of pristine **DBTTF**^**•+**^, the computed HOMO of **DBTTF-(ViRu)**_**2**_, the β-LUMO of **DBTTF-(ViRu)**_**2**_^**•+**^, and the changes in NBO-derived atomic charges concomitant with the first oxidation. Dioxidized **DBTTF-(ViRu)**_**2**_^**2+**^ is a very intricate system with all three conceivable electronic structures ({Ru}-CH=CH})^•+^-DBTTF^•+^-(CH=CH-{Ru}), ({Ru}-CH=CH})^•+^-DBTTF-(CH=CH-{Ru})^•+^, and ({Ru}-CH=CH})-DBTTF^2+^-(CH=CH-{Ru}) close in energy. The triplet state, resulting from oxidation of the TTF and one of the vinylruthenium entities, seems to be energetically preferred, which agrees with the experimental observation of two separate Ru(CO) bands. In this structure, the DBTTF bridge, despite its large lateral extension and despite being oxidized, still acts as a moderately efficient electronic conduit between the appended vinylruthenium termini, as is indicated by the modest splitting of the CO bands and the intense IVCT band.

### Reactions of **DBTTF-(ViRu)_2_** with Organic Acceptors

**Figure 11:**
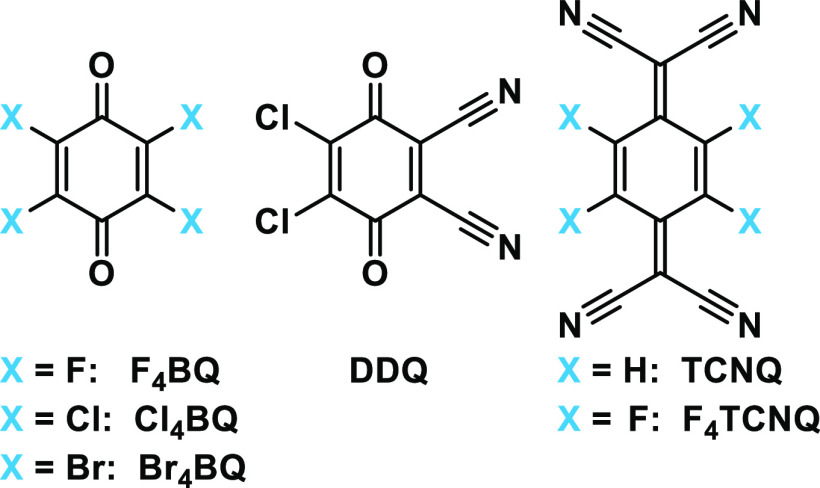
Structures of the organic acceptors employed in this study.

Its low first oxidation potential suggests that **DBTTF-(ViRu)**_**2**_ is a potent electron donor that can undergo redox reactions with suitable organic acceptors. We therefore treated **DBTTF-(ViRu)**_**2**_ with the tetrahalogeno-*p*-benzoquinones X_4_BQ^23,24^ (X = F, Cl, Br) and the related 2,3-dichloro-5,6-dicyano-1,4-benzoquinone (DDQ)^25,26^ as well as tetracyano-*p*-benzoquinodimethane TCNQ^2,27^ and its fluorinated derivative F_4_TCNQ.^2,28−30^ The chemical structures of the employed acceptors are listed in Figure 11. Their differing electron-accepting capabilities are reflected in their redox potentials, which, for the sake of consistency, were measured under the same conditions as those of **DBTTF-(ViRu)**_**2**_. These data are compiled in Table 1. Cyclic voltammograms of all employed acceptors are collected in Figures S18–S23. We also generated the associated one-electron-reduced forms of all employed acceptors by redox titrations (X_4_BQ, where X = F, Cl, Br) using decamethylferrocene, Cp*_2_Fe (*E*_1/2_ = −540 mV), as the reductant, or electrochemically (DDQ, TCNQ, and F_4_TCNQ) and collected their IR and UV/vis/NIR spectra in order to aid their identification in the as-formed products. These spectra are provided as Figures S46 and S47. Judging from the redox potentials, only the strongest acceptors DDQ and F_4_TCNQ should be able to quantitatively oxidize **DBTTF-(ViRu)**_**2**_ to its radical cation (Table 1). **DBTTF-(ViRu)**_**2**_ possesses a large cleft right above its DBTTF-based redox site, which is surrounded by hydrophobic walls defined by the P^*i*^Pr_3_ ligands, whose methyl protons are able to form stabilizing CH···X interactions with hydrogen-bond-accepting halogen or oxygen atoms of the acceptor (see Figure S13 for a space-filling model; note also that CH···π interactions were found in crystalline **DBTTF-(ViRu)**_**2**_). These and other attractive, noncovalent, interactions like S···X or π–π stacking may promote CT even in cases where the reduction potential of the acceptor falls below the oxidation potential of the donor.

In test reactions, equimolar quantities of the acceptor and donor were separately dissolved in dichloromethane and then mixed by slowly adding the solution of the respective acceptor to the donor. The resulting samples were analyzed by IR, UV/vis/NIR, and EPR spectroscopy in solution and, in the case of DDQ, F_4_TCNQ, and TCNQ, also in the solid state.

With tetrahalogeno-*p*-benzoquinones X_4_BQ (X = F, Cl, Br) as the acceptors, no change in the solution color was noted. IR and UV/vis/NIR spectra of the resulting solutions matched with the overlaid spectra of **DBTTF-(ViRu)**_**2**_ and the respective neutral X_4_BQ (Figures S48 and S49). In particular, no electronic transition at low energy that can be assigned to intermolecular CT from the donor to the acceptor was observed. EPR spectroscopy, which capitalizes on the high sensitivity of this method toward even traces of paramagnetic species, identified extremely weak resonances assignable to the **DBTTF-(ViRu)**_**2**_^**•+**^ radical cation and the X_4_BQ^•–^ radical anion for F_4_BQ and Cl_4_BQ. Curiously, a significantly more intense signal was observed for the reaction with Br_4_BQ (Figure S50), although its most negative reduction potential renders it the least likely candidate to oxidize **DBTTF-(ViRu)**_**2**_. Possibly, Br–S interactions promote CT to an extent that the two radical species can be observed by EPR spectroscopy while still escaping IR and UV/vis/NIR spectroscopic detection.

**Figure 12:**
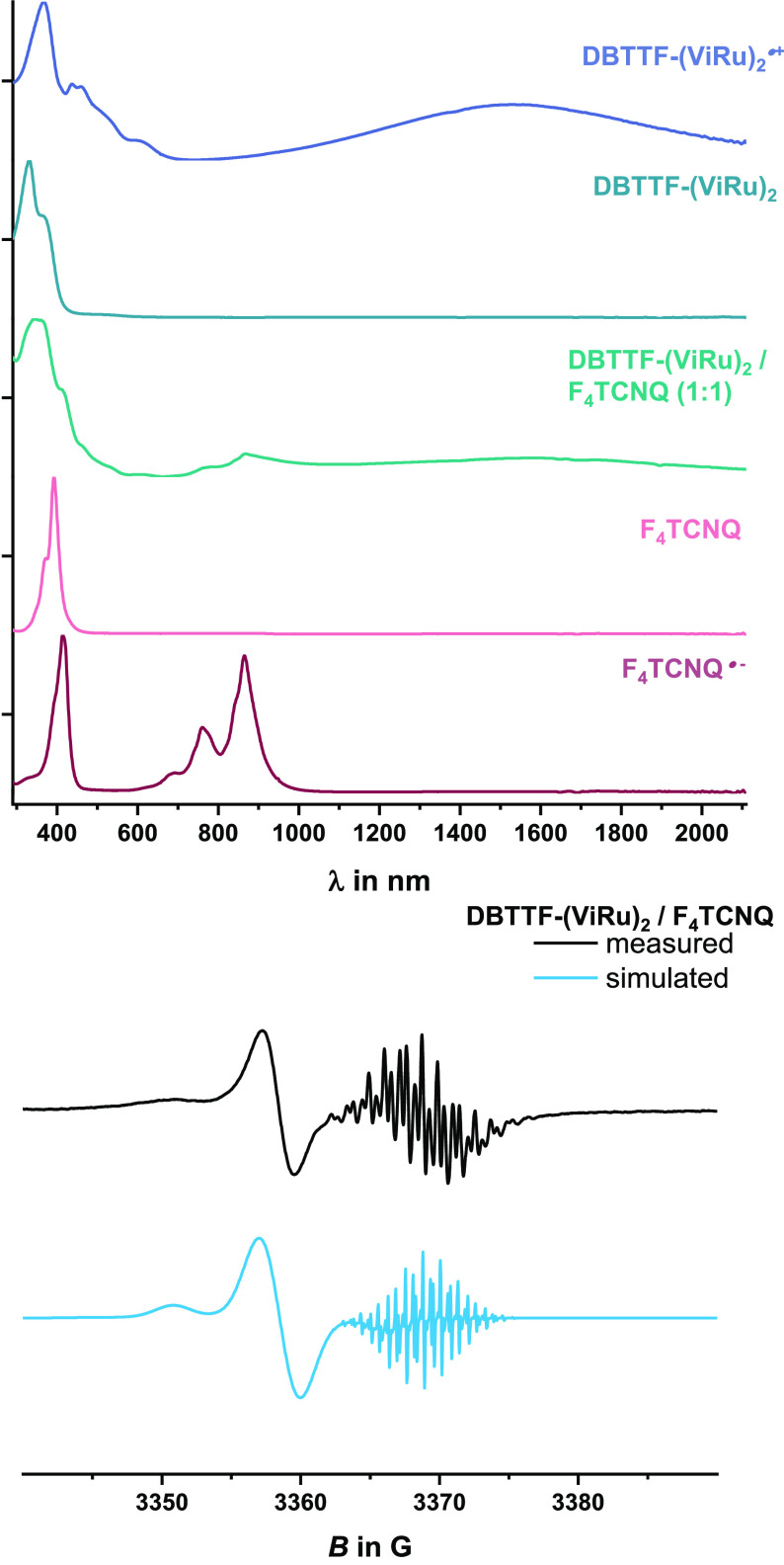
Top: Comparison of the UV/vis/NIR spectra of solutions of **DBTTF-(ViRu)**_**2**_, **DBTTF-(ViRu)**_**2**_^**•+**^, F_4_TCNQ, and F_4_TCNQ^*•–*^ and of a 1:1 mixture of **DBTTF-(ViRu)**_**2**_ and F_4_TCNQ in CH_2_Cl_2_. Bottom: Experimental (black line) and simulated (blue line) solution EPR spectra of **DBTTF-(ViRu)**_**2**_/F_4_TCNQ (1:1).

Mixing **DBTTF-(ViRu)**_**2**_ with any of the stronger acceptors DDQ, F_4_TCNQ, or TCNQ resulted in a rapid darkening of the solution and the formation of greenish-brown precipitates. IR and UV/vis/NIR spectra of the supernatant provided the spectroscopic fingerprints of the oxidized complex and the radical anion of the respective acceptor. Compilations of the spectra are shown in Figures 12 and S51–S55. Particularly diagnostic are the shifted C≡N stretching vibrations of the F_4_TCNQ^•–^ and DDQ^•–^ anions, the loss of the quinone CO band of neutral DDQ, the characteristic electronic absorptions of the reduced acceptor, and the appearance of the pair of blue-shifted Ru(CO) bands and a NIR band. For DDQ and F_4_TCNQ, the NIR band is intense and unshifted with respect to that of pristine **DBTTF-(ViRu)**_**2**_^**•+**^. Solution EPR spectra featured separate resonances of both paramagnetic constituents, the **DBTTF-(ViRu)**_**2**_^**•+**^ radical cation and the F_4_TCNQ^•–^ or DDQ^•–^ radical anion, the latter with their characteristic *A*(^14^N) and, in the case of F_4_TCNQ^•–^, *A*(^19^F) hfs; *g* values and hfs constants are collected in Table 3. In the case of the weaker acceptor TCNQ, the NIR band is significantly less intense, and its maximum is blue-shifted to ca. 960 nm (Figure S53). Moreover, solution EPR spectra show only a single broadened resonance signal with ill-resolved hfs lines at *g* = 1.993, suggesting a more dynamic situation with incomplete CT (Figure S51).

**Table 3:** EPR Data for the Species Formed upon Reaction of **DBTTF-(ViRu)**_**2**_ with Equimolar Amounts of Br_4_BQ, DDQ, F_4_TCNQ, and TCNQ in CH_2_Cl_2_ at rt^a^

	*g*_iso_	*A*(^32^S)	*A*(^99/101^Ru)	*A*(^31^P)	*A*(^14^N)	*A*(^35^Cl)	*A*(^19^F)
**DBTTF-(ViRu)**_**2**_^**•+**^	2.000	4.3 (4)	0.9 (2)	0.5 (4)	-	-	-
**DDQ**^**•–**^	1.996	-	-	-	0.9 (2)	0.6 (2)	-
**DBTTF-(ViRu)**_**2**_^**•+**^	2.000	6.4 (4)	2.7 (2)	0.9 (4)	-	-	-
**F**_**4**_**TCNQ**^**•–**^	1.993	-	-	-	1.2 (4)	-	0.7 (4)
**DBTTF-(ViRu)**_**2**_^**•+**^ b,c	1.993	-	-	-	-	-	-
**TCNQ**^**•–**^	1.993	-	-	-	-	-	-
**DBTTF-(ViRu)**_**2**_^**•+**^ c	2.012	-	-	-	-	-	-
**Br**_**4**_**BQ**^**•–**^	1.996	-	-	-	-	-	-

a hfs constants *A* are given in Gauss, with the number of identical nuclei in parentheses.

b Averaged spectrum.

c Unresolved hfs.

Attenuated-total-reflection IR and UV/vis/NIR absorption spectra recorded of the solid materials deposited during the reaction are also indicative of the presence of ionic constituents. The results agree with those in solution; however, only unstructured EPR signals without any resolution into separate subspectra or resolved hfs are observed under these conditions (Figure S52). Stability tests with periodic recording of the spectra indicated that the solid samples are bench-stable for at least 3 weeks. The results are collected as Figures S56 and S57.

Unfortunately, all of our attempts to obtain single crystals of any of these products failed. We therefore conducted scanning electron microscopy (SEM) and powder X-ray diffraction (pXRD) experiments in an attempt to obtain further information about the morphology and crystallinity of the solid materials. The pXRD spectra of the three CT compounds showed no defined reflections (Figures S58–S60). This indicates that the obtained materials are amorphous. SEM measurements showed porous agglomerates of nearly monodisperse spherical particles of ca. 0.3–0.5 μm diameter. The corresponding data are collected in Figures S61–S63.

Compressed pellets of the washed precipitates were investigated in a miniaturized setup with micrometer-sized tungsten probe tips as electrodes (see the Supporting Information for details). No significant current flow was observed even when the tip electrodes were positioned in close proximity, between 10 and 15 μm apart, and a gate voltage of up to 20 V was applied (Figure S65).

## Summary and Conclusions

We have presented bis(alkenylruthenium) complex **DBTTF-(ViRu)**_**2**_ with a redox-active, π-extended DBTTF linker. The complex and its organic precursors exist as *E* and *Z* isomers, which differ with respect to the positioning of the carbon atoms to which the vinyl linkers are attached, yet with indiscernible electrochemical and spectroscopic properties. **DBTTF-(ViRu)**_**2**_ is electron-rich and undergoes four consecutive one-electron oxidations, the first three of which are chemically reversible on the CV time scale. We employed IR/NIR, UV/vis/NIR, and EPR spectroscopic as well as computational studies in order to identify the redox sites involved in the individual oxidations and to delineate the electronic structures of the various oxidized forms. Due to stability issues, this was, however, only possible up to the level of the dication. According to our results, the first oxidation is centered on the TTF core structure of the DBTTF ligand. Dioxidized **DBTTF-(ViRu)**_**2**_^**2+**^ has an inherently unsymmetrical charge distribution, as inferred from the presence of two distinct Ru(CO) bands with a splitting of 27 cm^–1^ in the IR. This points to an electronic structure where the DBTTF ligand and one of the vinylruthenium subunits are oxidized, i.e., ({Ru}-CH=CH})^•+^-DBTTF^•+^-(CH=CH-{Ru}). This electronic configuration, rendering a triplet state with two structurally different vinylruthenium moieties, was indeed found to be slightly preferred over the symmetrical open-shell singlet state with two oxidized vinylruthenium entities and the singlet state, where the DBTTF core is oxidized twice. All of these states were found to be energetically close within only 19 kJ/mol and might therefore coexist.

Treatment with the strong organic acceptors F_4_TCNQ, DDQ, and TCNQ and, to a minor extent, also with Br_4_BQ, generates samples that contain the **DBTTF-(ViRu)**_**2**_^**•+**^ radical cation alongside the radical anion of the organic acceptor. Solids obtained from these mixtures proved to be highly amorphous and behave as electrical insulators.

## Supporting Information Available

Experimental details, procedures, and data, including NMR and mass spectra, cyclic and square-wave voltammetry, crystallographic data, spectroelectrochemical measurements, IR, EPR, and electronic spectra, quantum-chemical calculations, pXRD, and conductivity measurements on the CT salts (PDF)
