# Supplementary material for: A Dibenzotetrathiafulvalene-Bridged Bis(alkenylruthenium) Complex and Its One- and Two-Electron-Oxidized Forms
Source: Inorg Chem. 2023 Nov 3;62(46):18789–803. doi: 10.1021/acs.inorgchem.3c03184 (PMC10664072; doi:10.1021/acs.inorgchem.3c03184)
Supplement: Supplementary file 1 — ic3c03184_si_001.pdf [file ic3c03184_si_001.pdf]

Supporting Information for

A Dibenzotetrathiafulvalene-Bridged Bis(alkenylruthenium) Complex and Its One- and Two-Electron Oxidized Forms

Franciska S. Gogesch,[a] Lukas S. Laininger,[a] Nick Sokov,[a] Stefan M. Schupp,[b] Laura Senft,[c] Hipassia M. Moura,[a] Michael Linseis,[a] Lukas Schmidt-Mende,[b] Ivana Ivanović-Burmazović,[c] Miriam M. Unterlass,[a] Rainer F. Winter[a]*

[a] F. S. Gogesch, L. S. Lainiger, N. Sokov, Dr. H. M. Moura, Dr. M. Linseis, Prof. Dr. M. M. Unterlass, Prof. Dr. R. F. Winter
Fachbereich Chemie
Universität Konstanz
Universitätsstraße 10, 78457 Kostanz (Germany)
E-mail: rainer.winter@uni-konstanz.de

[b] S. M. Schupp, Prof. Dr. L. Schmidt-Mende
Fachbereich Physik
Universität Konstanz
Universitätsstraße 10, 78457 Konstanz

[c] L. Senft, Prof. Dr. I. Ivanović-Burmazović
Department Chemie
Ludwig-Maximilians-Universität München
Butenandstraße 5-13, Haus D, 81377 München (Germany)

Table of Content

Experimental Methods and Materials S2
Synthesis Details S3
NMR Spectra S5
Electrochemistry S17
EPR Spectra S21
Electronic Spectra S22
Quantum Chemistry S24
Electronic Spectra of Acceptors S41
Redox Reactions between DBTTF(ViRu)$_2$ and the Organic Acceptors S43
Atomic Coordinates for Geometry-Optimized Structures S54
References S99

Experimental Methods and Materials

All manipulations were performed under purified nitrogen atmosphere with dry, distilled, and nitrogen-saturated solvents. All reagents were purchased from commercial vendors and used without further purification. ^{1}H NMR (500 MHz) and ^{13}C NMR (126 MHz) spectra were measured on a Jeol ECZ 500R spectrometer, ^{1}H NMR (800 MHz) and ^{13}C NMR (202 MHz) spectra on a Bruker Avance Neo 800 and ^{31}P NMR (162 MHz) spectra were measured on a Bruker Avance III 400 spectrometer.the precursor **DBTTF-I$_2$** was prepared following the literature procedure.[1]
Mass spectra of organic compounds were recorded on a LTQ Orbitrap Velos (Thermo Scientific, Konstanz, Germany) in positive mode with ESI capable of a resolution of at least 60000 in CH_2Cl_2 as solvent. The spectrometer was calibrated with the Pierce LTQ Velos ESI Positive Calibration Solution (Thermo Fisher Scientific) prior to every measurement. Mass spectra of **DBTTF-(ViRu)$_2$** were determined by an *UHR-TOF Bruker Daltonik maXis plus* ESI-quadrupole time-of-flight (qToF) spectrometer (München, Germany). The resolution is at least 60000 FWHM. The detection was performed in positive ion mode with a source voltage of 3.5 kV and a flow rate of 180 µL/h. Nitrogen was used as drying gas. For solvent removal, the temperature was 180 °C and the temperature of the spray gas was 20 °C. The calibration was done prior to each measurement by direct infusion of low concentrated *Agilent ESi-TOF* tuning mixture. This gave an m/z range of singly charged peaks of up to 2700 Da.

Electrochemical and Spectroelectrochemical Measurements

All electrochemical experiments were performed in a custom-made cylindrical vacuum-tight single-compartment cell. A coiled Pt wire and a coiled Ag wire as the counter and reference electrodes were sealed into glass capillaries and were introduced *via* quickfit screws at opposite sides of the cell. A platinum electrode was used as the working electrode and inserted through the top port *via* a teflon screw cap with a suitable fitting. The working electrode was polished first with 1 µm and then 0.25 µm diamond paste (Buehler-Wirtz) before measurements. The cell can be attached to a conventional *Schlenk* line *via* a side arm equipped with a teflon screw valve and allows experiments to be performed under an atmosphere of argon with approximately 5 mL of analyte solution. CH_2Cl_2 / $^{n}Bu_4NPF_6$ (0.1 M) was used as the supporting electrolyte. Referencing was done by adding an equimolar amount of cobaltocenium hexafluorophosphate as an internal standard to the analyte solution after all data of interest had been collected. Representative sets of scans were repeated with the added standard. The final referencing was done against the ferrocene/ferrocenium ($Cp_2Fe^{0/+}$) redox couple with $E_{1/2}$ ($Cp_2Co^{+/0}$) = −1330 mV vs $Cp_2Fe^{0/+}$ for CH_2Cl_2 / $^{n}Bu_4NPF_6$. Electrochemical data were acquired with a computer-controlled BASi potentiostat.

The OTTLE cell for spectroelectrochemical experiments was also self-constructed and follows that the design of Hartl *et al*.[2] It comprises of Pt-mesh working and counter electrodes and a thin silver wire as a pseudoreference electrode. The electrodes are sandwiched in between two CaF_2 windows of a conventional liquid IR cell. The working electrode was positioned in the center of the spectrometer beam. The required potential was applied by connecting the cell leads to a Wenking POS 2 potentiostat by Bank Elektronik-Intelligent Controls GmbH. As supporting electrolyte, a 0.2 M solution of $^{n}Bu_4NPF_6$ in 1,2-$C_2H_4Cl_2$ was used. FT-IR spectra were recorded using a Bruker Tensor II FT-IR spectrometer. UV/Vis/NIR spectra were acquired on a TIDAS fiber optic diode-array spectrophotometer (combined MCS UV/NIR and PGS NIR instrumentation) from j&m Analytik AG.

EPR spectra were obtained using a MiniScope MS 400 and a MiniScope MS5000 X-band tabletop spectrometer by magnettech GmbH. All measurements were performed at room temperature. Samples of the oxidized complex were generated by using ferrocenium hexafluorophosphate (monocation) or 2.1 equiv. of diacetylferrocenium hexafluoroantimonate (dication) as oxidants. Simulation of experimental EPR spectra was performed with the MATLAB EasySpin program.[3]

Computational Details

The ground state electronic structures of the full models of all compounds were calculated by density functional theory (DFT) methods using the Gaussian 16 program packages.[4] Open shell systems were calculated by the unrestricted Kohn-Sham approach (UKS). Geometry optimization followed by vibrational analysis was performed in solvent media. Solvent effects were described by the polarizable continuum model (PCM) with standard parameters for 1,2-dichloroethane.[5,6] Electronic spectra were calculated by the TD-DFT method on optimized geometries. The 6-31G(d) polarized double-ζ basis sets[7] were employed for all atoms together with the Perdew, Burke, Ernzerhof exchange and correlation functional (pbe0pbe).[8,9] Additional geometry optimization calculations were performed with M06-2X[10] with the electronic spectra being calculated on optimized geometries by the TD-DFT method decribed above. The GaussSum program package was used to analyze the results,[11] while the visualization of the results was performed with the Avogadro program package.[12] Graphical representations of molecular orbitals were generated with the help of GNU Parallel[13] and plotted using the vmd program package[14] in combination with POV-Ray.

X-Ray Crystallography

X-Ray diffraction analysis was performed on a *STOE IPDS II Diffractometer* with a graphite-monochromator, and a MoKα-radiation source (λ = 0.71073 Å) at a scan rate of 3 - 30° min^{-1} in ω at T = 100.15 K. Using *Olex2*[15], the structure was solved with the *ShelXT*[16] structure solution program and refined with ShelXL[17] refinement package using the *Full-Matrix-Least-Squares* method. For visualization, the *Platon*[18] and *Mercury*[19] programs were used.

Powder X-Ray Diffraction

Powder X-ray diffraction (PXRD) measurements were carried out with a BrukerD8 Discover device equipped with a 1 µS CuK_{α} microfocus X-ray source and an energy dispersive LynxEye detector.the sample holder was oscillated during the measurement, and the samples were measured from 5° ≤ 2θ ≤ 70°.

Scanning Electron Microscopy

Scanning Electron Microscopy (SEM) was performed for some of the samples. Prior to imaging, the powders were sprinkled over a carbon tape, and the samples were coated by sputtering with a 17 nm thick layer of Au/Pd. The images were acquired in a field emission scanning electron microscope (SEM) Analytic SEM Zeiss Gemini 500 microscope, in the secondary electron mode, using 5 kV acceleration and 4.9 mm working distance.

Conductivity Measurements

The solid charge-transfer compounds were placed on a conductive Cu-tape, which was kept on a scanning electron microscope (SEM) stamp. Micrometer-sized tungsten probe tips were used as electrodes. Additionally, for **DBTTF-(ViRu)$_2$** / F_4TCNQ a second setup was used, whereby the solid was placed between gold electrodes with a distance of 10 µm. For fixation, the solid was compressed using a glass wafer. During the measurements, the applied voltage was varied from -20 to 20 V using a Keithley 2401.

Synthesis Details

5,5'-/5,6'-Bis(trimethysilylethynyl)-dibenzotetrathiafulvalene, DBTTF-(ATMS)$_2$

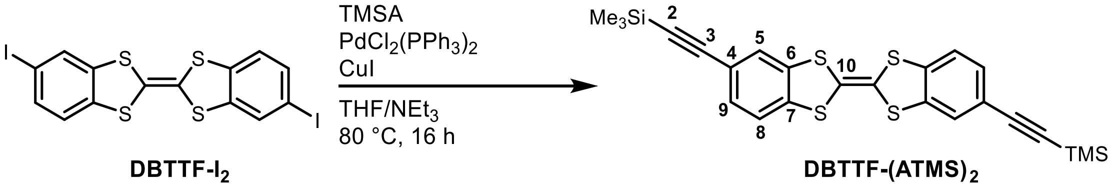

Diiododibenzotetrathiafulvalene[1] (299.0 mg, 0.54 mmol, 1.00 eq.) was suspended in THF (10 mL) and NEt_3 (10 mL). TMSA (0.29 mL, 194.4 mg, 2.00 mmol, 3.70 eq.), $PdCl_2(PPh_3)_2$ (19.0 mg, 0.03 mmol, 0.05 eq.) and CuI (5.1 mg, 0.03 mmol, 0.05 eq.) were added. The reaction mixture was stirred at 80 °C overnight. After cooling to room temperature, the reaction mixture was diluted with CH_2Cl_2 (50 mL) and washed with H_2O (3 x 50 mL). The organic phase was dried over $MgSO_4$ and the solvent was removed under reduced pressure. The yellow residue was washed with *n*-hexane (4 x 15 mL) to yield **DBTTF-(ATMS)$_2$** (218.0 mg, 0.44 mmol, 81 %) as a yellow solid.

^{1}H NMR (500 MHz, $CDCl_3$): δ [ppm] = 7.34 -7.32 (m, 2H, **H-5**), 7.21 (dd, 2H, $^3J_{HH}$ = 8.2 Hz, $^4J_{HH}$ = 1.4 Hz, **H-9**), 7.16 (d, 2H, $^3J_{HH}$ = 8.2 Hz, **H-8**), 0.24 (s, 18H, **H-1**). **^{13}C{^{1}H} NMR** (126 MHz, $CDCl_3$): δ [ppm] = 137.5 (s, **C-7**), 137.3 (s, **C-6**), 130.1 (s, 2C, **C-9**), 125.2 (s, **C-5**), 121.8 (s, **C-8**), 121.5 (s, **C-4**) 111.2 (s, **C-10**), 104.1 (s, 1C, **C-3**), 95.9 (s, **C-2**), 0.3 (s, **C-1**).

5,5'-/5,6'-Diethynyl-dibenzotetrathiafulvalene, DBTTF-(AH)$_2$ and DBTTF-(ViRu)$_{2E/Z}$

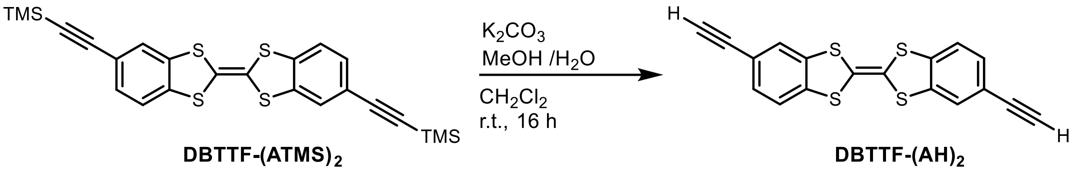

DBTTF-(ATMS)$_2$ (50.0 mg, 0.10 mmol, 1.00 eq.) was dissolved in CH_2Cl_2 (12 mL) and MeOH (5 mL). K_2CO_3 (240.0 mg, 1.74 mmol, 17.40 eq.) and a few drops of H_2O (0.5 mL) were added. The reaction mixture was stirred at room temperature overnight. During the reaction a yellow precipitate was formed. The supernatant solution was removed and the yellow solid was washed with H_2O (3 × 15 mL) and CH_2Cl_2 (15 mL) and dried *in vacuo*. The title compound (35.2 mg, 0.10 mmol) was obtained as a yellow solid in quantitative yield and used in the next step without further purification.

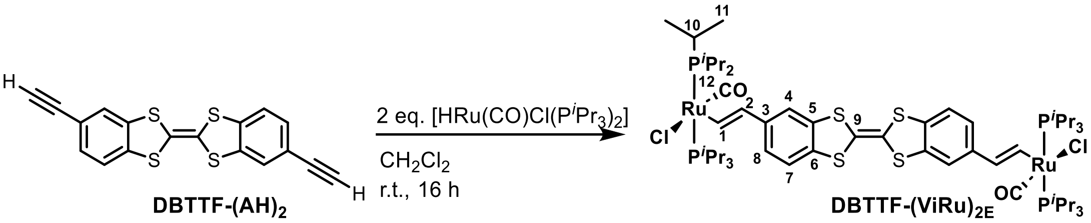

DBTTF-(AH)$_2$ (35.5 mg, 0.10 mmol, 1.00 eq.) was suspended in CH_2Cl_2 (10 mL) and was added dropwise to a solution of [HRu(CO)Cl(P^{i}Pr$_3$)$_2$] (99.0 mg, 0.20 mmol, 2.00 eq.) in CH_2Cl_2 (10 mL). The mixture turned red while stirring at room temperature overnight. The solvent was removed under reduced pressure. The resulting red solid was washed with *n*-hexane (2 × 15 mL) and MeOH (2 x 15 mL) and dried *in vacuo*. **DBTTF-(ViRu)$_2$** was obtained as an E/Z isomeric mixture (5,5'- and 5,6' isomers) in a yield of 70% (92.7 mg, 0.07 mmol).

^{1}H NMR (800 MHz, C_6D_6): δ [ppm] = 8.85 (d, 2H, $^3J_{HH}$ = 13.4 Hz, **H-1**), 6.75 (s, 2H, **H-8$_Z$**), 6.74 (s, 2H, **H-8$_E$**), 6.70 (vt, 2H, **H-7**), 6.60 (d, 2H, $^3J_{HH}$ = 8.2 Hz, **H-4$_E$**), 6.58 (d, 2H, $^3J_{HH}$ = 8.2 Hz, **H-4$_Z$**), 6.01 - 6.05 (m, 2H, **H-2**), 2.50 - 2.43 (m, 12H, **H-10**), 1.05 - 1.00 (m, 72H, **H-11**).
^{13}C{^{1}H} NMR (202 MHz, C_6D_6): δ [ppm] = 203.3 (t, $^2J_{CP}$ = 12.9 Hz, **C-12**), 152.1 (t, $^2J_{CP}$ = 10.5 Hz, **C-1**), 137.6 (s, **C-5**), 136.9 (s, **C-3**), 133.3 (s, **C-2**), 131.7 (s, **C-6**), 121.9 (s, **C-7**), 121.6 (s, **C-8**), 117.2 (s, **C-4**), 111.2 (s, **C-9**), 24.5 (vt, **C-10**), 19.6 (d, $^1J_{CP}$ = 52.9 Hz , **C-11**). **^{31}P{^{1}H} NMR** (162 MHz, C_6D_6): δ [ppm] = 38.5 (s).

Elemental Analysis *calcd.* for $C_{56}H_{94}Cl_2O_2P_4Ru_2S_4$: C, 50.78%, H, 7.17%; found: C, 49.98%, H, 6.98%.
ESI MS (CH_2Cl_2) 1324.559 m/z ($[M]^+$, calculated 1324.2525 m/z), 663.1274 m/z ($[M]^{2+}$, calculated 663.1272 m/z).

NMR Spectra

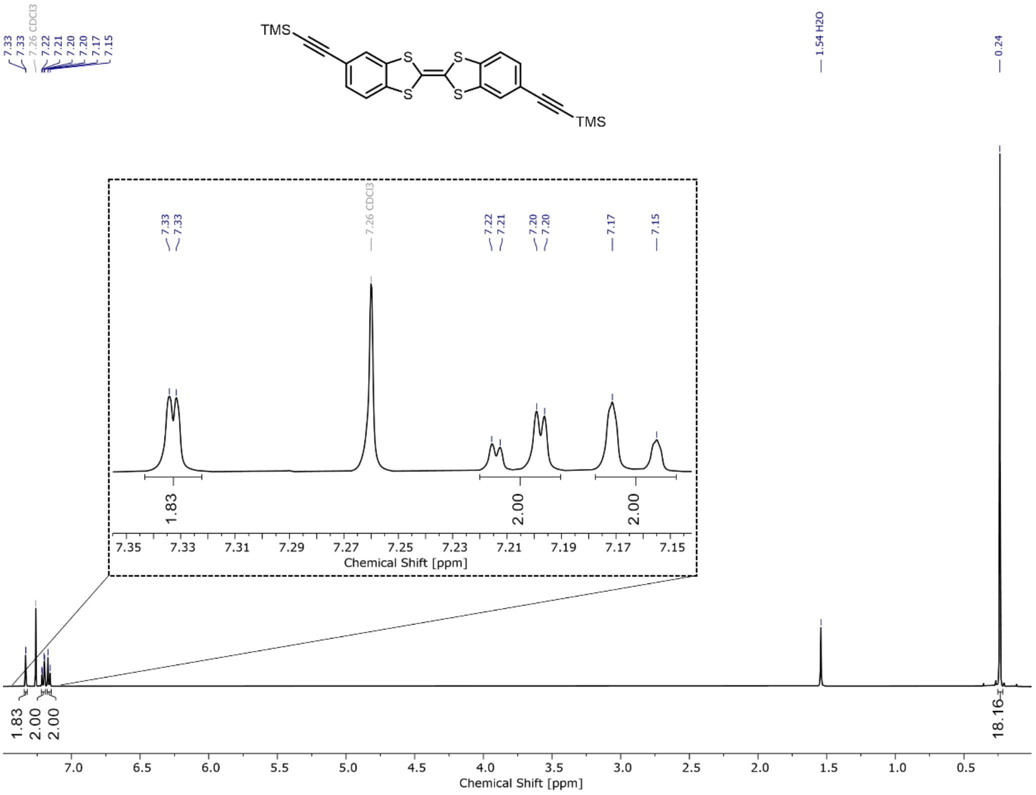

Figure S1. ^{1}H NMR spectrum of **DBTTF-(ATMS)$_2$** in $CDCl_3$.

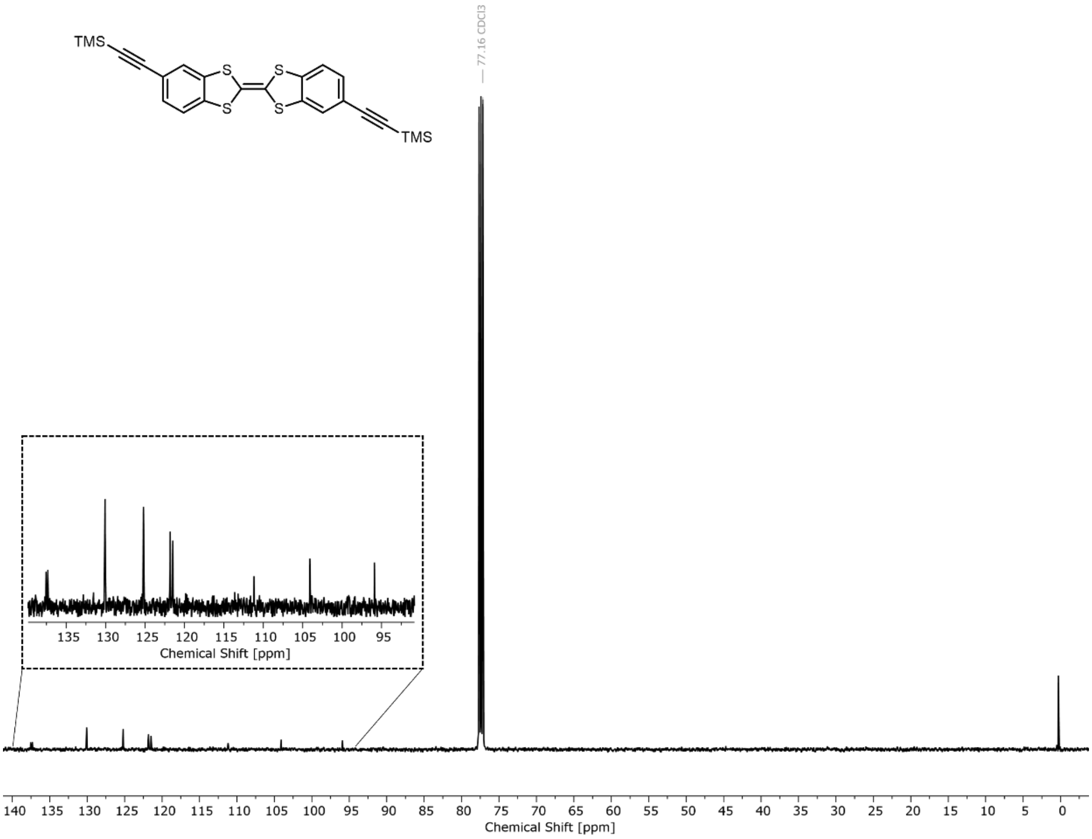

Figure S2. $^{13}C\{^{1}H\}$ NMR spectrum of **DBTTF-(ATMS)$_2$** in $CDCl_3$**.**

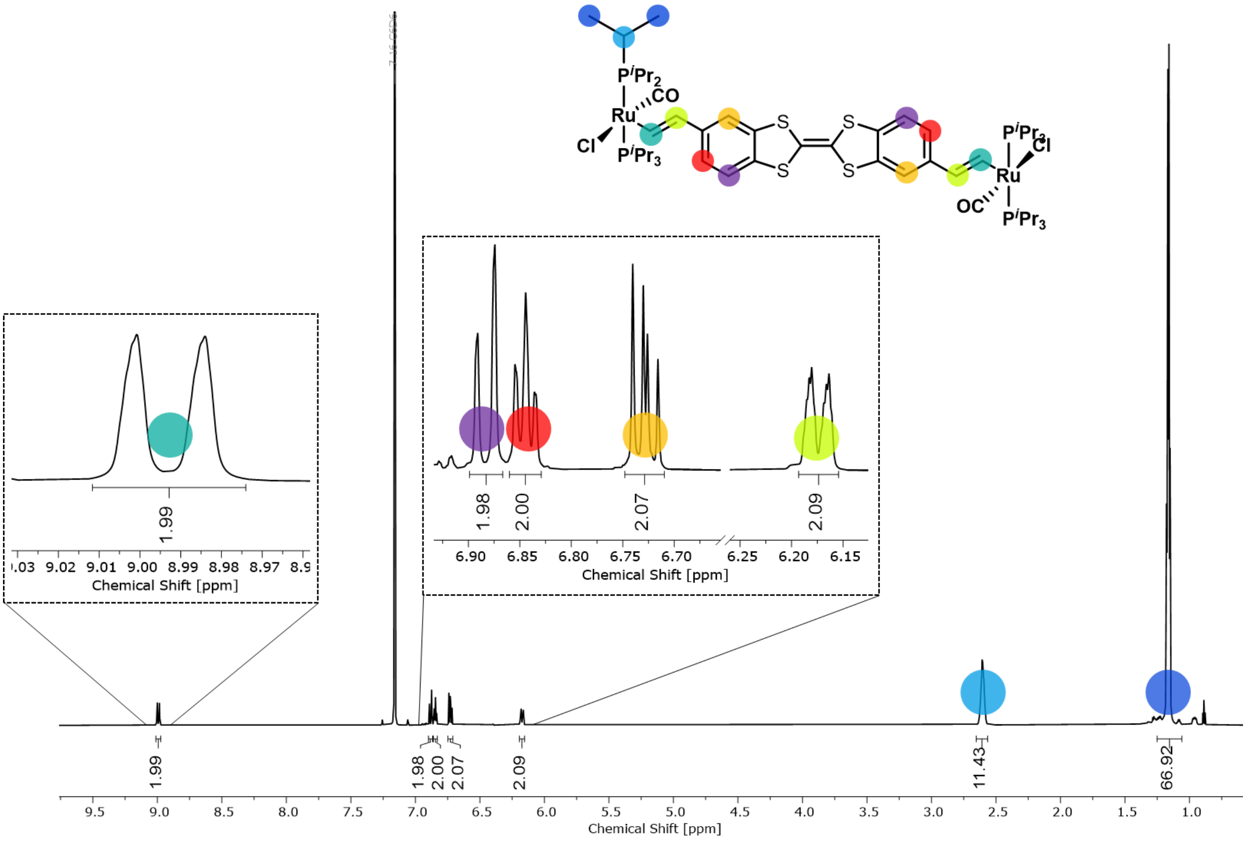

Figure S3. ^{1}H NMR spectrum (800 MHz) of an *E*/*Z* isomeric mixture **DBTTF-(ViRu)$_2$** in C_6D_6 with assignment of the resonance signals.

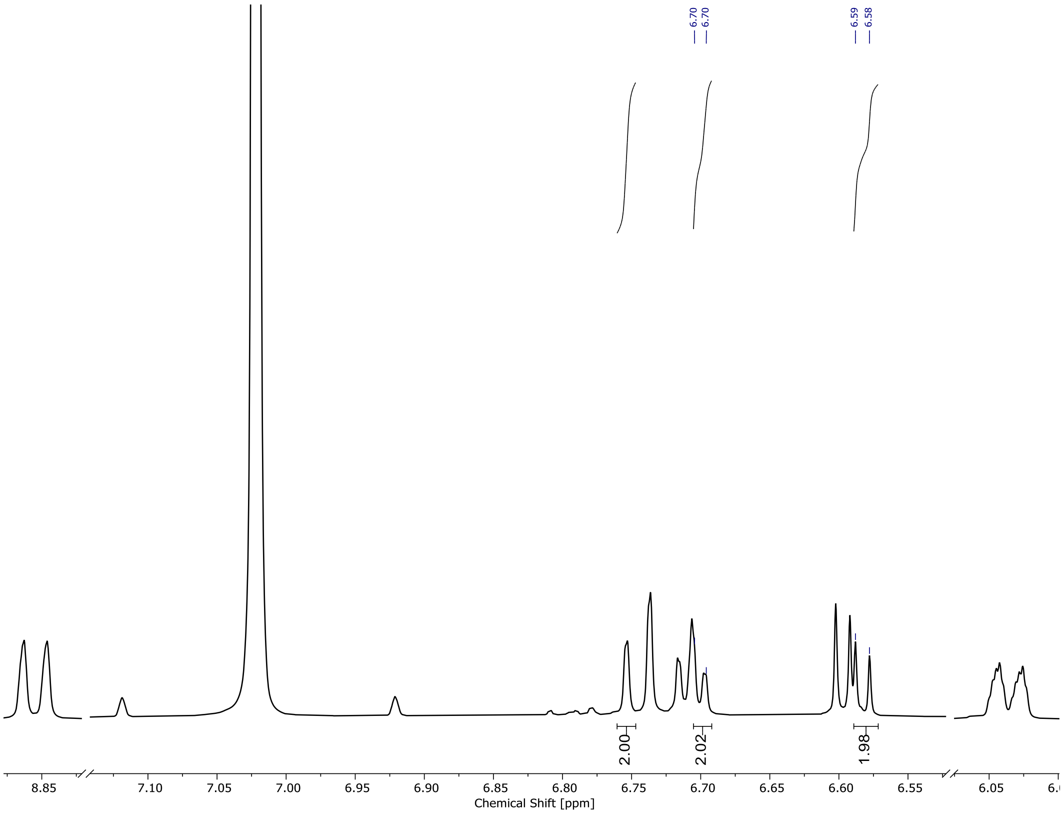

Figure S4. ^{1}H NMR spectrum (600 MHz) of a sample of **DBTTF-(ViRu)$_2$** enriched in the *E* isomer in C_6D_6.

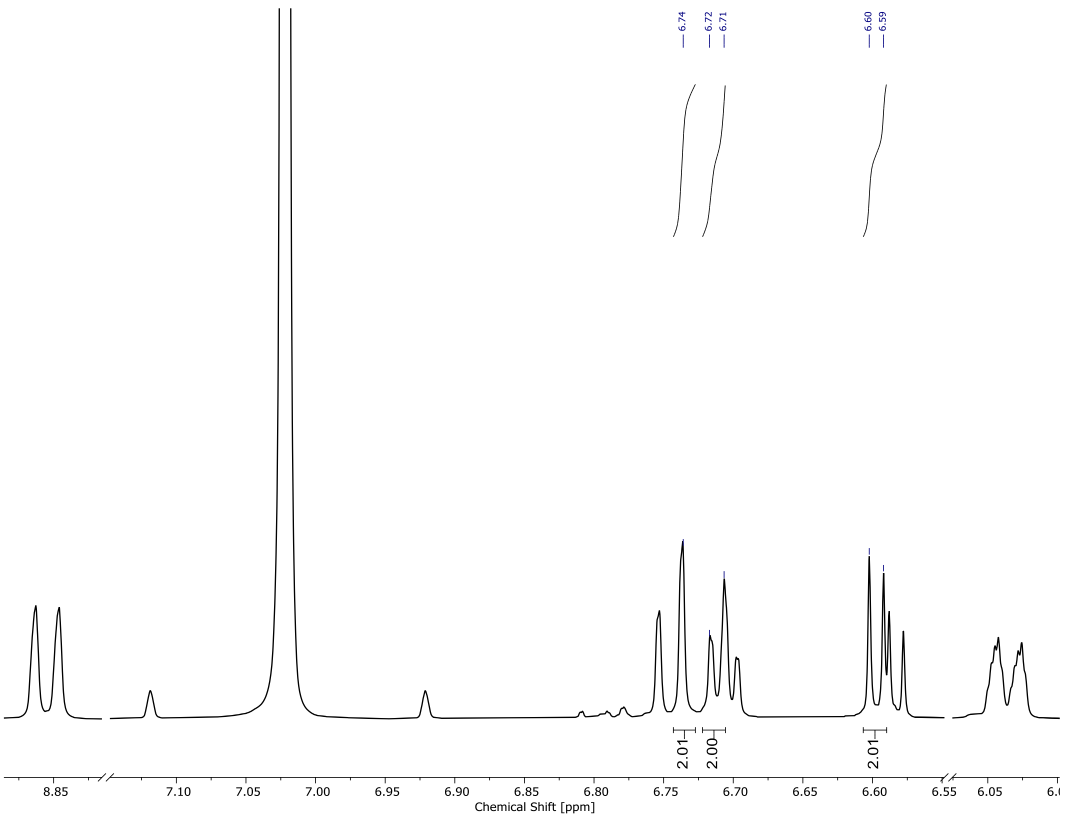

Figure S5. ^{1}H NMR spectrum (600 MHz) of a sample of **DBTTF-(ViRu)$_2$** enriched in the *Z* isomer in C_6D_6.

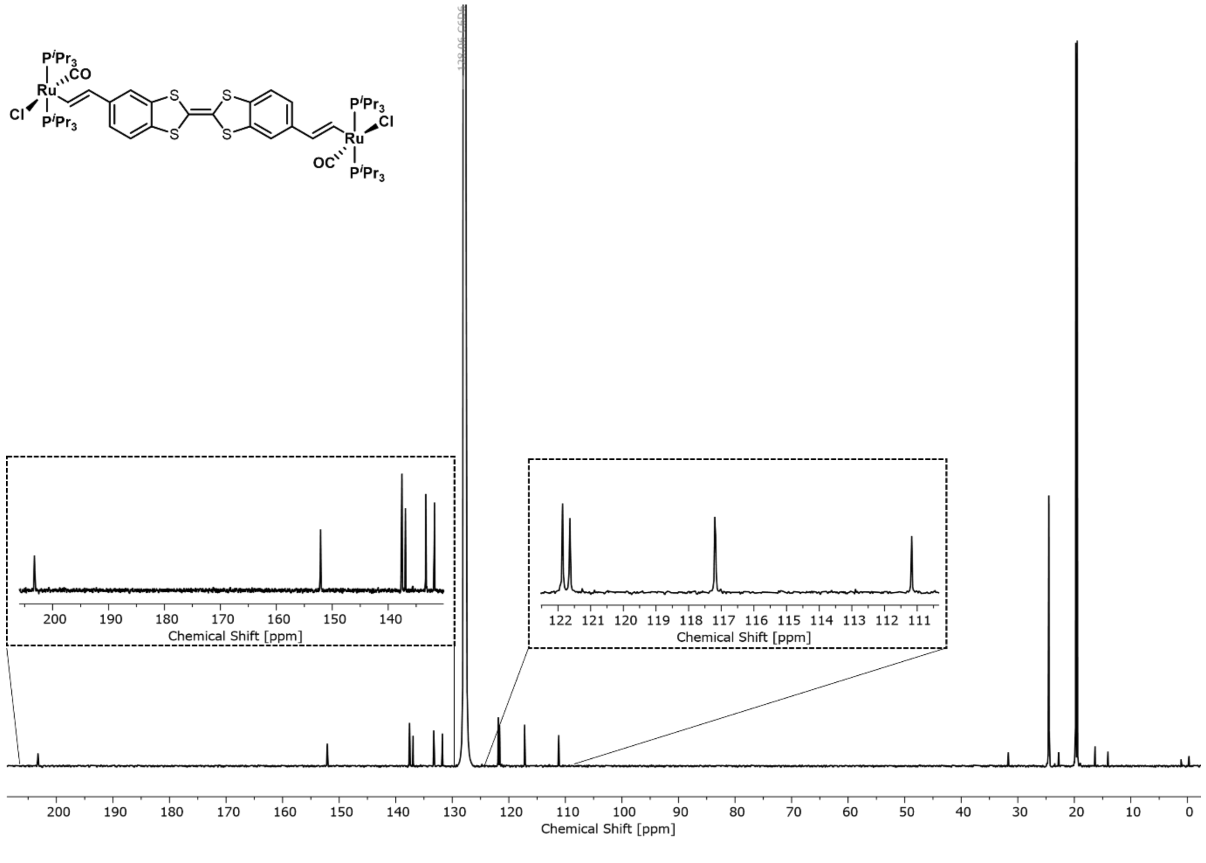

Figure S6. $^{13}C\{^{1}H\}$ NMR spectrum (800 MHz) of an *E*/*Z* isomeric mixture of **DBTTF-(ViRu)$_2$** in C_6D_6.

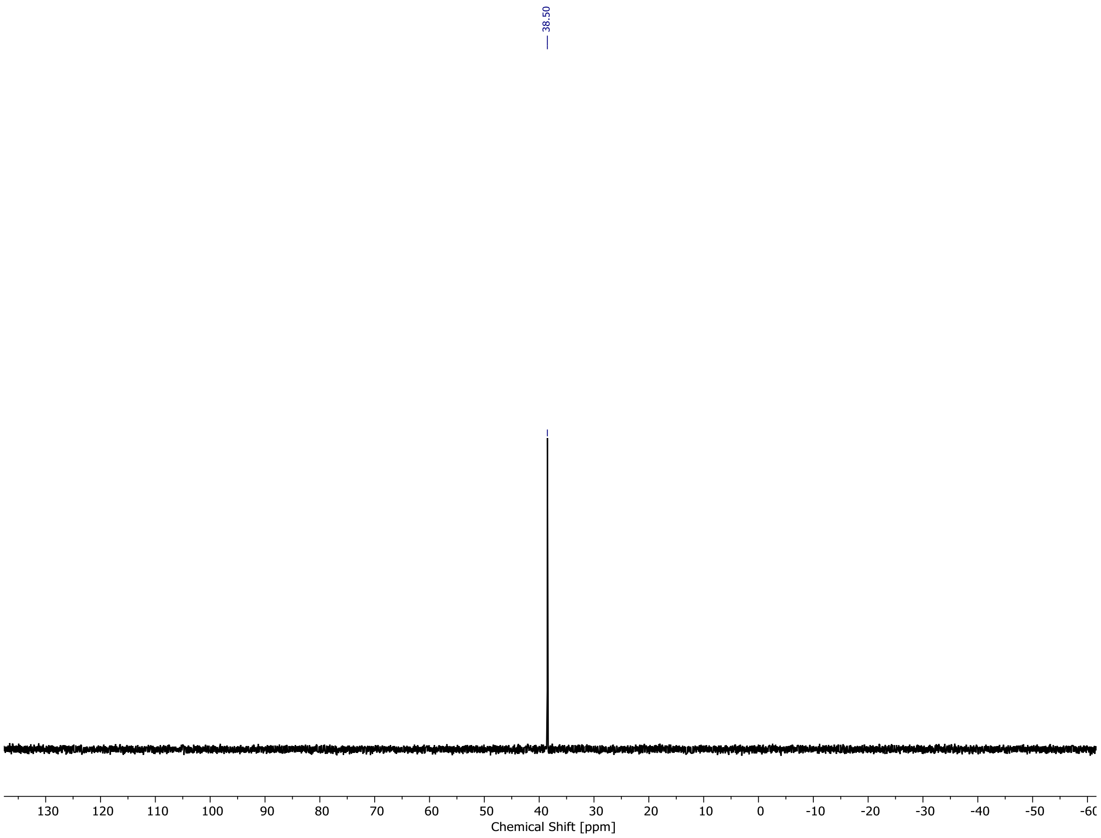

Figure S7. $^{31}P\{^{1}H\}$ NMR spectrum of an *E*/*Z* isomeric mixture of **DBTTF-(ViRu)$_2$** in C_6D_6.

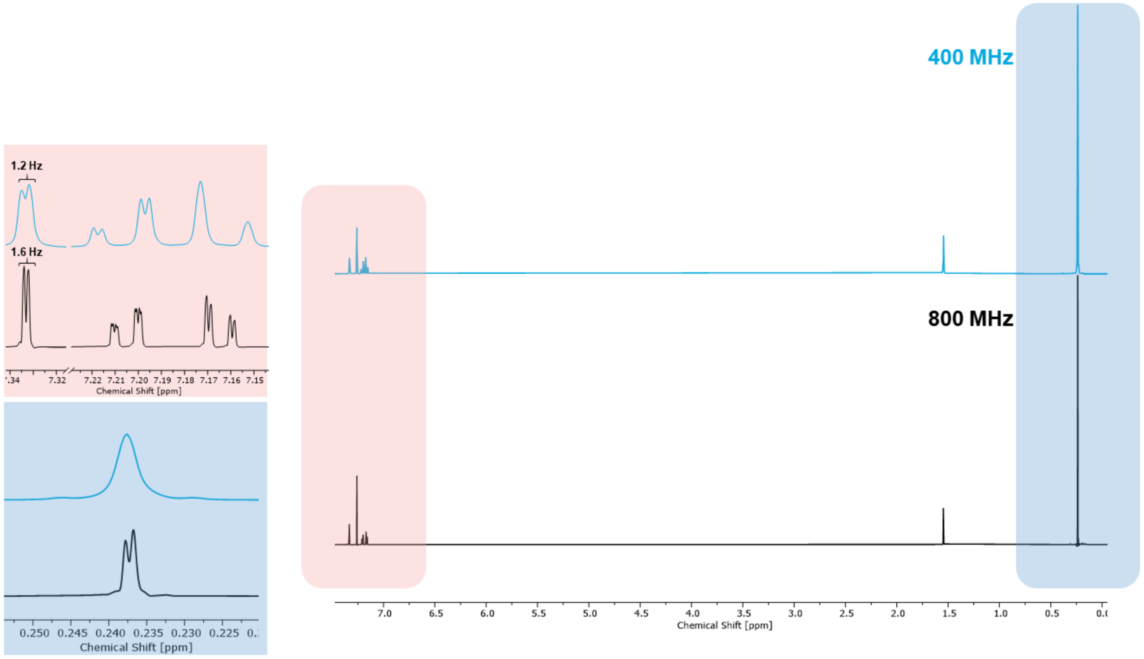

Figure S8. Top: 400 MHz ^{1}H NMR spectra of **DBTTF-(ATMS)$_2$** in $CDCl_3$; bottom: 800 MHz ^{1}H NMR spectra of **DBTTF-(ATMS)$_2$** in $CDCl_3$.

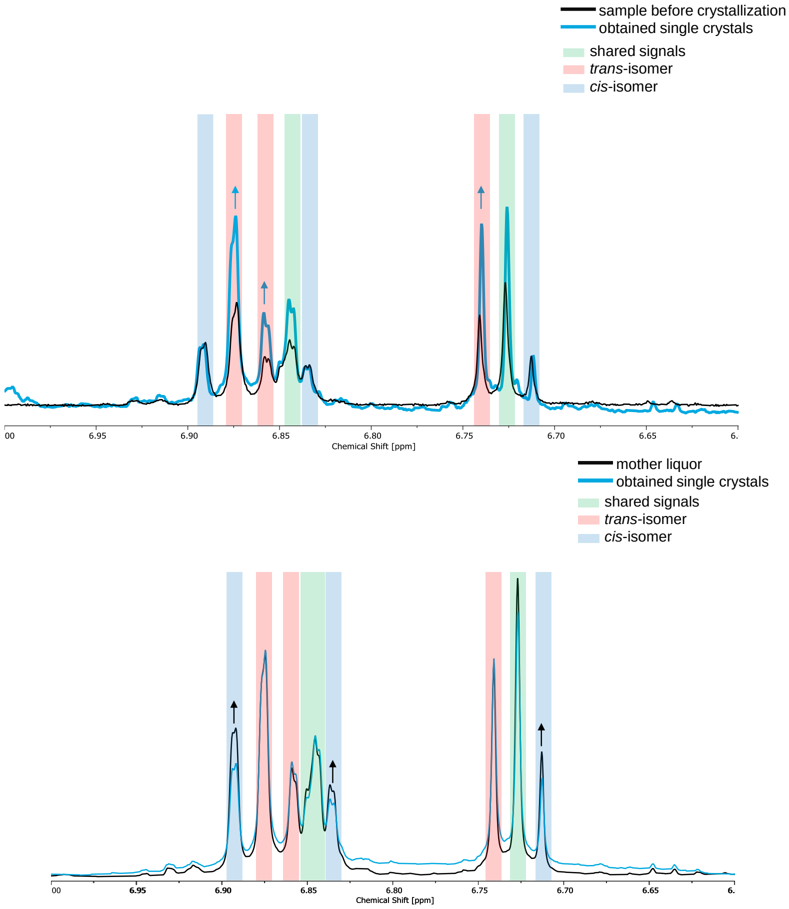

Figure S9. Top: Normalized ^{1}H NMR spectra of **DBTTF-(ViRu)₂** before crystallization (black) and of the crystallized material (blue) in C_6D_6. Bottom: normalized ^{1}H NMR spectra of crystallized **DBTTF-(ViRu)₂** (blue) and of the supernatant mother liquor (black) in C_6D_6

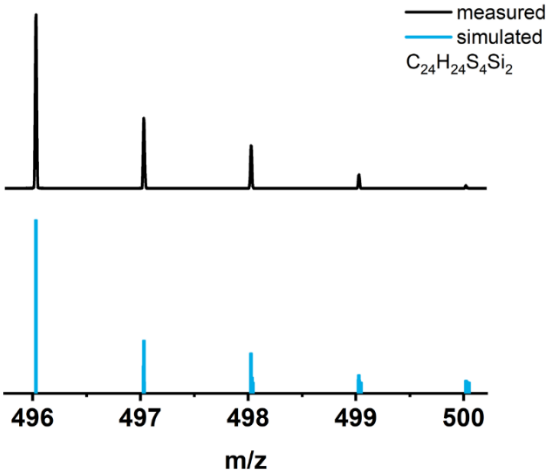

Figure S10. Experimental (black) and simulated (blue) mass spectrum of **DBTTF-(ATMS)₂**, measured in CH_2Cl_2.

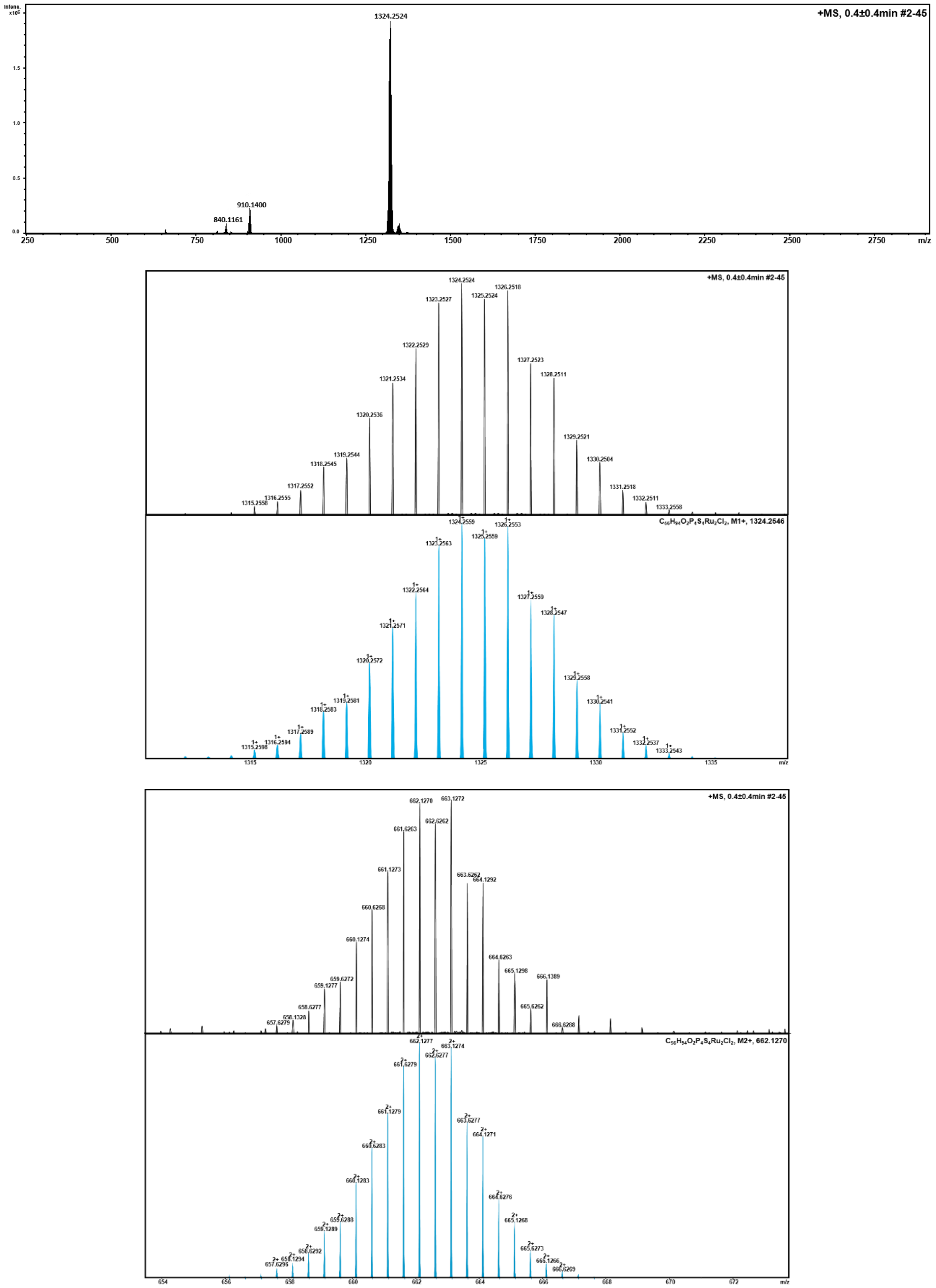

Figure S11. Top: Full mass spectrum of **DBTTF-(ViRu)$_2$**, measured in CH_2Cl_2; middle: measured (black) and simulated (blue) mass peak of the **DBTTF-(ViRu)$_2$$^+$** ion; bottom: measured (black) and simulated (blue) mass peak of the **DBTTF-(ViRu)$_2$$^{2+}$** ion.

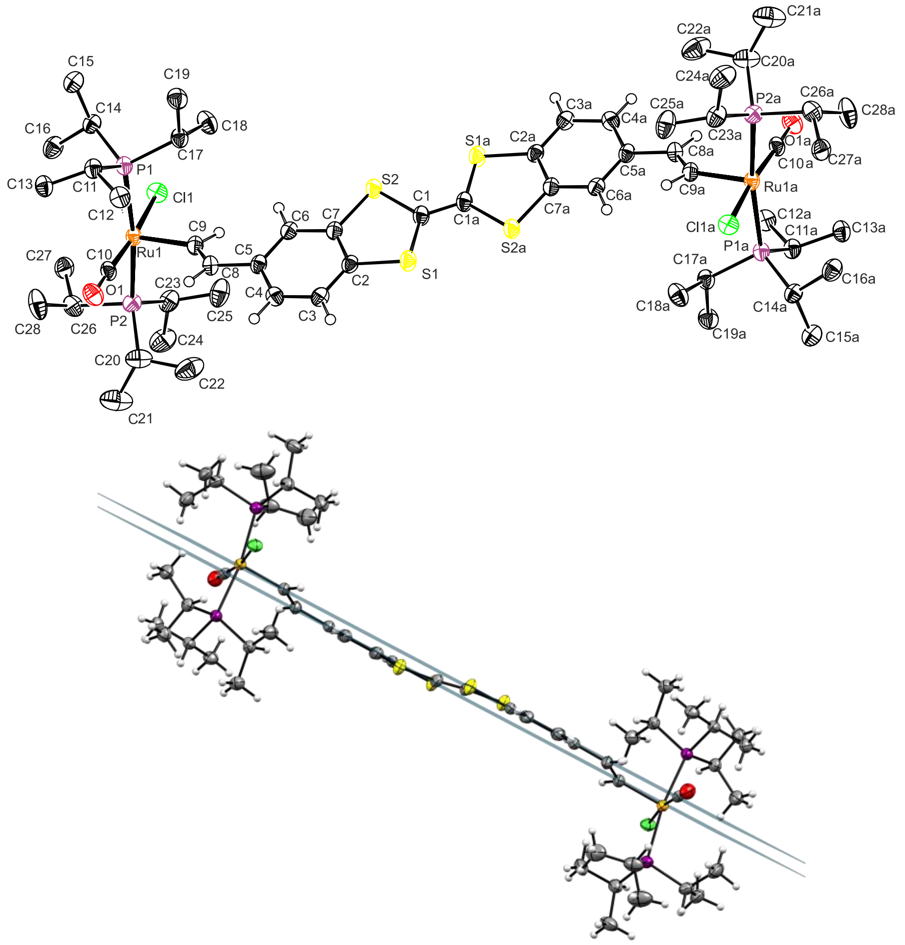

Figure S12. Molecular structure of **DBTTF-(ViRu)$_{2E}$** as determined by X-ray diffraction with atom numbering. Hydrogen atoms of the phosphane ligands are omitted for clarity reasons, ellipsoids display at the 50% probability level.

Table S1. Crystal data and structure refinement for **DBTTF-(ViRu)$_{2E}$** crystallized from CH_2Cl_2/*n*-pentane.

Empirical formular	$C_{118}Cl_4P_8Ru_4S_8H_{0.5}$
Formula weight	2532.00
Temperature [K]	100
Crystal system	monoclinic
Space group	$P2_1/n$
a [Å] 12.5098(4)	
b [Å]	15.4641(3)
c [Å]	18.2794(6)
α [°]	90
β [°]	106.014(3)
γ [°]	90
Volume [Å^3]	3398.98(18)
Z	2
ρ_{calc} [g/cm^3]	2.474
μ/mm^{-1}	1.547
F(000)	2465.0
Crystal size [mm^3]	0.2 × 0.133 × 0.1
Radiation	MoKα (λ = 0.71073)
2Θ range for data collection [°]	4.29 to 55.174
Index ranges	$-16 \le h \le 16$, $-20 \le k \le 20$, $-23 \le l \le 20$
Reflections collected	21578
Independent reflections	7805 [R_{int} = 0.0237, R_{sigma} = 0.0260]
Data/restraints/parameters	7805/0/329
Goodness-of-fit on F^2	1.041
Final *R* indexes [$I \ge 2\sigma$ (I)]	R_1 = 0.0364, wR_2 = 0.0818
Final *R* indexes [all data]	R_1 = 0.0529, wR_2 = 0.0931
Largest diff. peak/hole [e Å^{-3}]	0.57/-0.81

Table S2. Bond lengths [Å] for **DBTTF-(ViRu)$_{2E}$**.

Atom	Atom	Length/Å	Atom	Atom	Length/Å
Ru1	Cl1	2.4161(8)	C3	C4	1.388(4)
Ru1	P1	2.4090(8)	C4	C5	1.396(4)
Ru1	P2	2.4023(8)	C5	C6	1.397(4)
Ru1	C9	1.989(3)	C5	C8	1.478(4)
Ru1	C10	1.814(3)	C6	C7	1.391(4)
S1	C1	1.748(3)	C8	C9	1.337(4)
S1	C2	1.760(3)	C11	C12	1.523(4)
S2	C1	1.761(3)	C11	C13	1.533(4)
S2	C7	1.753(3)	C14	C15	1.537(4)
P1	C11	1.857(3)	C14	C16	1.539(4)
P1	C14	1.858(3)	C17	C18	1.530(4)
P1	C17	1.866(3)	C17	C19	1.532(4)
P2	C20	1.862(4)	C20	C21	1.528(5)
P2	C23	1.848(3)	C20	C22	1.528(6)
P2	C26	1.857(3)	C23	C24	1.534(5)
O1	C10	1.157(4)	C23	C25	1.528(5)
C1	C1a	1.349(6)	C26	C27	1.528(5)
C2	C3	1.383(4)	C26	C28	1.528(5
C2	C7	1.399(4)			

Table S3. Bond angles [°] for **DBTTF-(ViRu)$_{2E}$**.

**Atom**	**Atom**	**Atom**	**Angle/˚**	**Atom**	**Atom**	**Atom**	**Angle/˚**
P1	Ru1	Cl1	88.28(3)	C2	C3	C4	119.2(3)
P2	Ru1	Cl1	88.49(3)	C3	C4	C5	122.2(3)
P2	Ru1	P1	170.51(3)	C4	C5	C6	118.0(3)
C9	Ru1	Cl1	98.23(9)	C4	C5	C8	120.7(3)
C9	Ru1	P1	90.72(8)	C6	C5	C8	121.3(3)
C9	Ru1	P2	98.57(8)	C7	C6	C5	120.3(3)
C10	Ru1	Cl1	171.76(9)	C2	C7	S2	116.9(2)
C10	Ru1	P1	94.27(9)	C6	C7	S2	122.6(2)
C10	Ru1	P2	87.75(9)	C6	C7	C2	120.5(3)
C10	Ru1	C9	89.58(13)	C9	C8	C5	124.8(3)
C1	S1	C2	95.06(14)	C8	C9	Ru1	134.8(2)
C7	S2	C1	94.63(14)	O1	C10	Ru1	177.3(3)
C11	P1	Ru1	116.67(10)	C12	C11	P1	110.7(2)
C11	P1	C14	105.55(14)	C12	C11	C13	111.0(3)
C11	P1	C17	102.87(13)	C13	C11	P1	112.8(2)
C14	P1	Ru1	110.46(10)	C15	C14	P1	115.7(2)
C14	P1	C17	106.82(14)	C15	C14	C16	110.5(3)
C17	P1	Ru1	113.64(10)	C16	C14	P1	111.3(2)
C20	P2	Ru1	115.83(12)	C18	C17	P1	112.9(2)
C23	P2	Ru1	115.75(11)	C18	C17	C19	108.9(3)
C23	P2	C20	110.31(17)	C19	C17	P1	117.1(2)
C23	P2	C26	103.56(15)	C21	C20	P2	116.9(3)
C26	P2	Ru1	105.85(11)	C21	C20	C22	110.4(3)
C26	P2	C20	103.93(17)	C22	C20	P2	111.6(3)
S1	C1	S2	115.79(16)	C24	C23	P2	115.7(3)
C1[1]	C1	S1	122.8(3)	C25	C23	P2	113.9(2)
C1[1]	C1	S2	121.4(3)	C25	C23	C24	111.4(3)
C3	C2	S1	123.9(2)	C27	C26	P2	112.2(2)
C3	C2	C7	119.8(3)	C27	C26	C28	110.5(3)
C7	C2	S1	116.3(2)	C28	C26	P2	110.2(2)

Table S4. Torsion angles [°] for **DBTTF-(ViRu)$_{2E}$**.

A	B	C	D	Angle/°	A	B	C	D	Angle/°
Ru1	P1	C11	C12	64.3(2)	C5	C8	C9	Ru1	-174.3(2)
Ru1	P1	C11	C13	-60.7(2)	C6	C5	C8	C9	20.7(4)
Ru1	P1	C14	C15	166.0(2)	C7	S2	C1	S1	-11.64(19)
Ru1	P1	C14	C16	38.8(2)	C7	S2	C1	$C1^{1}$	168.6(4)
Ru1	P1	C17	C18	65.0(3)	C7	C2	C3	C4	0.9(4)
Ru1	P1	C17	C19	-167.2(2)	C8	C5	C6	C7	-179.0(3)
Ru1	P2	C20	C21	151.2(3)	C11	P1	C14	C15	39.1(3)
Ru1	P2	C20	C22	-80.4(3)	C11	P1	C14	C16	-88.2(2)
Ru1	P2	C23	C24	-171.4(2)	C11	P1	C17	C18	-167.9(2)
Ru1	P2	C23	C25	57.7(3)	C11	P1	C17	C19	-40.2(3)
Ru1	P2	C26	C27	69.7(2)	C14	P1	C11	C12	-172.6(2)
Ru1	P2	C26	C28	-53.8(3)	C14	P1	C11	C13	62.3(3)
S1	C2	C3	C4	-179.9(2)	C14	P1	C17	C18	-57.0(3)
S1	C2	C7	S2	-2.5(3)	C14	P1	C17	C19	70.7(3)
S1	C2	C7	C6	179.3(2)	C17	P1	C11	C12	-60.8(2)
C1	S1	C2	C3	175.9(3)	C17	P1	C11	C13	174.2(2)
C1	S1	C2	C7	-4.9(2)	C17	P1	C14	C15	-69.9(3)
C1	S2	C7	C2	8.3(2)	C17	P1	C14	C16	162.8(2)
C1	S2	C7	C6	-173.5(2)	C20	P2	C23	C24	54.6(3)
C2	S1	C1	S2	10.62(19)	C20	P2	C23	C25	-76.3(3)
C2	S1	C1	$C1^{1}$	-169.6(4)	C20	P2	C26	C27	-167.8(2)
C2	C3	C4	C5	1.1(4)	C20	P2	C26	C28	68.6(3)
C3	C2	C7	S2	176.8(2)	C23	P2	C20	C21	-74.9(4)
C3	C2	C7	C6	-1.4(4)	C23	P2	C20	C22	53.5(3)
C3	C4	C5	C6	-2.5(4)	C23	P2	C26	C27	-52.5(3)
C3	C4	C5	C8	178.5(3)	C23	P2	C26	C28	-176.0(3)
C4	C5	C6	C7	1.9(4)	C26	P2	C20	C21	35.5(4)
C4	C5	C8	C9	-160.3(3)	C26	P2	C20	C22	164.0(3)
C5	C6	C7	S2	-178.1(2)	C26	P2	C23	C24	-56.0(3)
C5	C6	C7	C2	0.0(4)	C26	P2	C23	C25	173.0(3)

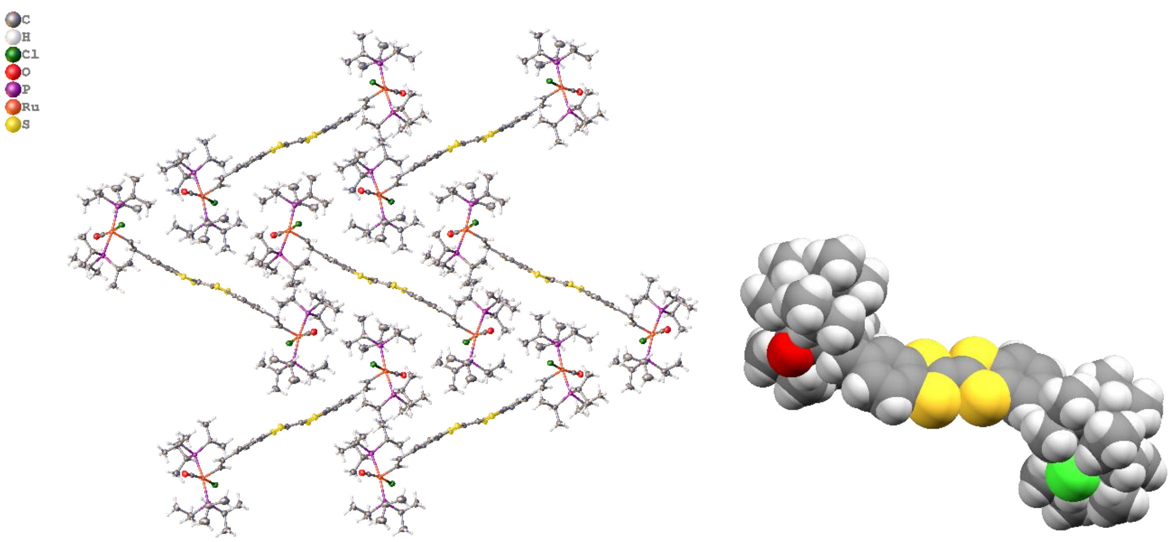

Figure S13. Left: Packing of individual molecules of **DBTTF-(ViRu)$_{2E}$** in the solid state; right: Space-filling representation of the a molecule of **DBTTF-(ViRu)$_{2E}$** in the crystal.

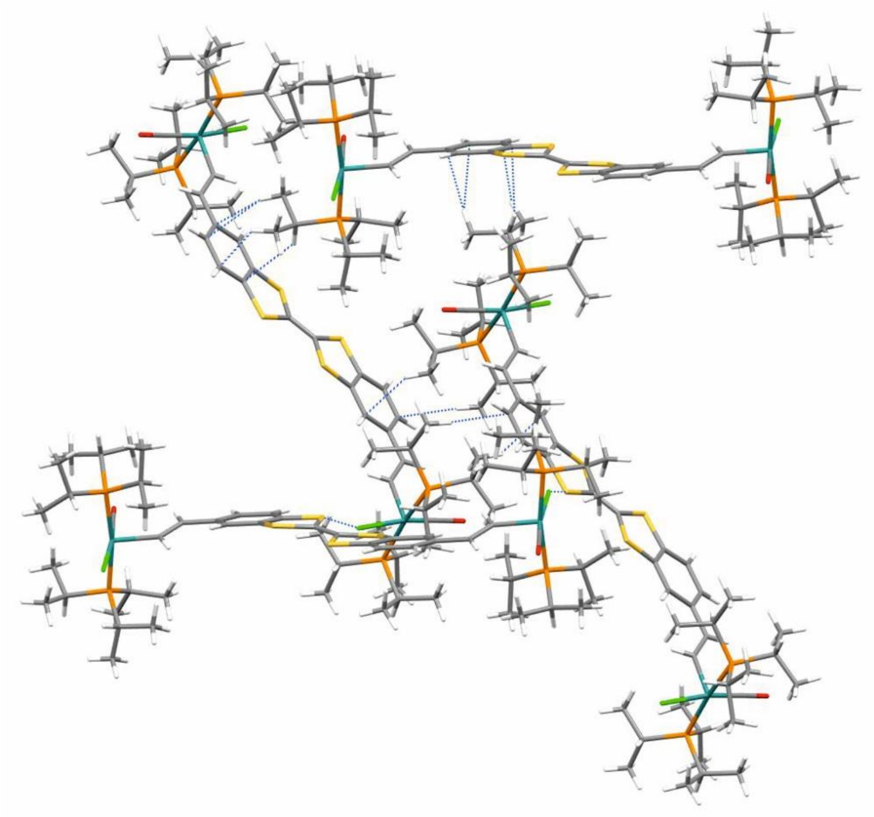

Figure S14. Intermolecular interactions between individual molecules of **DBTTF-(ViRu)$_{2E}$** in the crystalline state. S···Cl and CH··· π contacts shorter than the sum of the VdW radii are indicated by dark blue broken lines.

Electrochemistry

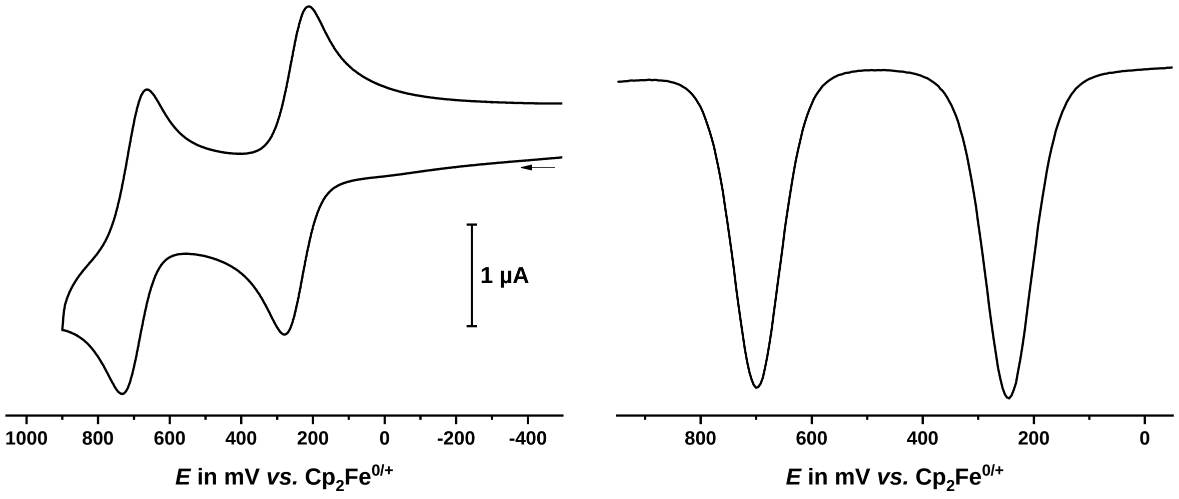

Figure S15. Cyclic (left) and square wave (right) voltammograms of **DBTTF-(ATMS)₂** (0.1 M NBu_4^+ PF_6^-/CH_2Cl_2 at v = 100 mV/s, r.t.).

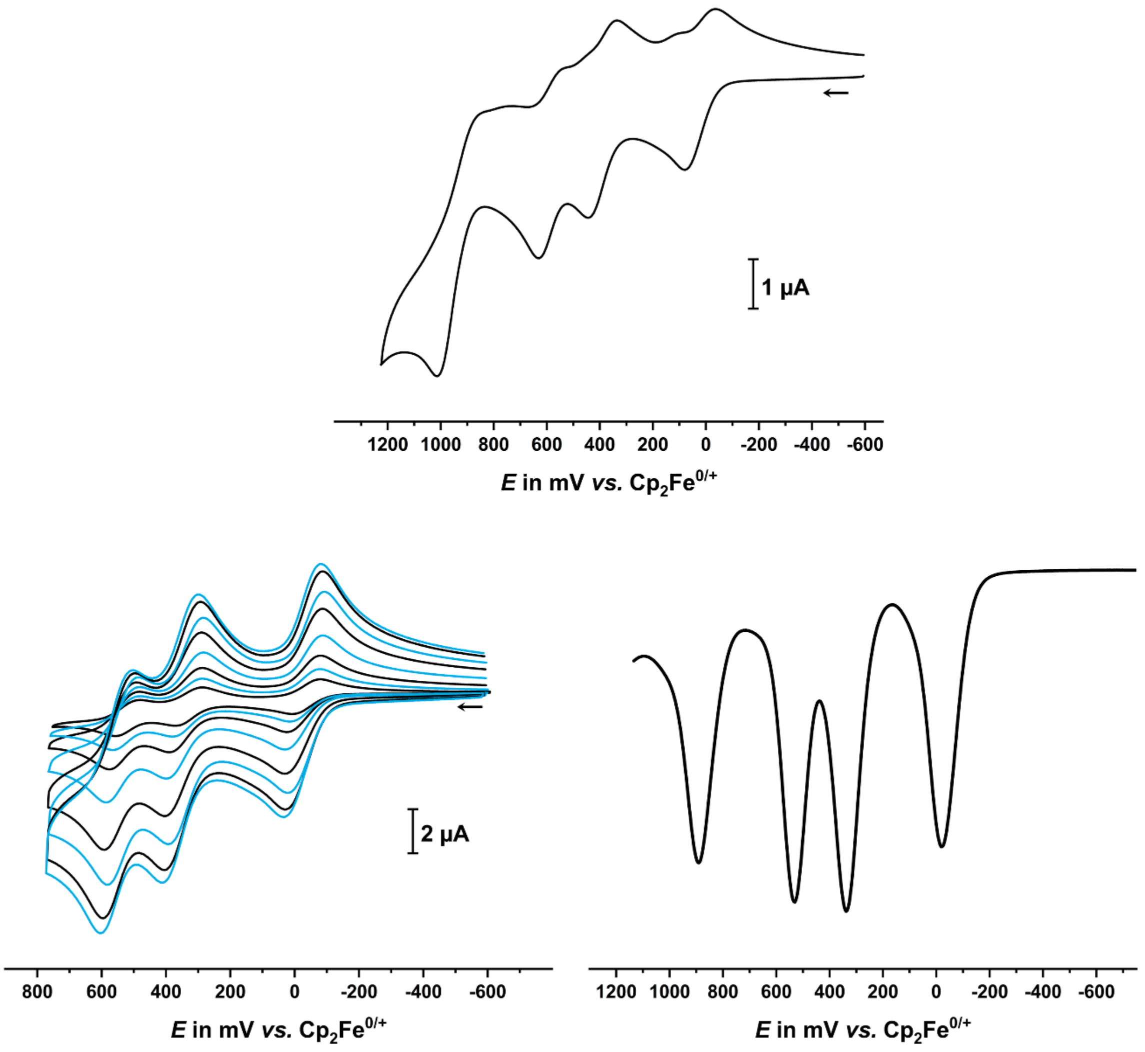

Figure S16. Top and bottom right: Cyclic and square wave voltammogram of all four oxidations of **DBTTF-(ViRu)₂** (0.1 M NBu_4^+ PF_6^- / CH_2Cl_2, v = 100 mV/s, r.t.); bottom left: cyclic voltammograms of the first three oxidations of **DBTTF-(ViRu)₂** at v = 25, 50, 100, 200, 400, 600, 800, 1000 mV/s.

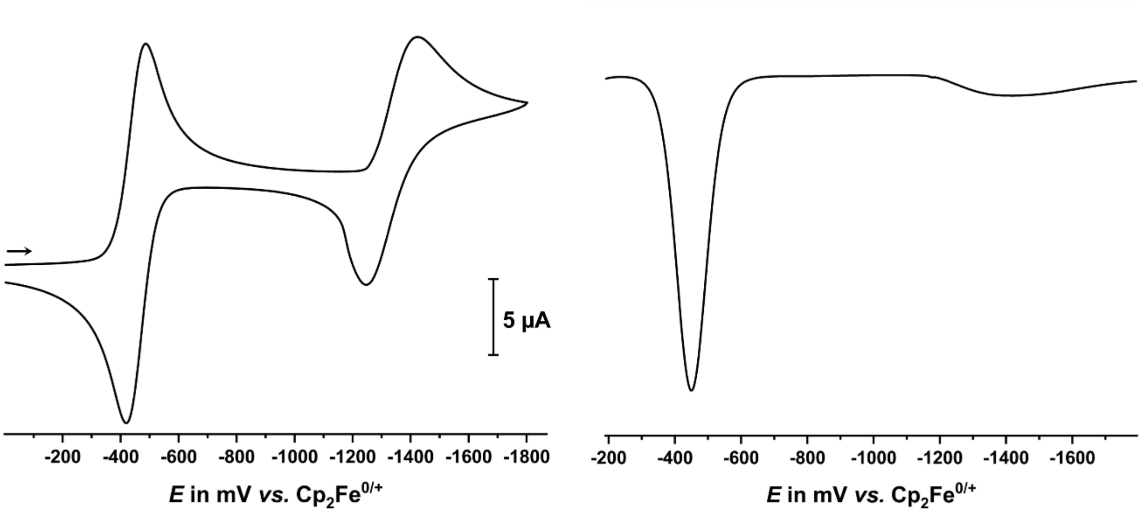

Figure S17. Cyclic (left) and square wave voltammogram (right) (0.1 M NBu_4^+ PF_6^- / CH_2Cl_2, *v* = 100 mV/s, r.t.) of **F_4BQ**.

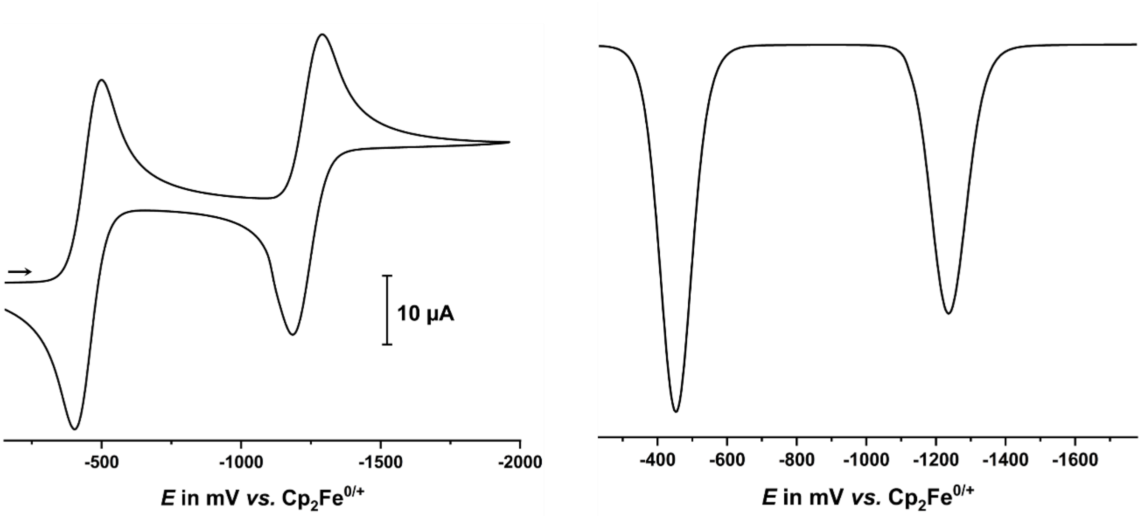

Figure S18. Cyclic (left) and square wave voltammogram (right) (0.1 M NBu_4^+ PF_6^- / CH_2Cl_2, *v* = 100 mV/s, r.t.) of **Cl_4BQ**.

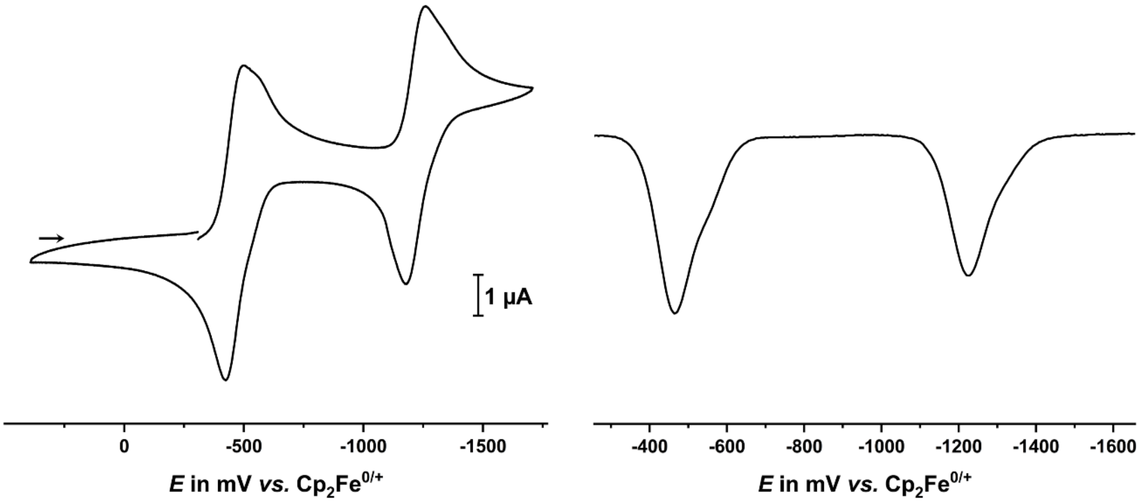

Figure S19. Cyclic (left) and square wave voltammogram (right) (0.1 M NBu_4^+ PF_6^- / CH_2Cl_2, *v* = 100 mV/s, r.t.) of **Br_4BQ**.

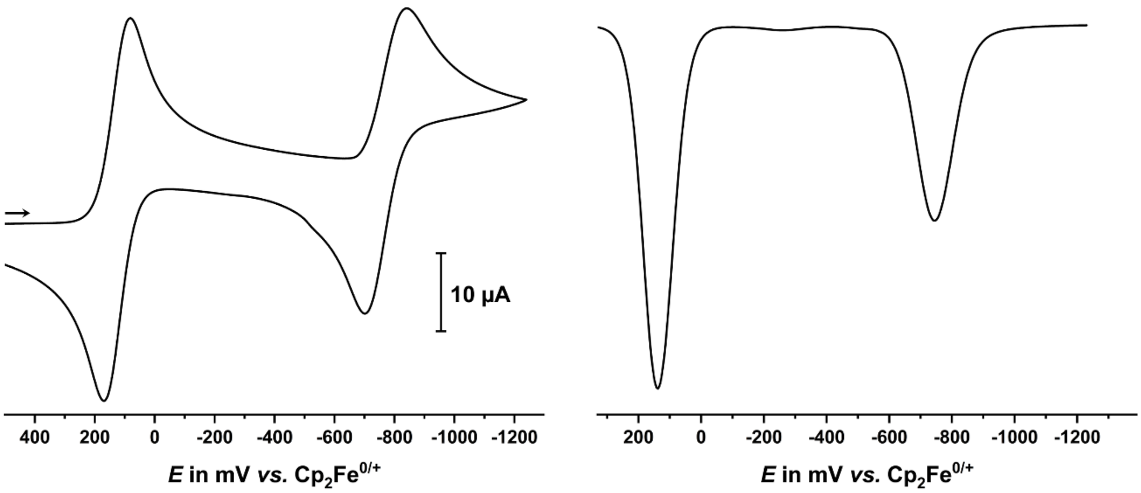

Figure S20. Cyclic (left) and square wave voltammogram (right) (0.1 M $NBu_4^+ PF_6^-$ / CH_2Cl_2, *v* = 100 mV/s, r.t.) of **DDQ**.

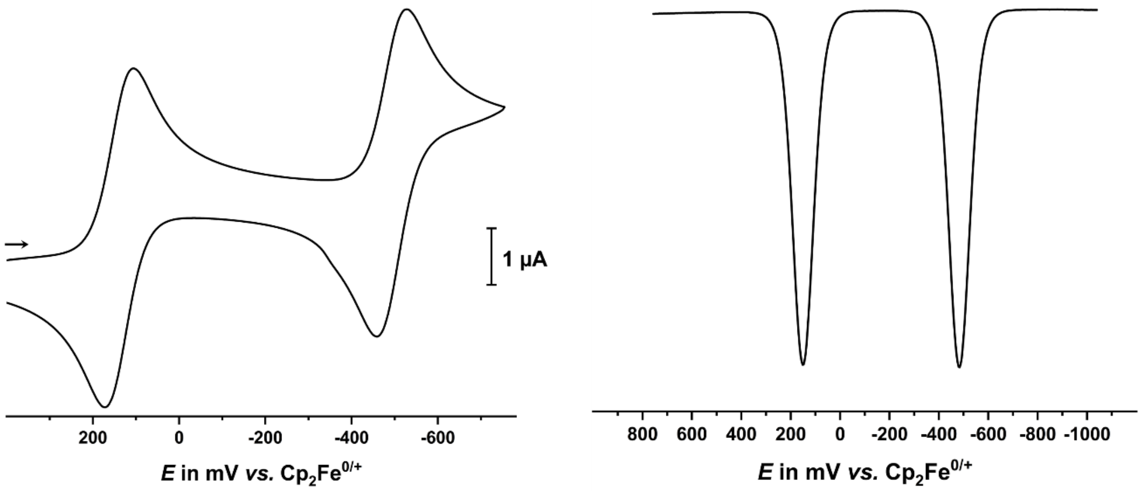

Figure S21. Cyclic (left) and square wave voltammogram (right) (0.1 M $NBu_4^+ PF_6^-$ / CH_2Cl_2, *v* = 100 mV/s, r.t.) of **F_4TCNQ**.

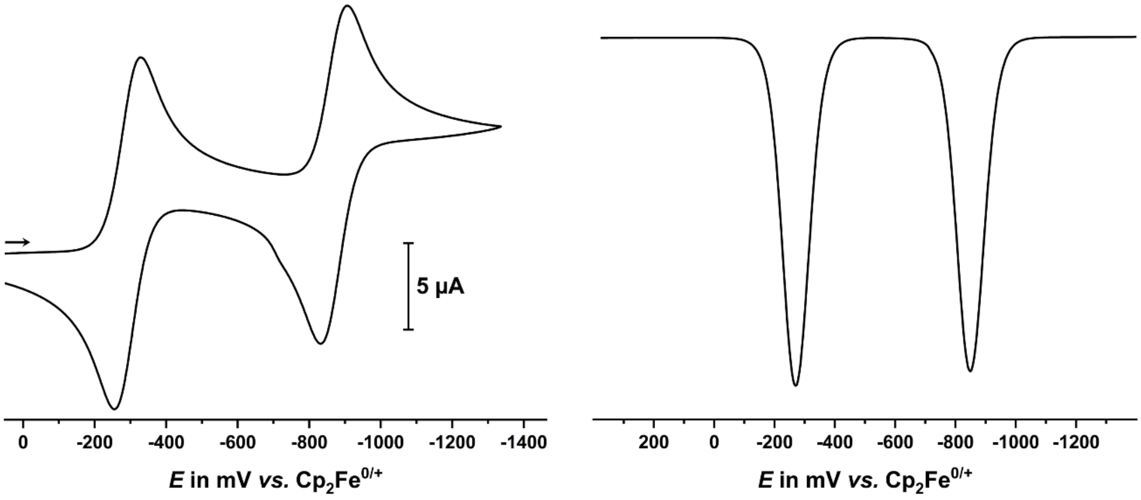

Figure S22. Cyclic (left) and square wave voltammogram (right) (0.1 M $NBu_4^+ PF_6^-$ / CH_2Cl_2, *v* = 100 mV/s, r.t.) of **TCNQ**.

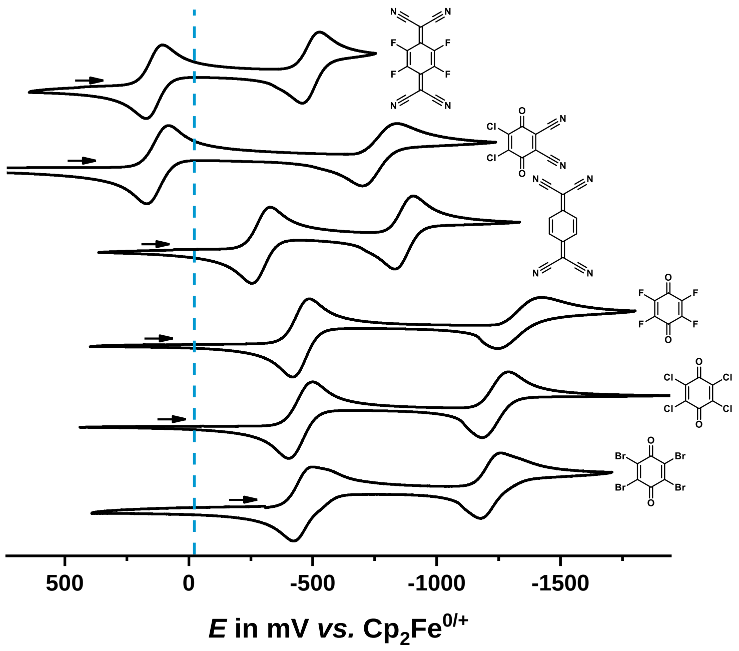

Figure S23. Comparison of the cyclic voltammograms of all acceptors of this study in 0.1 M $NBu_4^+ PF_6^-$ / CH_2Cl_2 at *v* = 100 mV/s, Blue dotted lines at -5 mV mark the half-wave potential for the first oxidation of **DBTTF-(ViRu)$_2$**.

EPR Spectra

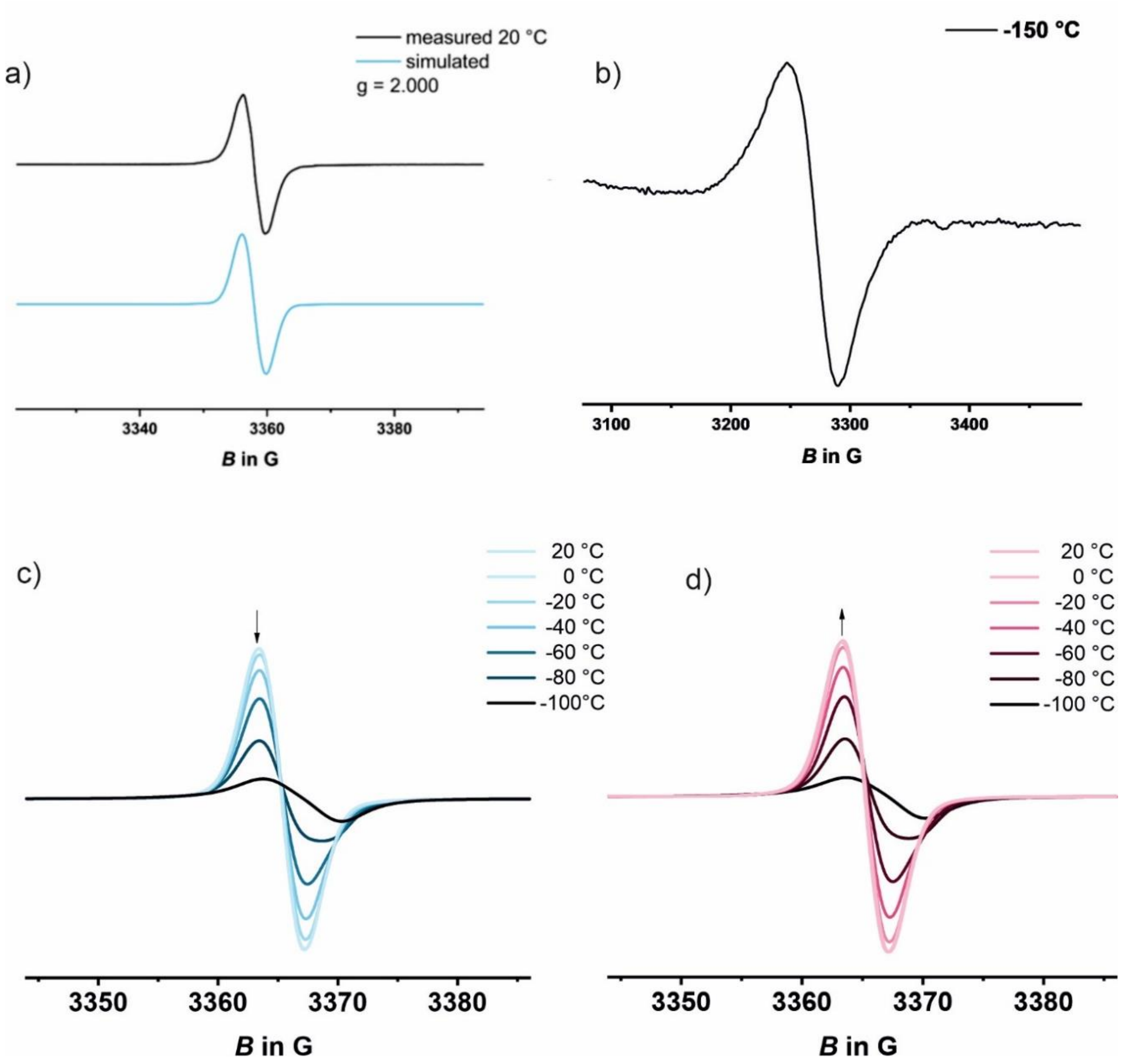

Figure S24. a) Experimental (black line) and simulated (blue line) EPR spectra of **DBTTF-(ViRu)$_2^+$** in CH_2Cl_2 at 20°C; b) EPR spectrum of **DBTTF-(ViRu)$_2^+$** in frozen CH_2Cl_2 solution at -150 °C; c) and d) Temperature-dependent EPR spectra recorded during (c)) a cooling and (d)) the corresponding warming cycle, demonstrating the reversibility of *T*-induced changes.

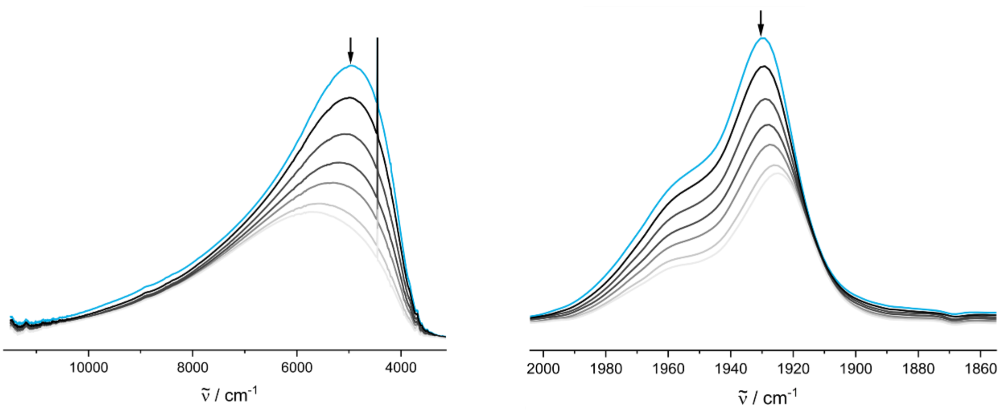

Figure S25. Time-dependent (5 min intervals between each scan) IR (right) and NIR (left) spectra of **DBTTF-(ViRu)$_2^{2+}$**, chemically prepared by oxidation of **DBTTF-(ViRu)$_2$** with 2.1 equivalents of diacetylferrocinium hexafluoroantimonate at r.t.

Electronic Spectra

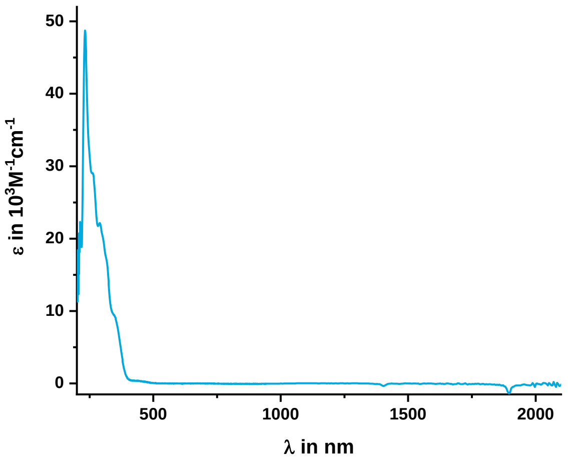

Figure S26. UV/Vis spectrum of **DBTTF-I_2** in CH_2Cl_2.

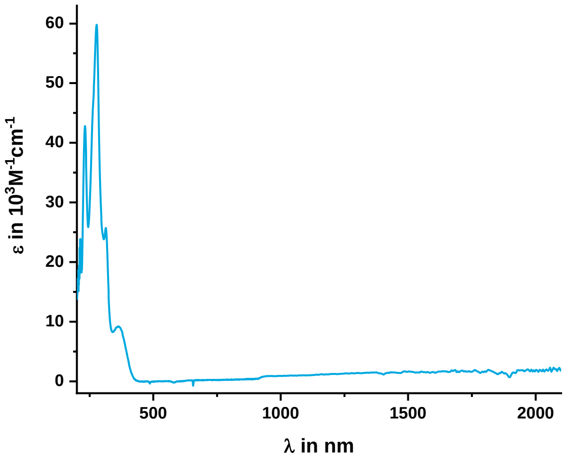

Figure S27. UV/Vis spectrum of **DBTTF-(ATMS)$_2$** in CH_2Cl_2.

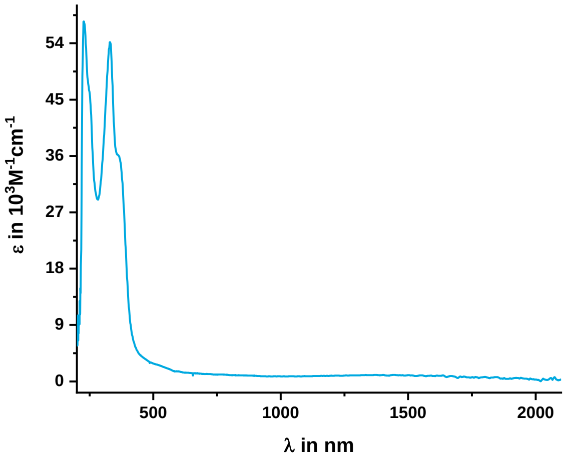

Figure S28. UV/Vis spectrum of **DBTTF-(ViRu)$_2$** in CH_2Cl_2.

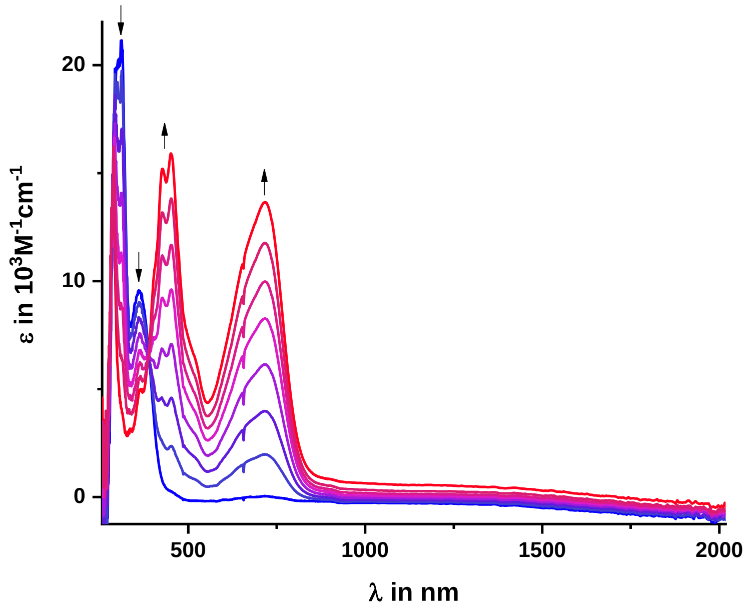

Figure S29. Changes in UV/Vis/NIR spectra upon the electrochemical oxidation of **DBTTF-(ATMS)$_2$** (1,2-$C_2H_4Cl_2$ / $^nBu_4NPF_6$ (0.2 M), r.t.).

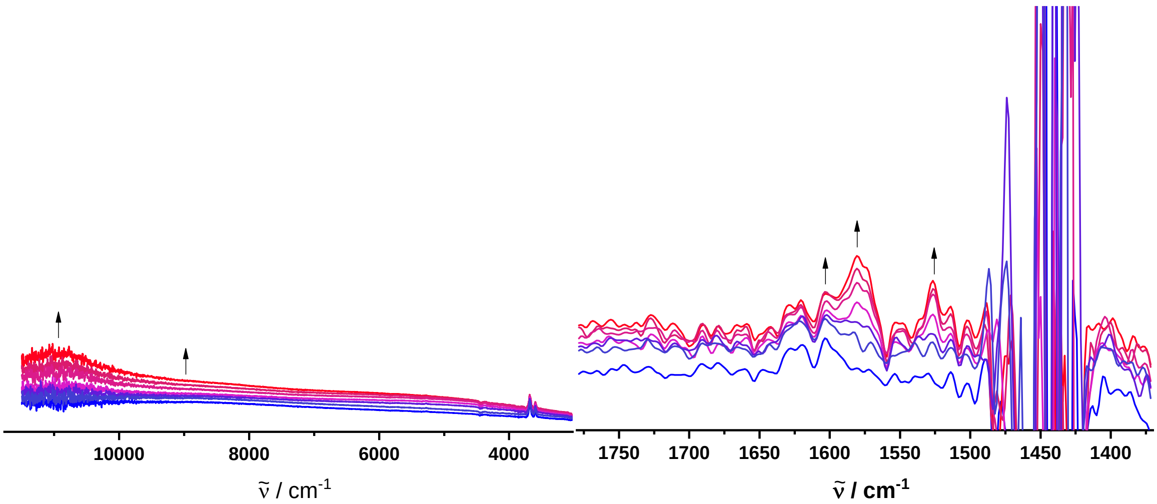

Figure S30. Changes in NIR (left) and IR (right) spectra upon the electrochemical oxidation of **DBTTF-(ATMS)$_2$** (1,2-$C_2H_4Cl_2$ / $^nBu_4NPF_6$ (0.2 M), r.t.).

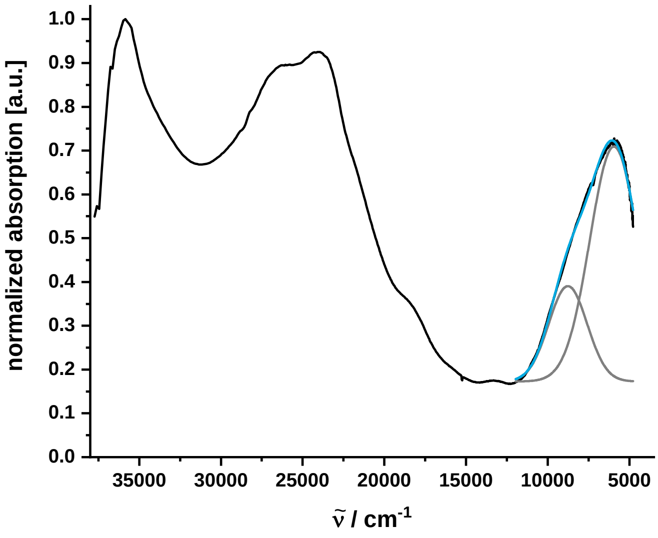

Figure S31. UV/Vis spectrum of **DBTTF-(ViRu)$_2^{2+}$** with deconvolution of the CT band in CH_2Cl_2.

Quantum Chemistry

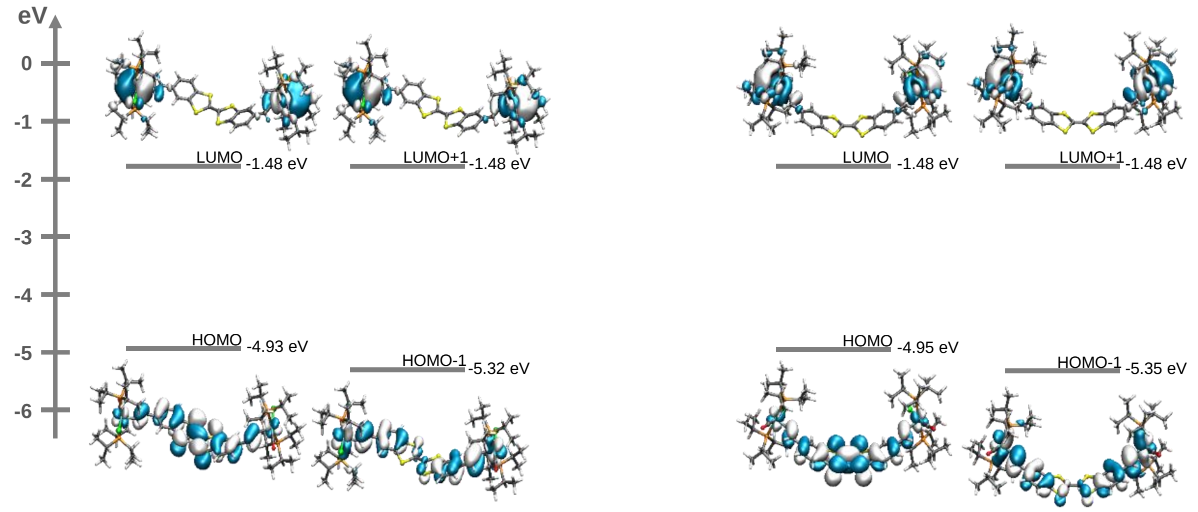

Figure S32. Computed (pbe1pbe) frontier orbitals of **DBTTF-(ViRu)$_{2E}$** (left) and **DBTTF-(ViRu)$_{2Z}$** (right).

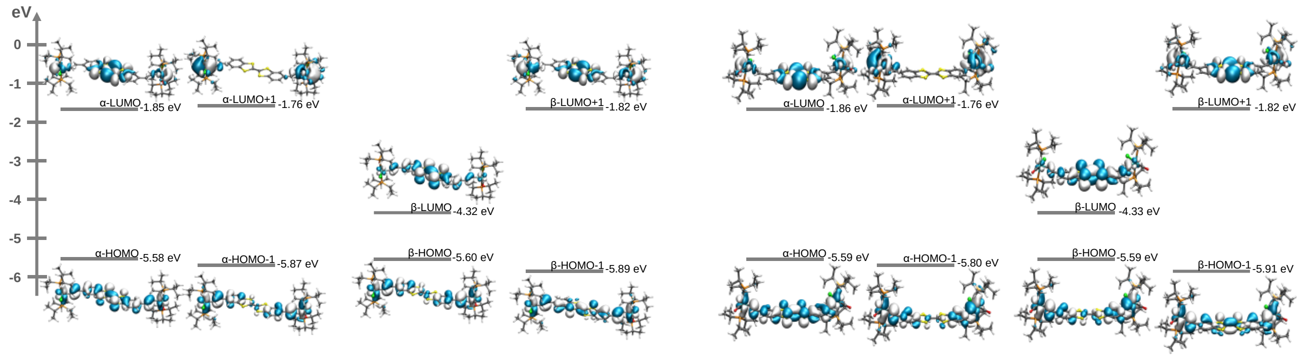

Figure S33. Computed (pbe1pbe) frontier orbitals of **DBTTF-(ViRu)$_{2E}^{+}$** (left) and **DBTTF-(ViRu)$_{2Z}^{+}$** (right).

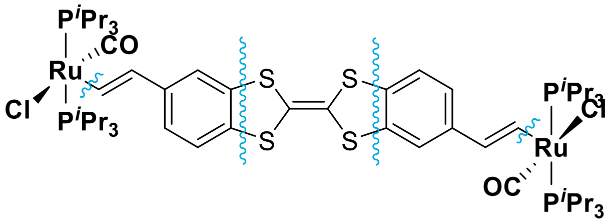

Table S5. Composition and energies of the calculated (pbe1pbe) occupied frontier MOs of **DBTTF-(ViRu)$_{2E}$**.

MO	eV	{Ru$_1$}	VinylBenz$_1$	TTF core	VinylBenz$_2$	{Ru$_2$}
HOMO	-4.93	5	18	54	18	5
HOMO-1	-5.35	18	30	5	30	18
HOMO-2	-5.65	16	19	30	19	16

Table S 6. Composition and energies of the calculated (M062X) occupied frontier MOs of **DBTTF-(ViRu)$_{2E}$**.

MO	eV	{Ru_1}	$VinylBenz_1$	TTF core	$VinylBenz_2$	{Ru_2}
HOMO	-5.64	2	14	68	14	2
HOMO-1	-6.41	14	32	8	32	14
HOMO-2	-6.69	16	25	20	24	15

Table S7. Composition and energies of the calculated (pbe1pbe) occupied frontier MOs of **DBTTF-(ViRu)$_{2Z}$**.

MO	eV	{Ru_1}	$VinylBenz_1$	TTF core	$VinylBenz_2$	{Ru_2}
HOMO	-4.95	5	19	52	18	5
HOMO-1	-5.32	17	30	6	30	17
HOMO-2	-5.68	16	19	31	19	16

Table S8. Composition and energies of selected calculated (pbe1pbe) MOs of **DBTTF-(ViRu)$_{2E}^{+}$**.

MO	eV	{Ru_1}	$VinylBenz_1$	TTF core	$VinylBenz_2$	{Ru_2}
α-LUMO	-1.85	13	9	55	9	13
β-LUMO	-4.32	3	14	66	14	3
α-HOMO	-5.58	12	24	29	24	12
β-HOMO	-5.60	19	29	4	29	19
α-HOMO-1	-5.81	21	28	3	28	21
β-HOMO-1	-5.89	19	22	18	22	19
α-HOMO-2	-6.28	12	13	51	13	12
β-HOMO-2	-6.62	49	0	0	0	50

Table S9. Composition and energies of selected calculated (M062X) MOs of **DBTTF-(ViRu)$_{2E}^{+}$**.

MO	eV	{Ru$_1$}	VinylBenz$_1$	TTF core	VinylBenz$_2$	{Ru$_2$}
α-LUMO	-1.16	3	10	74	10	3
β-LUMO	-3.93	1	10	77	10	1
α-HOMO	-6.67	9	23	34	24	10
β-HOMO	-6.83	17	29	5	30	18
α-HOMO-1	-6.97	20	29	3	28	20
β-HOMO-1	-7.04	21	26	8	25	20
α-HOMO-2	-7.42	15	13	43	13	15
β-HOMO-2	-7.78	40	1	0	1	58

Table S10. Composition and energies of selected calculated (pbe1pbe) MOs of **DBTTF-(ViRu)$_{2Z}^{+}$**.

MO	eV	{Ru$_1$}	VinylBenz$_1$	TTF core	VinylBenz$_2$	{Ru$_2$}
α-LUMO	-1.86	12	9	57	9	13
β-LUMO	-4.33	3	14	67	14	3
α-HOMO	-5.59	12	24	29	24	12
β-HOMO	-5.59	19	29	5	29	19
α-HOMO-1	-5.80	20	28	3	28	20
β-HOMO-1	-5.91	20	22	17	22	20
α-HOMO-2	-6.30	12	13	50	13	12
β-HOMO-2	-6.62	73	1	0	0	27

Table S11. Composition and energies of selected calculated (pbe1pbe) occupied frontier MOs of **DBTTF-(ViRu)$_{2E}^{2+}$** in the closed-shell singlet (S) state.

MO	eV	{Ru$_1$}	VinylBenz$_1$	TTF core	VinylBenz$_2$	{Ru$_2$}
HOMO	-6.02	20	28	4	28	20
HOMO-1	-6.66	17	17	32	17	17
HOMO-2	-7.00	49	1	0	1	49

Table S12. Composition and energies of selected calculated (pbe1pbe) occupied frontier MOs of **DBTTF-(ViRu)$_{2E}^{2+}$** in the triplet (T) state.

MO	eV	{Ru$_1$}	VinylBenz$_1$	TTF core	VinylBenz$_2$	{Ru$_2$}
α-HOMO	-5.95	3	14	67	14	3
β-HOMO	-5.96	10	14	52	14	10
α-HOMO-1	-6.66	20	27	6	27	20
β-HOMO-1	-7.18	48	1	1	1	48
α-HOMO-2	-6.87	22	19	18	19	22
β-HOMO-2	-7.18	49	1	1	1	49

Table S13. Composition and energies of selected calculated (pbe1pbe) occupied frontier MOs of **DBTTF-(ViRu)$_{2E}^{2+}$** in the open-shell singlet (OSS) state.

MO	eV	{Ru$_1$}	VinylBenz$_1$	TTF core	VinylBenz$_2$	{Ru$_2$}
α-HOMO	-6.01	12	17	53	14	3
β-HOMO	-6.01	3	14	53	17	12
α-HOMO-1	-6.70	2	3	12	43	40
β-HOMO-1	-6.70	40	43	12	3	2
α-HOMO-2	-7.14	97	2	0	0	1
β-HOMO-2	-7.14	1	0	0	2	97

Table S94. Composition and energies of selected M062X-calculated occupied frontier MOs of **DBTTF-(ViRu)$_{2E}^{2+}$** in the closed-shell singlet (S) state.

MO	eV	{Ru$_1$}	VinylBenz$_1$	TTF core	VinylBenz$_2$	{Ru$_2$}
HOMO	-7.10	19	28	4	29	20
HOMO-1	-7.42	22	22	14	21	21
HOMO-2	-8.06	53	2	0	2	43

Table S15. Composition and energies of selected M062X-calculated occupied frontier MOs of **DBTTF-(ViRu)$_{2E}^{2+}$** in the triplet (T) state.

MO	eV	{Ru$_1$}	VinylBenz$_1$	TTF core	VinylBenz$_2$	{Ru$_2$}
α-HOMO	-6.95	26	47	24	3	0
β-HOMO	-7.04	38	52	8	1	0
α-HOMO-1	-7.47	18	47	24	3	0
β-HOMO-1	-7.89	97	3	0	0	0
α-HOMO-2	-7.90	94	4	1	1	1
β-HOMO-2	-8.32	96	4	0	0	0

Table S16. Composition and energies of selected M062X-calculated occupied frontier MOs of **DBTTF-(ViRu)$_{2E}^{2+}$** in the open-shell singlet (OSS) state.

MO	eV	{Ru$_1$}	VinylBenz$_1$	TTF core	VinylBenz$_2$	{Ru$_2$}
α-HOMO	-6.79	5	11	69	14	2
β-HOMO	-6.79	2	14	69	11	5
α-HOMO-1	-7.80	1	4	13	45	37
β-HOMO-1	-7.80	37	45	13	4	1
α-HOMO-2	-8.40	91	4	2	1	2
β-HOMO-2	-8.41	3	1	2	4	90

Table S17. Composition and energies of selected calculated (pbe1pbe) occupied frontier MOs of **DBTTF-(ViRu)$_{2Z}$$^{2+}$** in the closed-shell singlet (S) state.

MO	eV	{Ru$_1$}	VinylBenz$_1$	TTF core	VinylBenz$_2$	{Ru$_2$}
HOMO	-6.01	20	28	4	28	20
HOMO-1	-6.50	17	17	31	17	17
HOMO-2	-6.99	54	1	0	1	44

Table S18. Composition and energies of selected calculated (pbe1pbe) occupied frontier MOs of **DBTTF-(ViRu)$_{2Z}$$^{2+}$** in the triplet (T) state.

MO	eV	{Ru$_1$}	VinylBenz$_1$	TTF core	VinylBenz$_2$	{Ru$_2$}
α-HOMO	-5.98	3	14	67	14	3
β-HOMO	-5.98	10	15	50	15	10
α-HOMO-1	-6.65	19	27	7	27	19
β-HOMO-1	-7.18	48	1	1	1	48
α-HOMO-2	-6.90	24	18	16	18	24
β-HOMO-2	-7.19	49	1	0	1	49

Table S19. Composition and energies of selected calculated (pbe1pbe) occupied frontier MOs of **DBTTF-(ViRu)$_{2Z}$$^{2+}$** in the open-shell singlet (OSS) state.

MO	eV	{Ru$_1$}	VinylBenz$_1$	TTF core	VinylBenz$_2$	{Ru$_2$}
α-HOMO	-6.01	12	18	53	14	3
β-HOMO	-6.01	3	15	53	17	12
α-HOMO-1	-6.72	2	3	12	43	40
β-HOMO-1	-6.71	40	43	12	3	2
α-HOMO-2	-7.14	97	2	0	0	1
β-HOMO-2	-7.15	1	0	0	2	96

Table S20. NBO calculated (pbe1pbe) atomic partial charges for the individual constituents of the complexes **DBTTF-(ViRu)$_{2E}$** and **DBTTF-(ViRu)$_{2Z}$** in their one-electron and two-electron (singlet, triplet and OSS) oxidized states.

	State	{Ru$_1$}	VinylBenz$_1$	TTF core	VinylBenz$_2$	{Ru$_2$}
**DBTTF-(ViRu)$_{2Z}$**						
	0	0.13	-0.47	0.69	-0.47	0.13
	+1	0.19	-0.35	1.33	-0.35	0.19
	+2$_{singlet}$	0.33	-0.17	1.67	-0.17	0.33
	+2$_{triplet}$	0.50	-0.07	1.16	-0.07	0.50
	+2$_{OSS}$	0.47	-0.09	1.14	-0.08	0.46
**DBTTF-(ViRu)$_{2E}$**						
	0	0.11	-0.48	0.73	-0.48	0.11
	+1	0.19	-0.35	1.33	-0.35	0.19
	+2$_{singlet}$	0.34	-0.17	1.67	-0.17	0.34
	+2$_{triplet}$	0.50	-0.07	1.14	-0.07	0.50
	+2$_{OSS}$	0.46	-0.09	1.24	-0.08	0.46

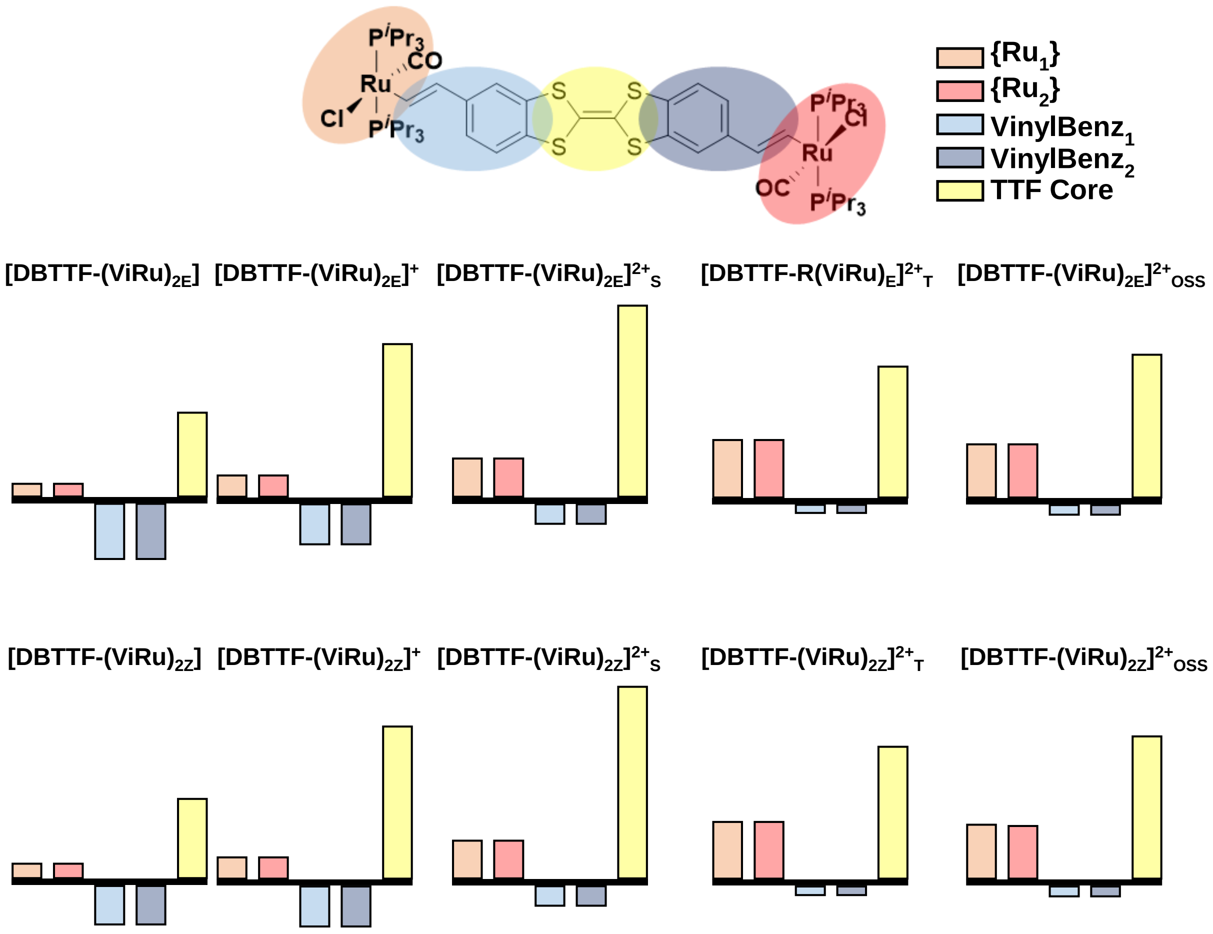

Figure S34. Schematic illustration of absolute NBO calculated atomic partial charges for the individual constituents of the complexes **DBTTF-(ViRu)$_{2E}$** and **DBTTF-(ViRu)$_{2Z}$** in their one-electron and two-electron (singlet, triplet and OSS) oxidized states.

Table S21. Differences of the NBO calculated (pbe1pbe) atomic partial charges for the individual constituents of the complexes **DBTTF-(ViRu)$_{2E}$** and **DBTTF-(ViRu)$_{2Z}$** upon oxidation. Differences are given relative to the neutral complex.

	State	{Ru$_1$}	VinylBenz$_1$	TTF core	VinylBenz$_2$	{Ru$_2$}
**DBTTF-(ViRu)$_{2Z}$**						
	+1	0.06	0.12	0.64	0.12	0.06
	+2$_{singlet}$	0.20	0.30	0.98	0.30	0.20
	+2$_{triplet}$	0.37	0.40	0.47	0.40	0.37
	+2$_{OSS}$	0.34	0.38	0.45	0.39	0.33
**DBTTF-(ViRu)$_{2E}$**						
	+1	0.08	0.13	0.60	0.13	0.08
	+2$_{singlet}$	0.23	0.31	0.91	0.31	0.23
	+2$_{triplet}$	0.39	0.41	0.41	0.41	0.39
	+2$_{OSS}$	0.35	0.39	0.51	0.40	0.35

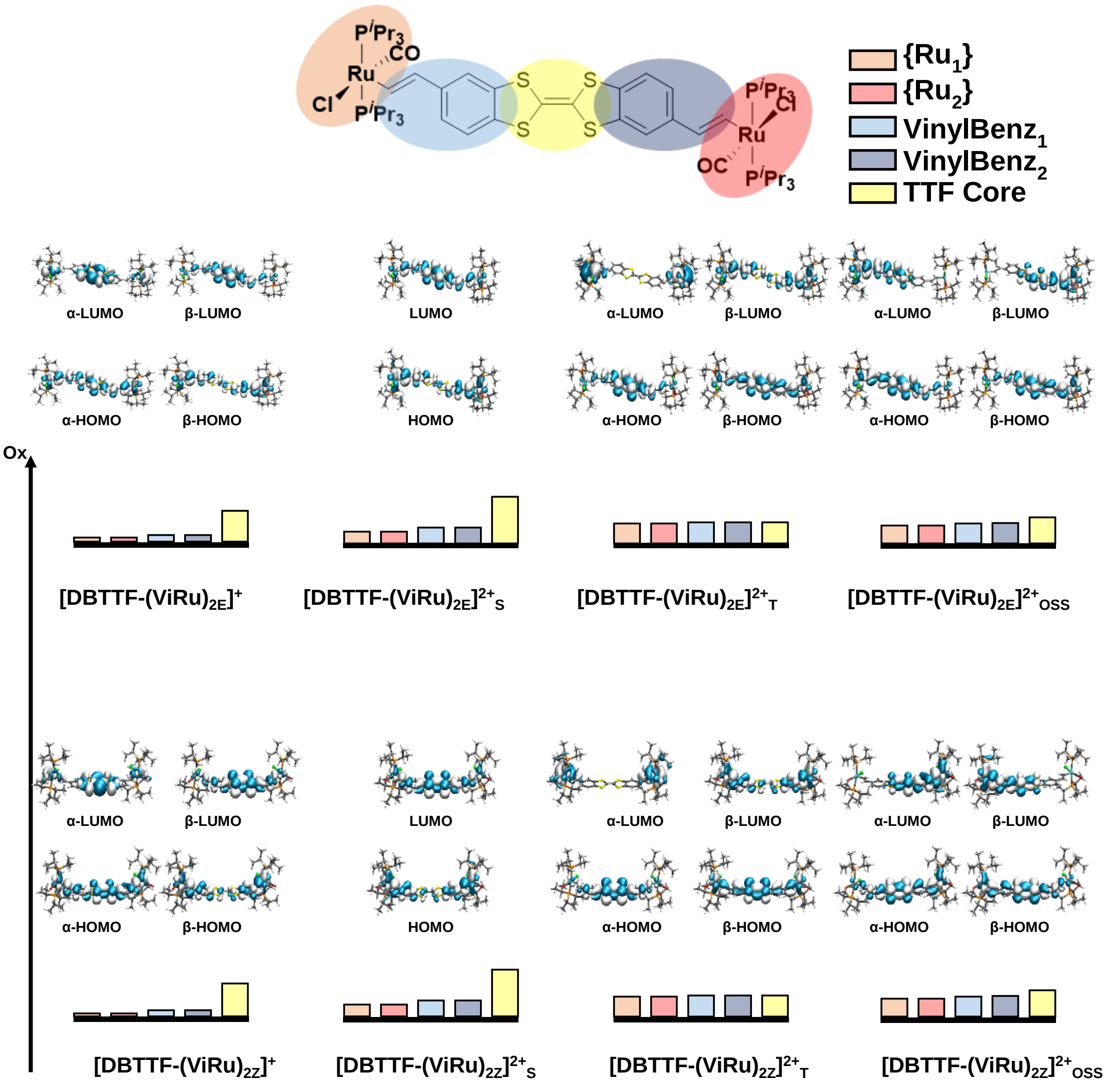

Figure S35. Differences in NBO calculated (pbe1pbe) atomic partial charges for the individual constituents of the complexes **DBTTF-(ViRu)$_{2E}$** and **DBTTF-(ViRu)$_{2Z}$** in their one-electron and two-electron (singlet, triplet and OSS) oxidized states. Differences are given relative to the neutral complex.

Table S22. NBO calculated (M062X) atomic partial charges for the individual constituents of the complexe **DBTTF-(ViRu)$_{2E}$** in its one-electron and two-electron (singlet, triplet and OSS) oxidized states.

	State	{Ru$_1$}	VinylBenz$_1$	TTF core	VinylBenz$_2$	{Ru$_2$}
**DBTTF-(ViRu)$_{2E}$**						
	0	0.10	-0.47	0.73	-0.47	0.10
	+1	0.14	-0.36	1.43	-0.36	0.14
	+2$_{singlet}$	0.22	-0.22	2.00	-0.22	0.22
	+2$_{triplet}$	0.16	-0.33	1.49	-0.04	0.71
	+2$_{OSS}$	0.53	-0.03	1.00	-0.03	0.53

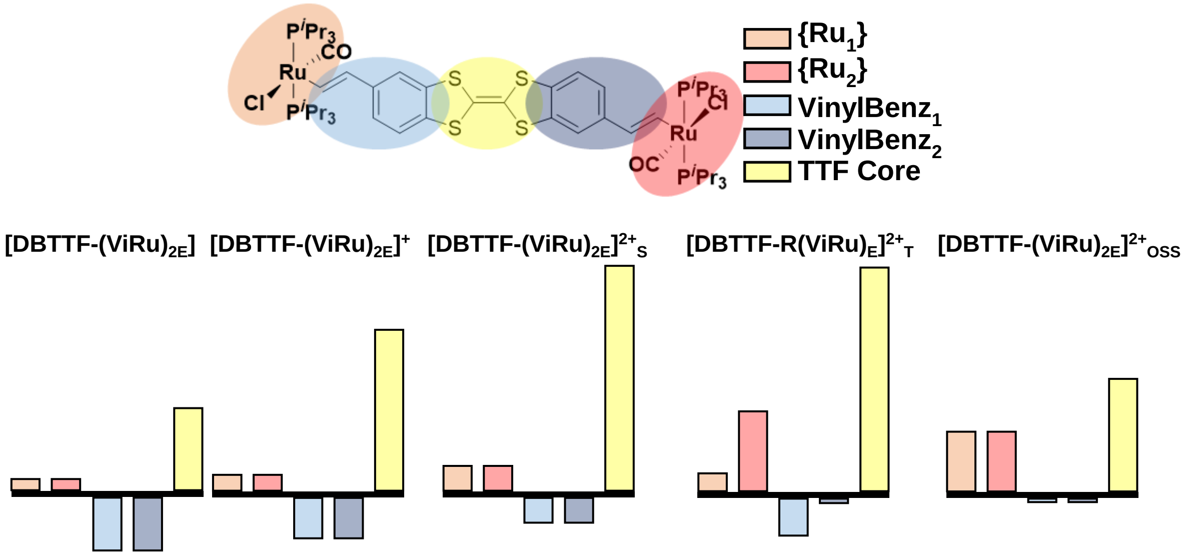

Figure S36. Schematic illustration of absolute NBO calculated (M062X) atomic partial charges for the individual constituents of the complexes **DBTTF-(ViRu)$_{2E}$** in its one-electron and two-electron (singlet, triplet and OSS) oxidized states.

Table S23. Differences of the NBO calculated (M062X) atomic partial charges for the individual constituents of the complexe **DBTTF-(ViRu)$_{2E}$** upon oxidation. Differences are given relative to the neutral complex.

	State	$\{Ru_1\}$	$VinylBenz_1$	TTF core	$VinylBenz_2$	$\{Ru_2\}$
**DBTTF-(ViRu)$_{2E}$**						
	+1	0.04	0.11	0.70	0.11	0.04
	$+2_{singlet}$	0.12	0.25	1.27	0.25	0.12
	$+2_{triplet}$	0.06	0.14	0.76	0.43	0.61
	$+2_{OSS}$	0.43	0.44	0.27	0.44	0.43

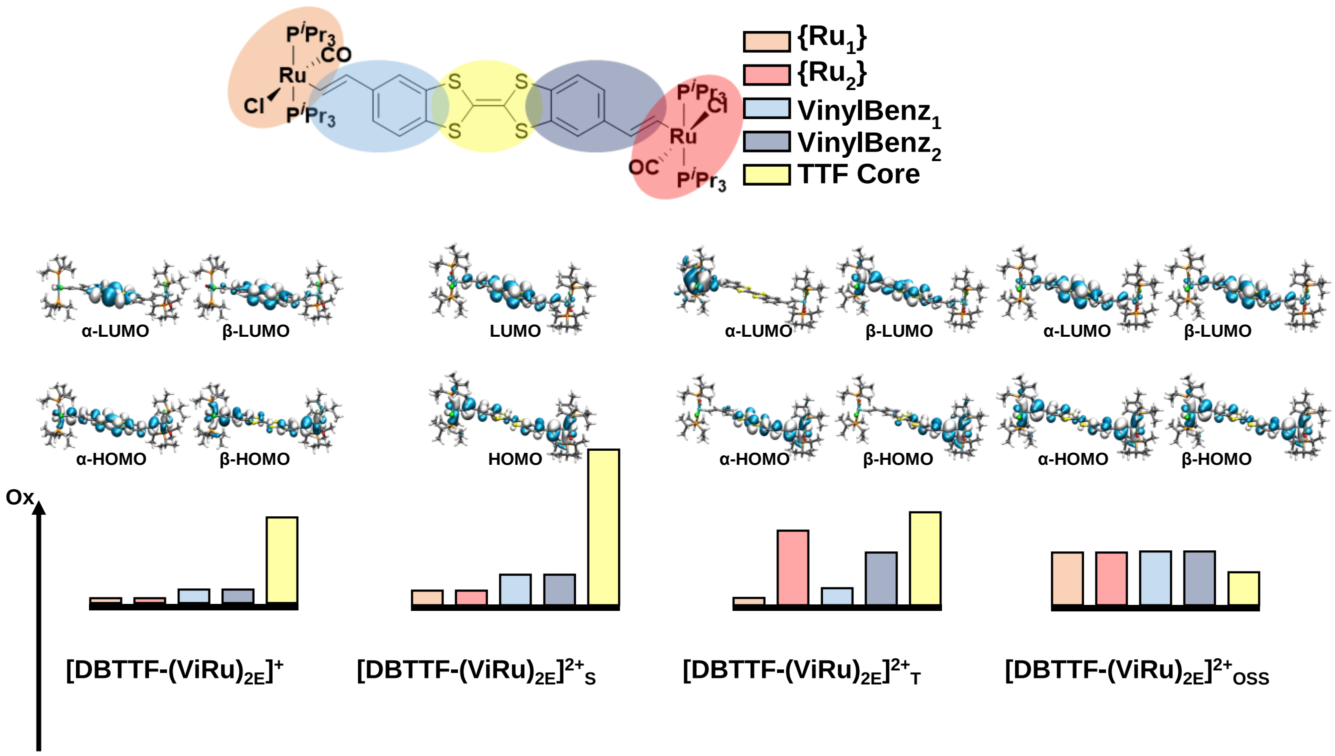

Figure S37. Differences in NBO calculated (M062X) atomic partial charges for the individual constituents of the complex **DBTTF-(ViRu)$_{2E}$** in its one-electron and two-electron (singlet, triplet and OSS) oxidized states and plots of the immediate frontier MOs. Charge differences are given relative to the neutral complex.

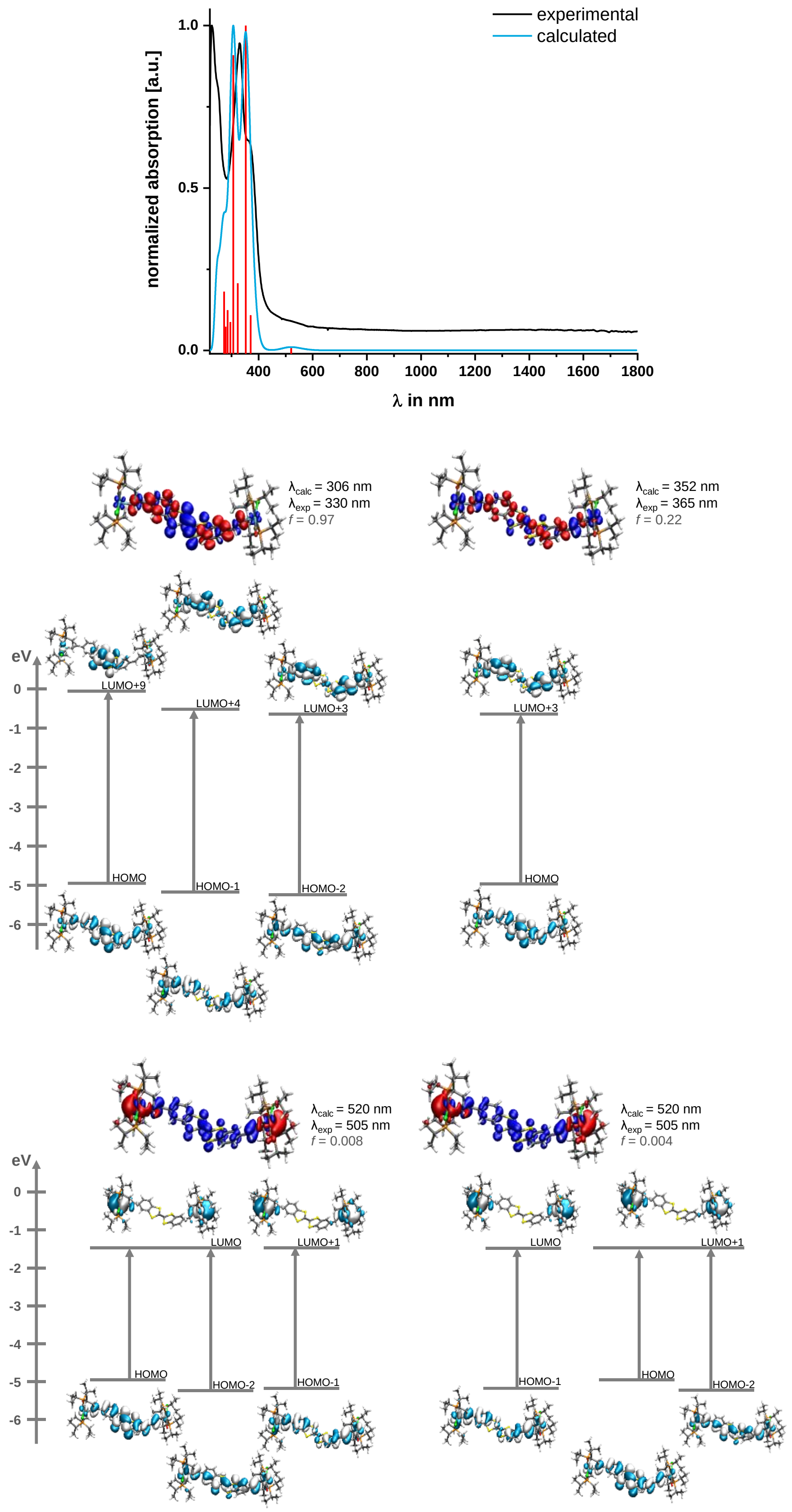

Figure S38. Top panel: Comparison of the experimental electronic spectrum of **DBTTF-(ViRu)$_2$** (black line) and the pbe1pbe TD-DFT calculated spectrum of **DBTTF-(ViRu)$_{2E}$** (blue line). Middle and bottom panels: Individual electronic transitions with the respective donor and acceptor molecular orbitals and the corresponding electron density difference maps (EDDMs; blue color = electron density decrease, red color = electron density increase).

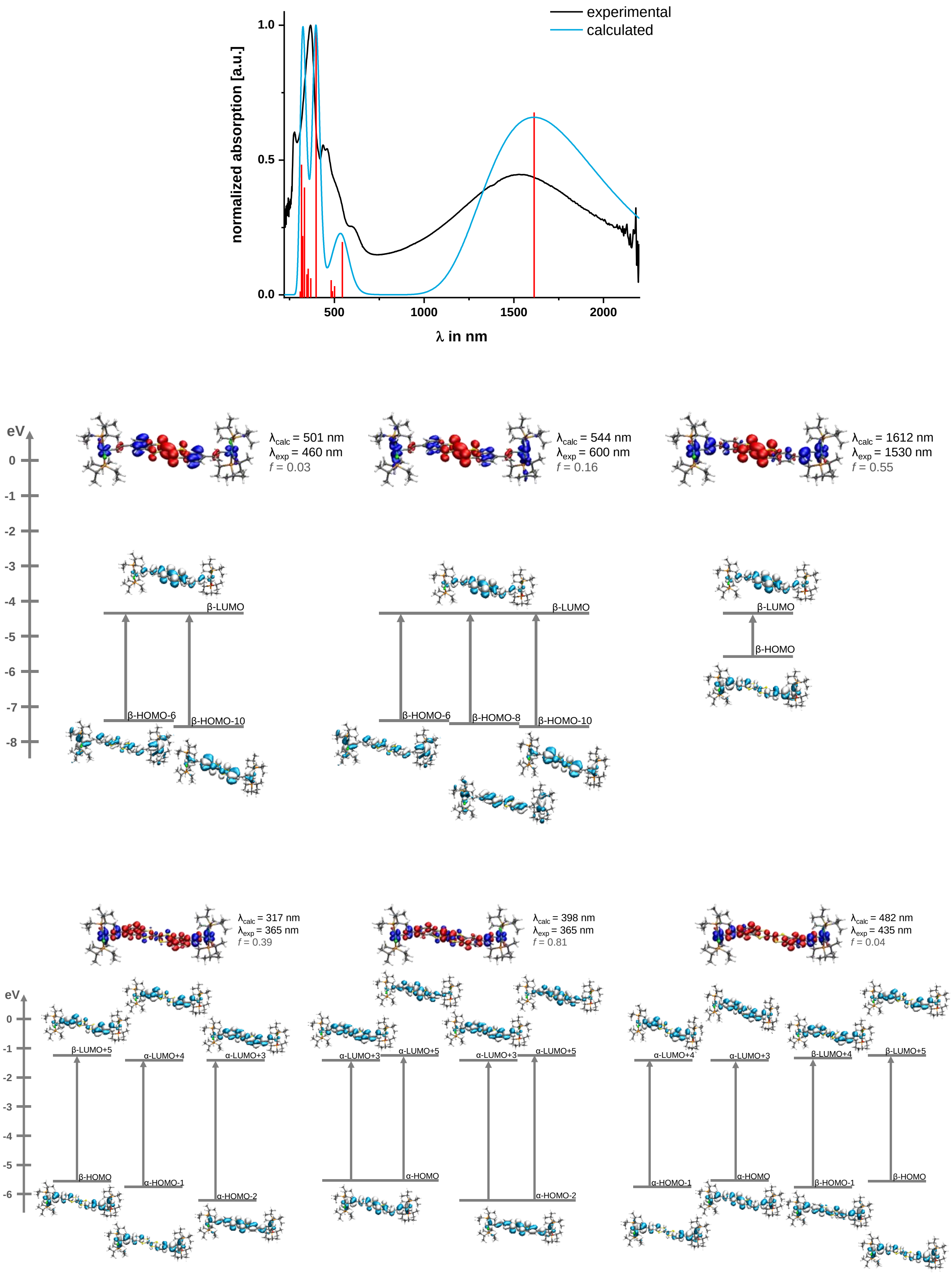

Figure S39. Top panel: Comparison of the experimental electronic spectrum (black line) of **DBTTF-(ViRu)$_{E2}^{+}$** and the pbe1pbe TD-DFT calculated spectrum (blue line) of **DBTTF-(ViRu)$_{2E}^{+}$**. Middle and bottom panels: Individual electronic transitions with the respective donor and acceptor molecular orbitals and the corresponding electron density difference maps (EDDMs; blue color = electron density decrease, red color = electron density increase).

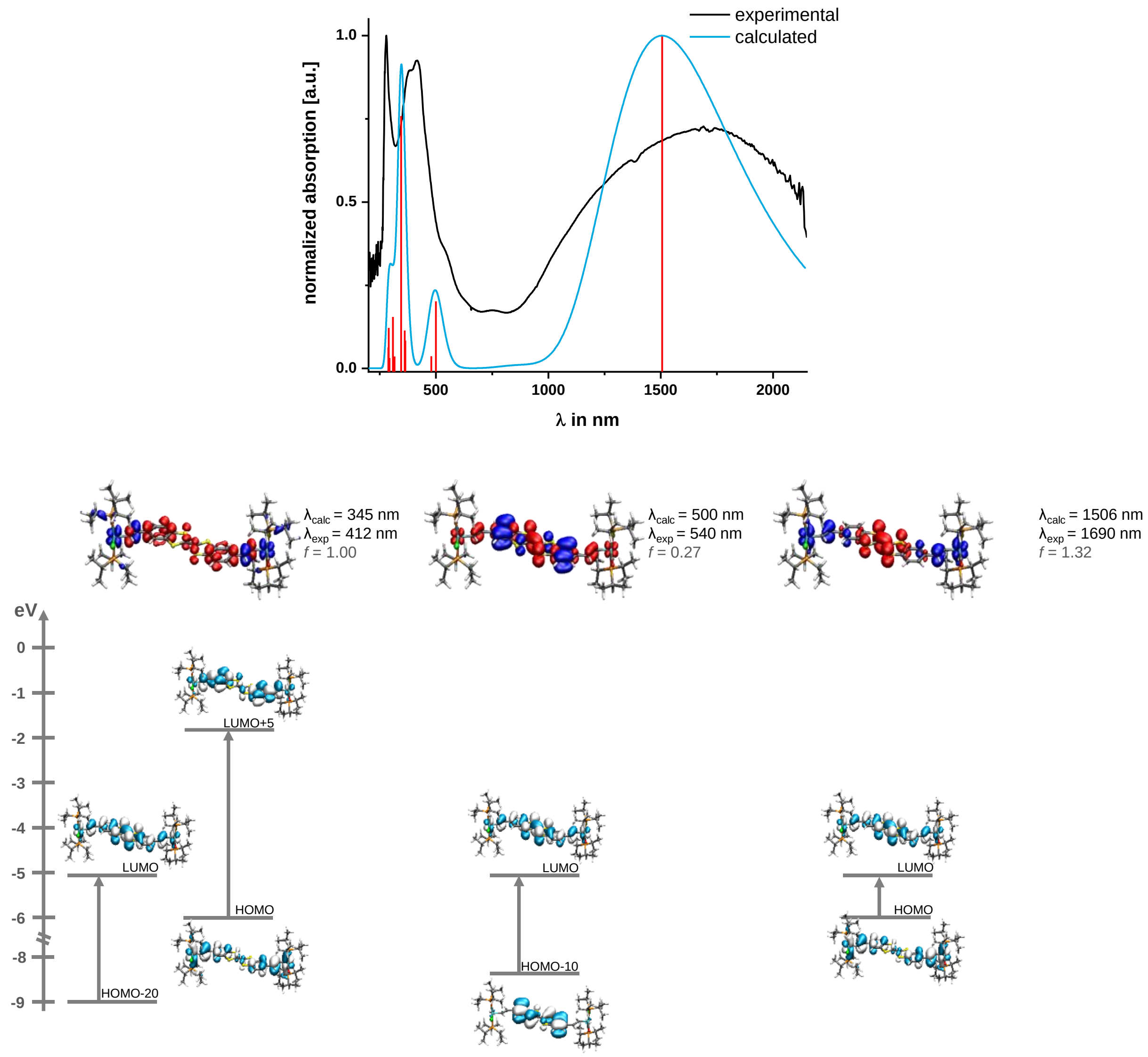

Figure S40. Top panel: Comparison of the experimental electronic spectrum (black line) of **DBTTF-(ViRu)$_2$$^{2+}$** and the pbe1pbe TD-DFT calculated spectrum (blue line) of **DBTTF-(ViRu)$_{2E}$$^{2+}$** in the singlet state. Bottom panel: Individual electronic transitions with the respective donor and acceptor molecular orbitals and the corresponding electron density difference maps (EDDMs; blue color = electron density decrease, red color = electron density increase).

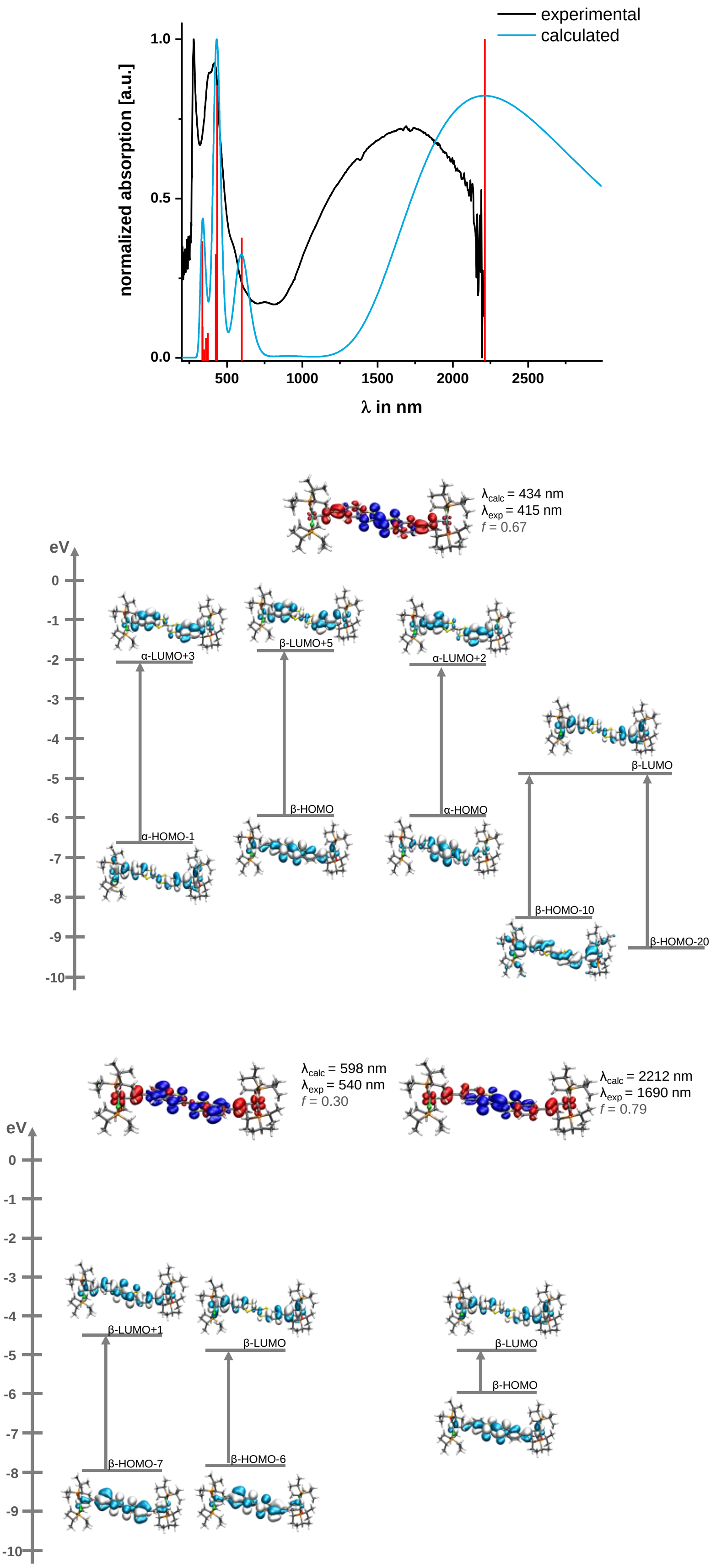

Figure S41. Top panel: Comparison of the experimental electronic spectrum (black line) of **DBTTF-(ViRu)$_2^{2+}$** and the pbe1pbe TD-DFT calculated spectrum (blue line) of **DBTTF-(ViRu)$_{2E}^{2+}$** in the triplet state. Middle and bottom panels: Individual electronic transitions with the respective donor and acceptor molecular orbitals and the corresponding electron density difference maps (EDDMs; blue color = electron density decrease, red color = electron density increase).

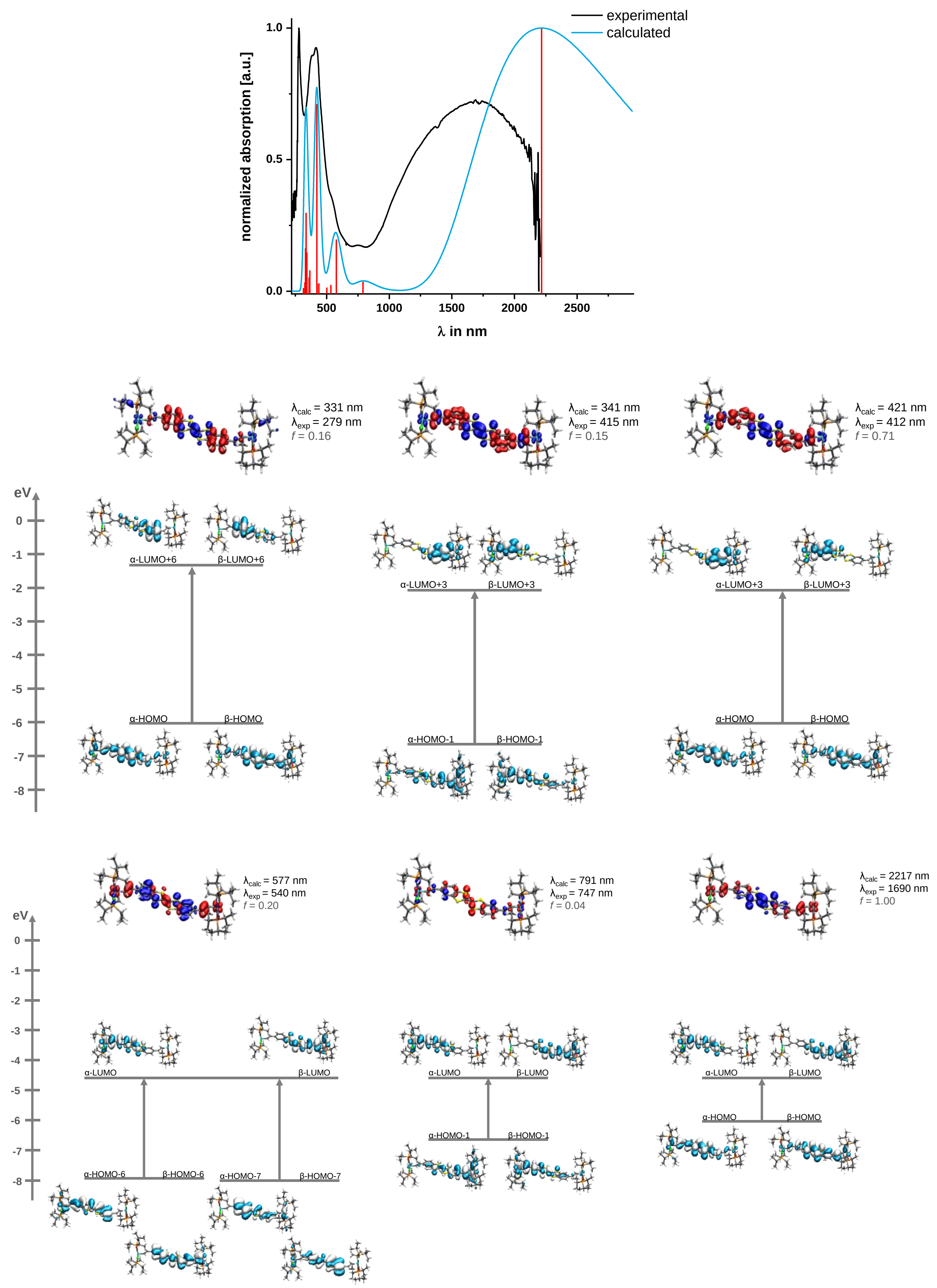

Figure S42. Top panel: Comparison of the experimental electronic spectrum (black line) of **DBTTF-(ViRu)$_2$$^{2+}$** and the pbe1pbe TD-DFT calculated spectrum (blue line) of **DBTTF-(ViRu)$_{2E}$$^{2+}$** in the open-shell singlet state. Middle and bottom panels: Individual electronic transitions with the respective donor and acceptor molecular orbitals and the corresponding electron density difference maps (EDDMs; blue color = electron density decrease, red color = electron density increase).

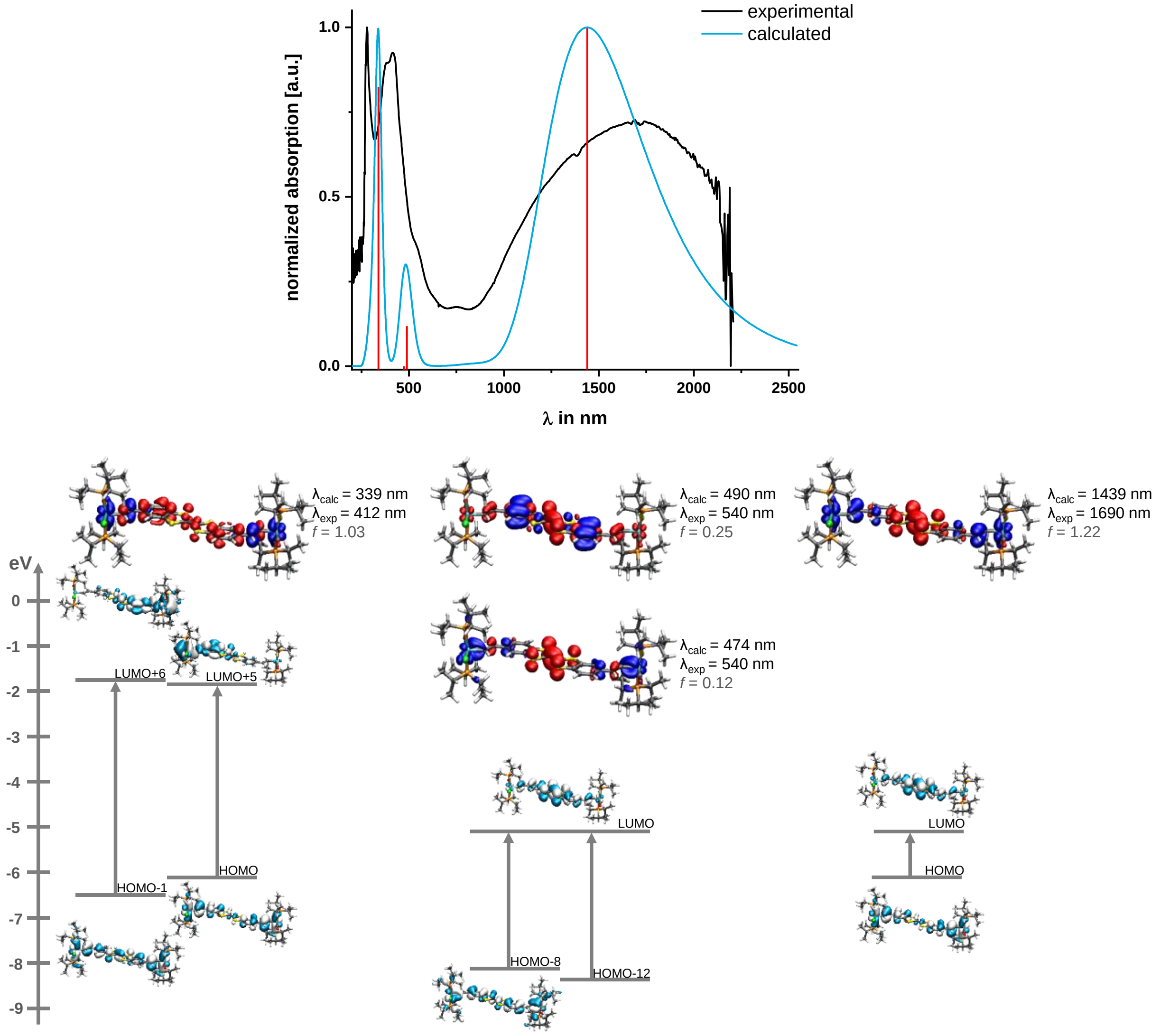

Figure S43. Top panel: Comparison of the experimental electronic spectrum (black line) of **DBTTF-(ViRu)$_2$$^{2+}$** and the M062X / pbe1pbe TD-DFT calculated spectrum (blue line) of **DBTTF-(ViRu)$_{2E}$$^{2+}$** in the singlet state. Bottom panel: Individual electronic transitions with the respective donor and acceptor molecular orbitals and the corresponding electron density difference maps (EDDMs; blue color = electron density decrease, red color = electron density increase).

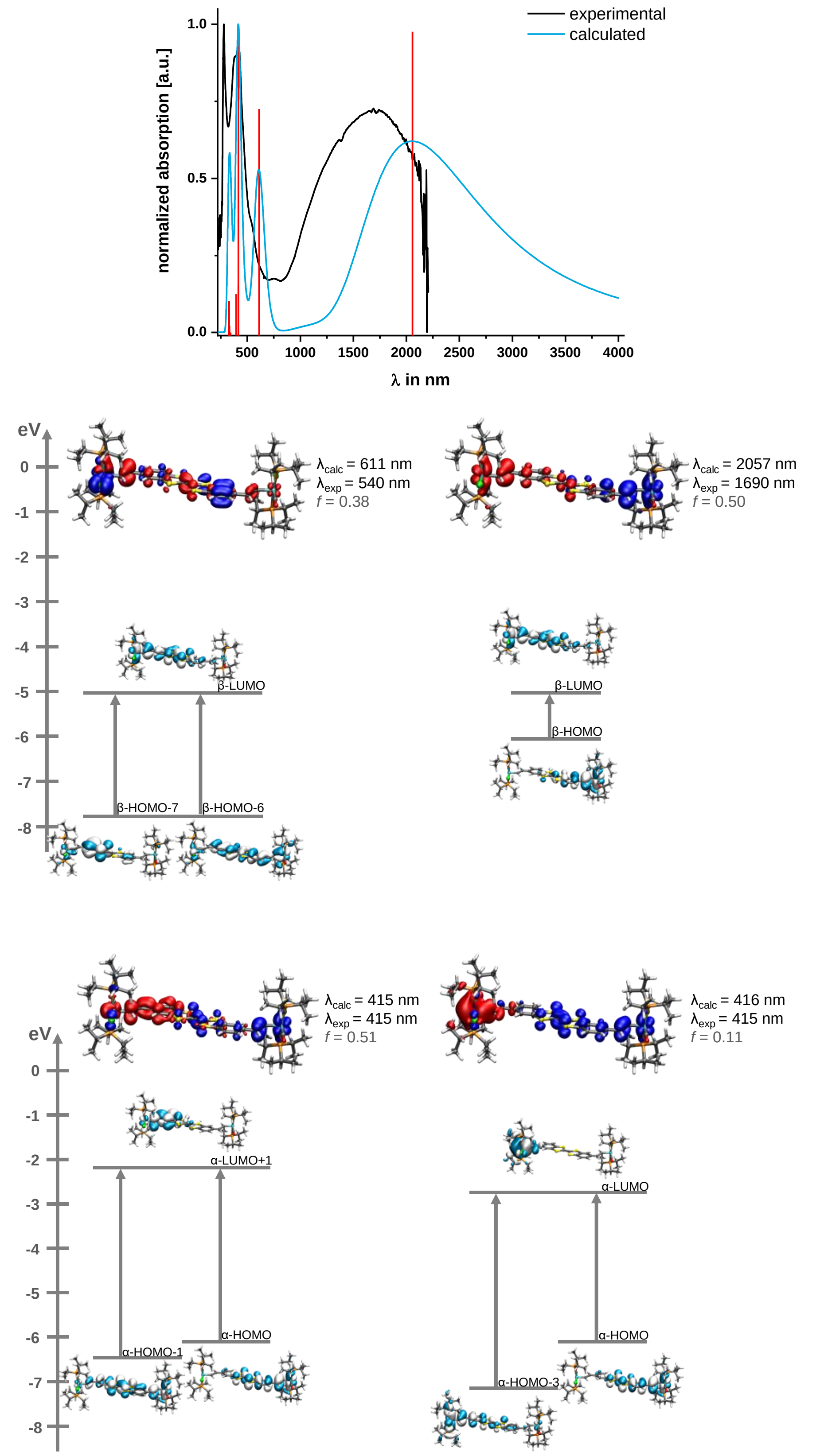

Figure S44. Top panel: Comparison of the experimental electronic spectrum (black line) of **DBTTF-(ViRu)$_2$$^{2+}$** and the M062X / pbe1pbe TD-DFT calculated spectrum (blue line) of **DBTTF-(ViRu)$_{2E}$$^{2+}$** in the triplet state. Middle and bottom panels: Individual electronic transitions with the respective donor and acceptor molecular orbitals and the corresponding electron density difference maps (EDDMs; blue color = electron density decrease, red color = electron density increase).

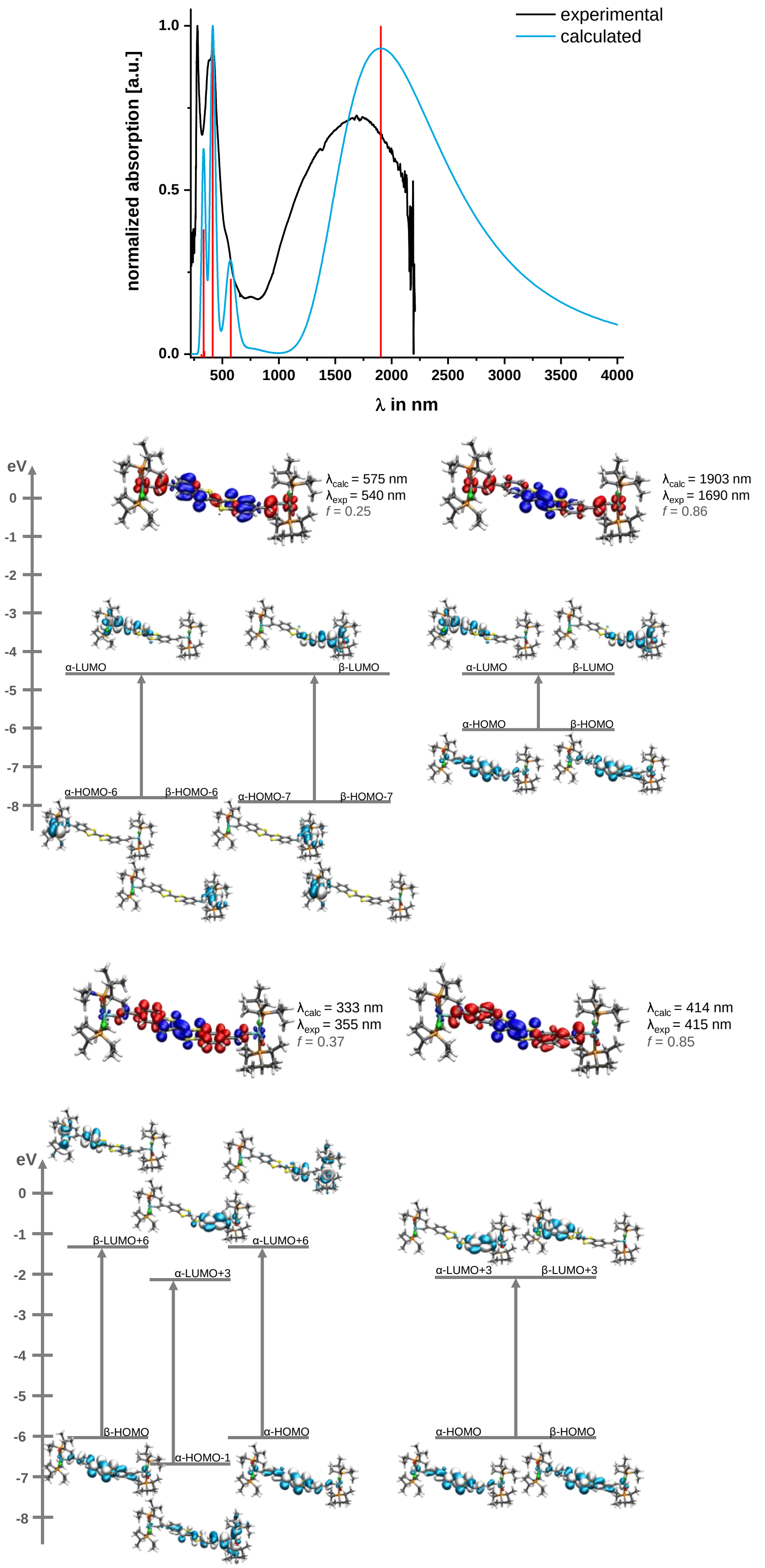

Figure S45. Top panel: Comparison of the experimental electronic spectrum (black line) of **DBTTF-(ViRu)$_2^{2+}$** and the M062X / pbe1pbe TD-DFT calculated spectrum (blue line) of **DBTTF-(ViRu)$_{2E}^{2+}$** in the open-shell singlet state. Middle and bottom panels: Individual electronic transitions with the respective donor and acceptor molecular orbitals and the corresponding electron density difference maps (EDDMs; blue color = electron density decrease, red color = electron density increase).

Electronic Spectra of Acceptors

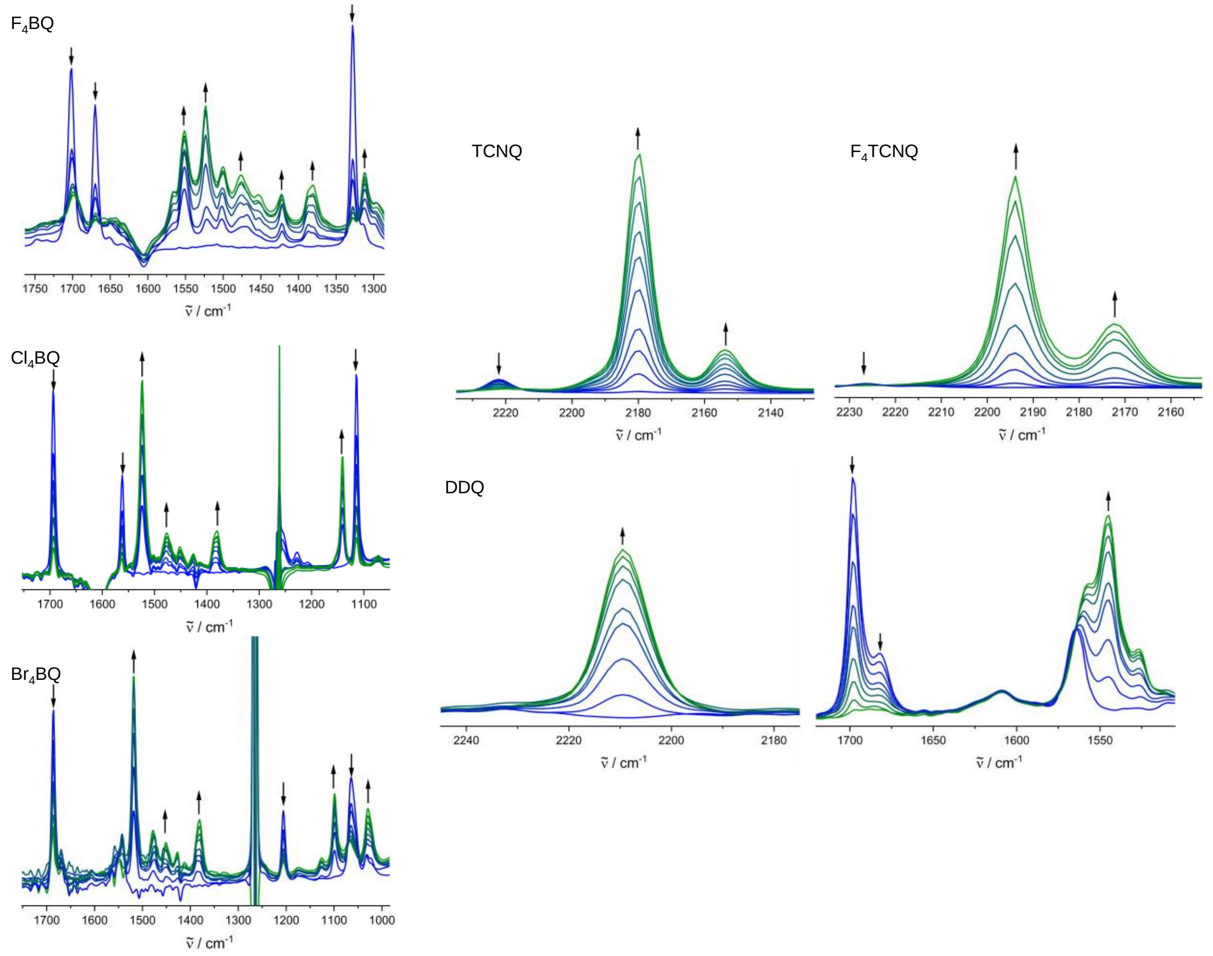

Figure S46. Left: Changes in IR spectra during chemical reduction of F_4BQ, Cl_4BQ and Br_4BQ with 0.00, 0.25, 0.05, 0.75, 1.00, 1.25, 1.5, 1.75 and 2.00 equivalents of Cp^*_2Fe in CH_2Cl_2 (blue to green); right: hanges in IR spectra during electrochemical reduction of TCNQ (top left), F_4TCNQ (top right) and DDQ (bottom) in 1,2-$C_2H_4Cl_2$ / 0.2 M $^nBu_4N^+$ PF_6^-, r.t.

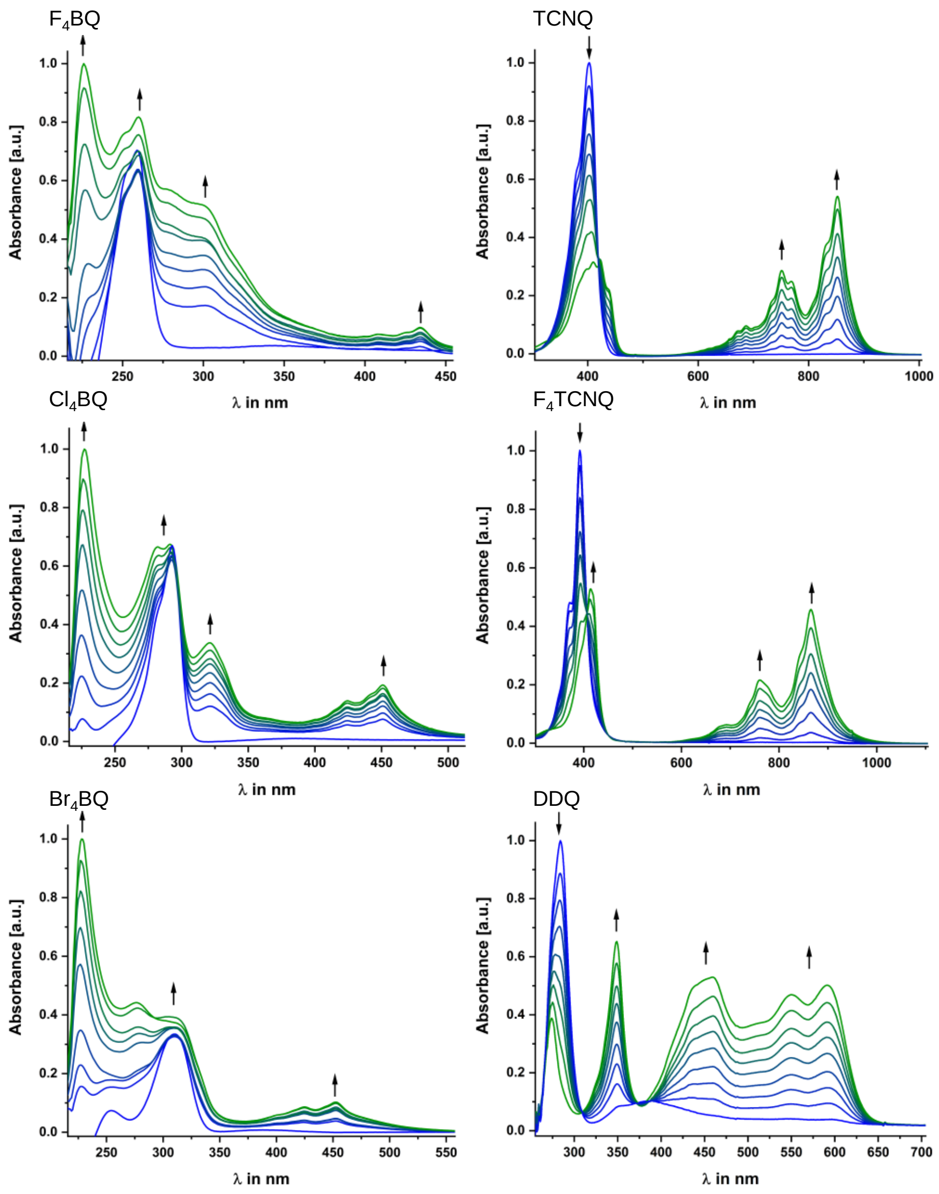

Figure S47. Left: Changes in UV/Vis spectra during chemical reduction of F_4BQ, Cl_4BQ and Br_4BQ with 0.00, 0.25, 0.05, 0.75, 1.00, 1.25, 1.5, 1.75 and 2.00 equivalents of Cp^*_2Fe in CH_2Cl_2 (blue to green); right: changes in IR spectra during electrochemical reduction of TCNQ (top), F_4TCNQ (middle) and DDQ (bottom) in 1,2-$C_2H_4Cl_2$ / 0.2 M $^nBu_4N^+$ PF_6^-, r.t.

Redox Reactions between DBTTF(ViRu)$_2$ and the Organic Acceptors

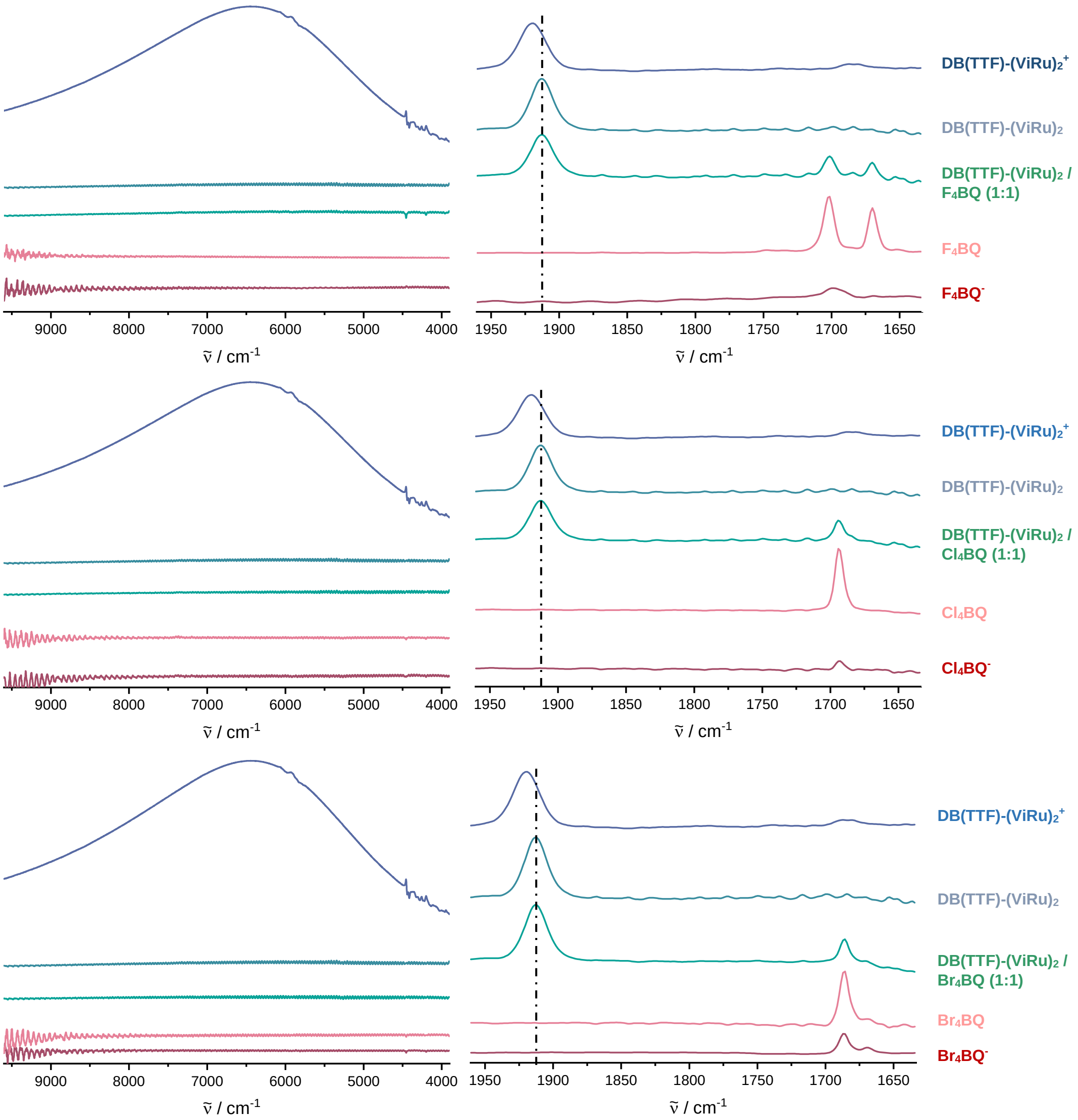

Figure S48. NIR (left) and IR (right) spectra obtained for 1:1 mixtures of **DBTTF-(ViRu)$_2$** and F_4BQ (top), Cl_4BQ (middle) and Br_4BQ (bottom) and comparison with the spectra of **DBTTF-(ViRu)$_2$** and **DBTTF-(ViRu)$_2^{+}$** and the neutral and reduced forms of the respective acceptor X_4BQ (X = F, Cl, Br). The black dotted lines serve as guide to the eye.

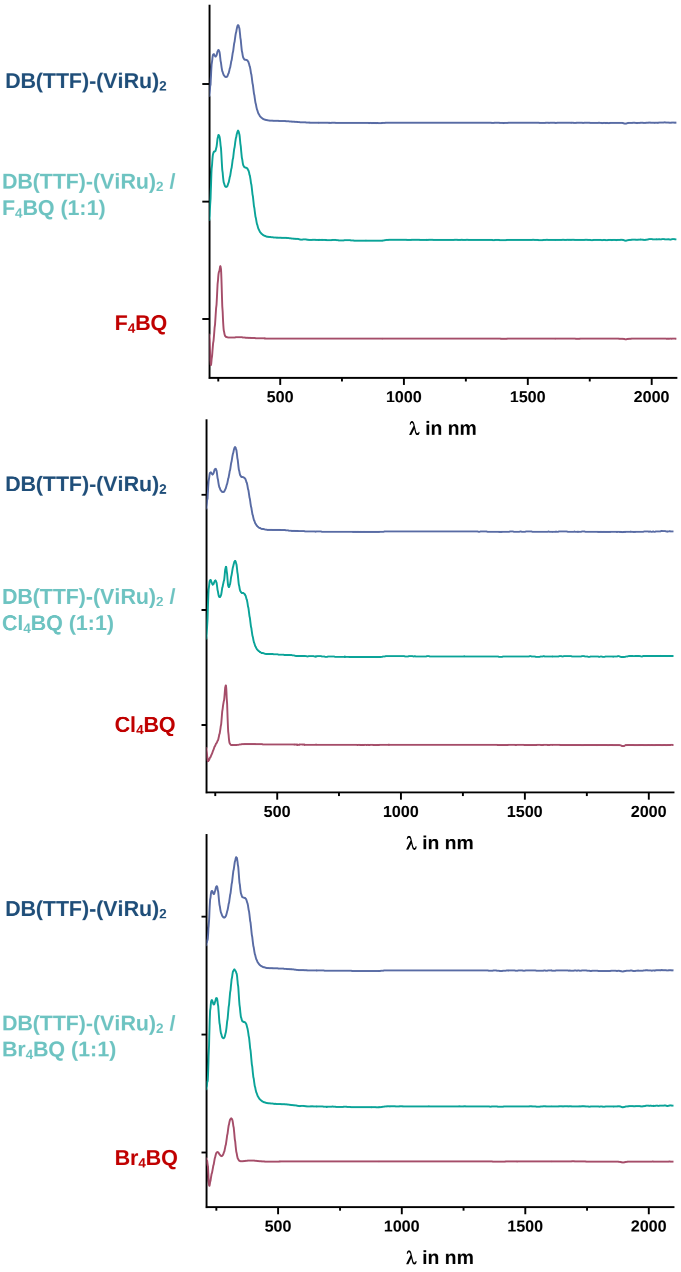

Figure S49. UV/Vis/NIR spectra obtained for 1:1 mixtures of **DBTTF-(ViRu)$_2$** and F_4BQ (top), Cl_4BQ (middle) and Br_4BQ (bottom) and comparison with the spectra of **DBTTF-(ViRu)$_2$** and the respective neutral acceptor.

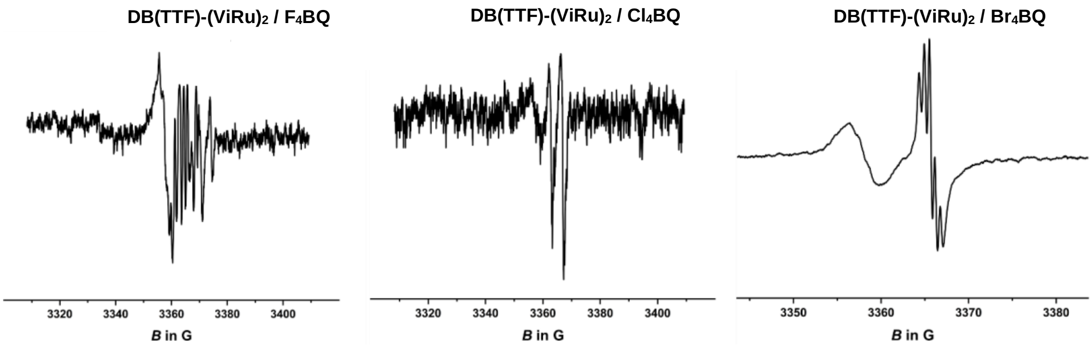

Figure S50. Solution EPR spectra obtained for 1:1 mixtures of **DBTTF-(ViRu)$_2$** and F_4BQ (left), **DBTTF-(ViRu)$_2$** and Cl_4BQ (middle), and **DBTTF-(ViRu)$_2$** and Br_4BQ (right).

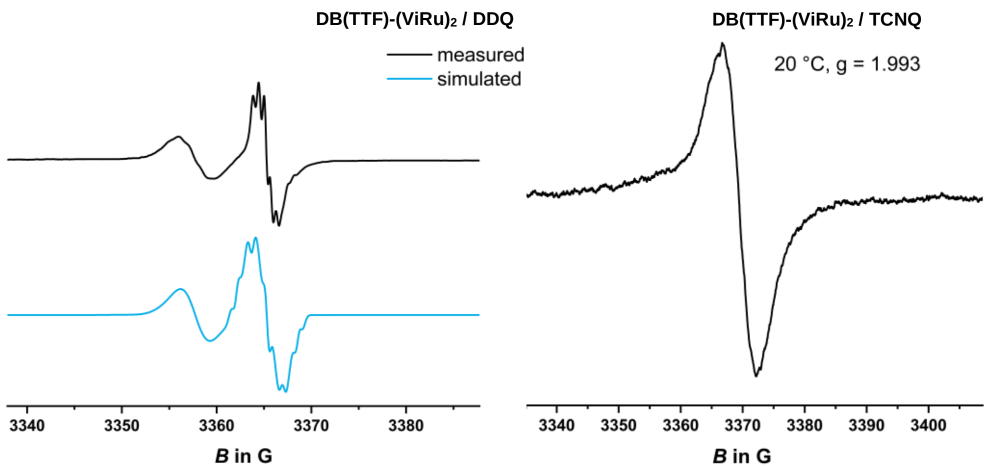

Figure S51. Solution EPR spectra obtained for 1:1 mixtures of **DBTTF-(ViRu)$_2$** with DDQ (left; black line: experimental, blue line: simulated spectrum) and TCNQ (right).

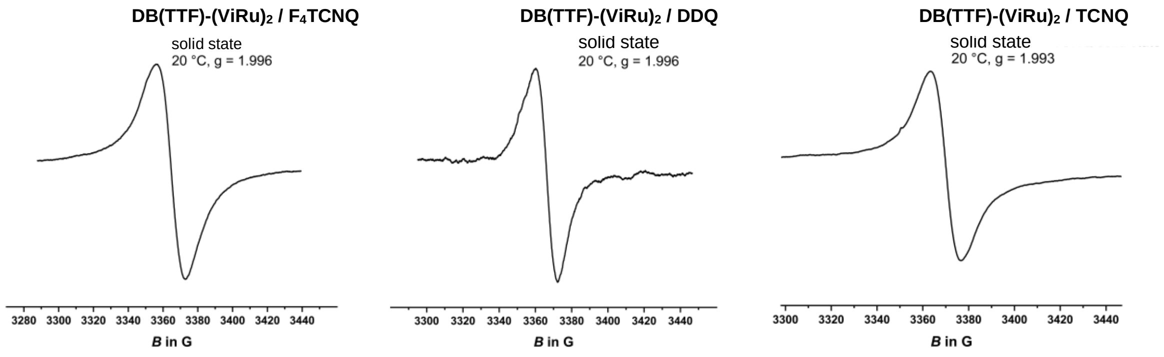

Figure S52. Solid state EPR spectra obtained for 1:1 mixtures of **DBTTF-(ViRu)$_2$** with F$_4$TCNQ (left), DDQ (middle) and TCNQ (right).

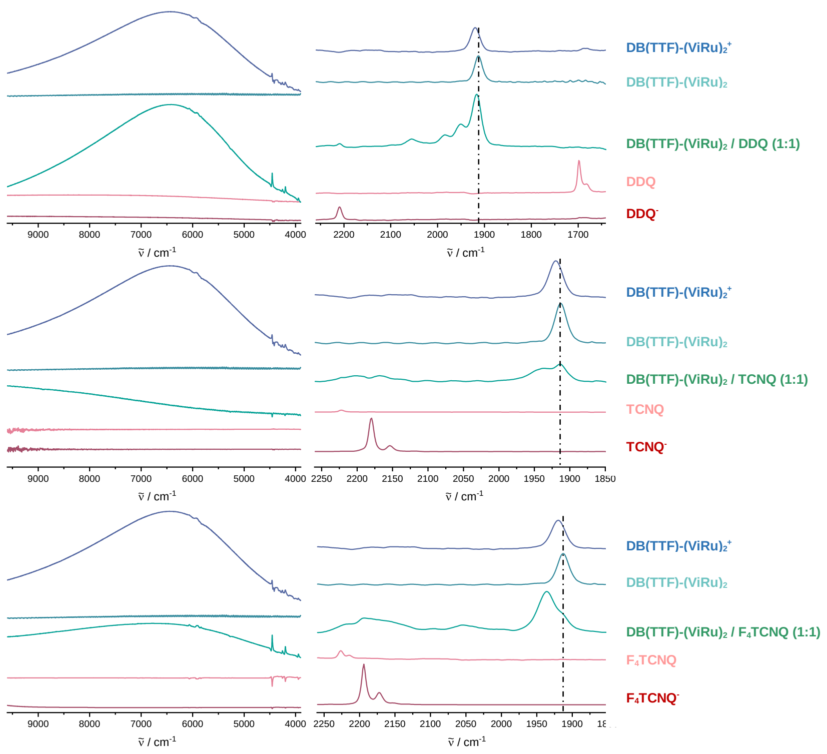

Figure S53. NIR (left) and IR (right) spectra obtained for 1:1 mixtures of **DBTTF-(ViRu)$_2$** and DDQ (top), TCNQ (middle) and F$_4$TCNQ (bottom) and comparison with the spectra of **DBTTF-(ViRu)$_2$** and **DBTTF-(ViRu)$_2^+$** and the neutral and reduced forms of the respective acceptor.the black dotted lines serve as guide to the eye.

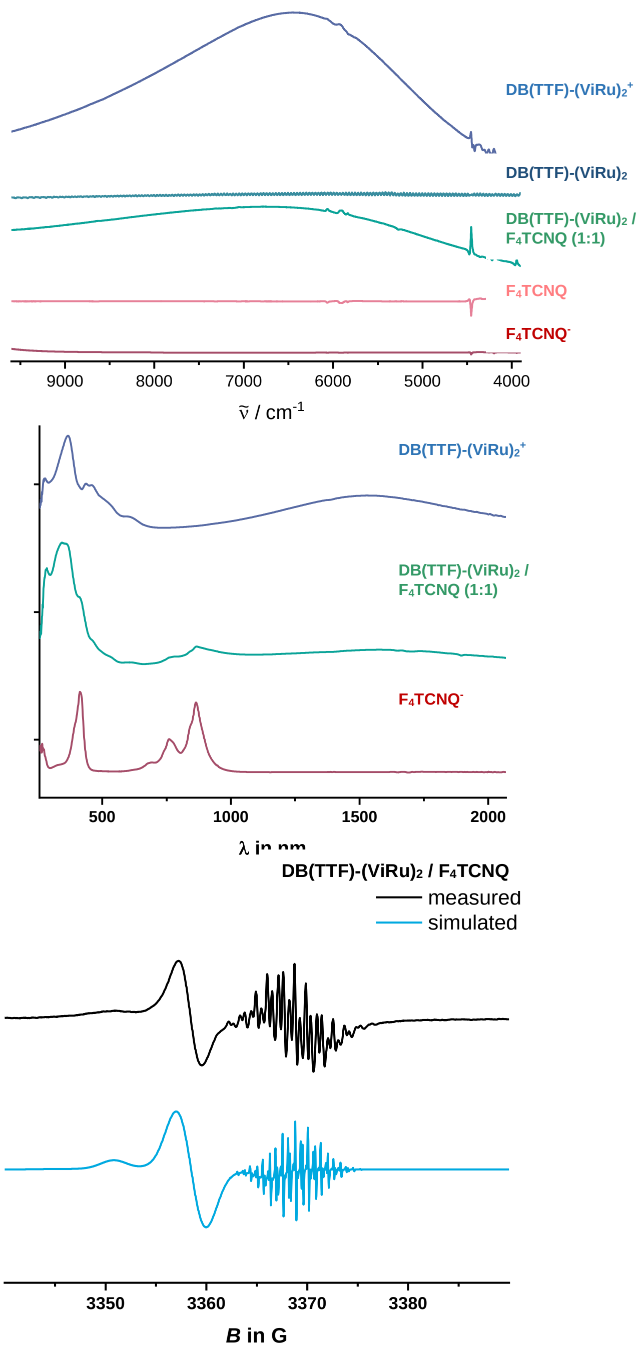

Figure S54. Comparison of UV/Vis/NIR spectra (middle) of solutions of **DBTTF-(ViRu)$_2$**, **DBTTF-(ViRu)$_2^+$**, F_4TCNQ, F_4TCNQ^- and of a 1:1 mixture of **DBTTF-(ViRu)$_2$** and F_4TCNQ in CH_2Cl_2. Bottom: Experimental (black line) and simulated (blue line) solution EPR spectrum of **DBTTF-(ViRu)$_2$** : F_4TCNQ (1:1).

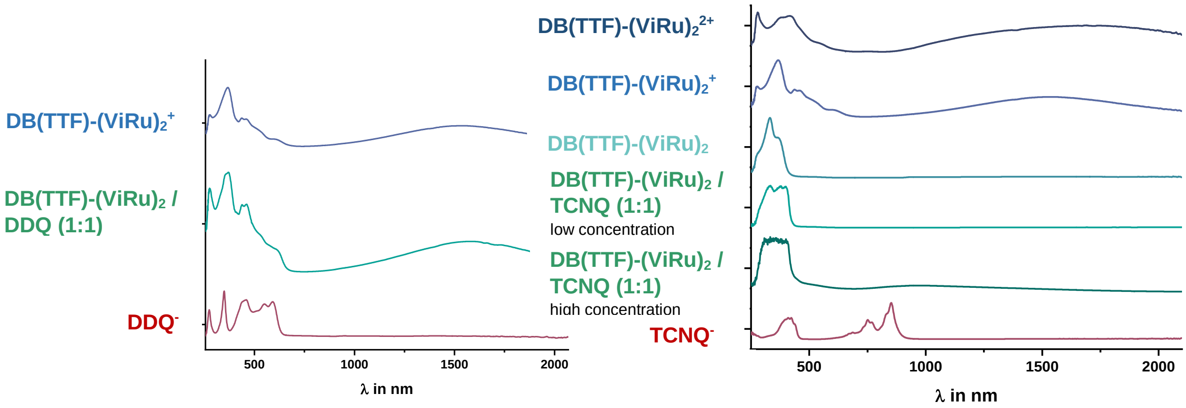

Figure S55. UV/Vis/NIR spectra obtained for 1:1 mixtures of **DBTTF-(ViRu)$_2$** and DDQ (left) or TCNQ (right) and comparison with the spectra obtained for **DBTTF-(ViRu)$_2^+$** and the respective reduced acceptor.

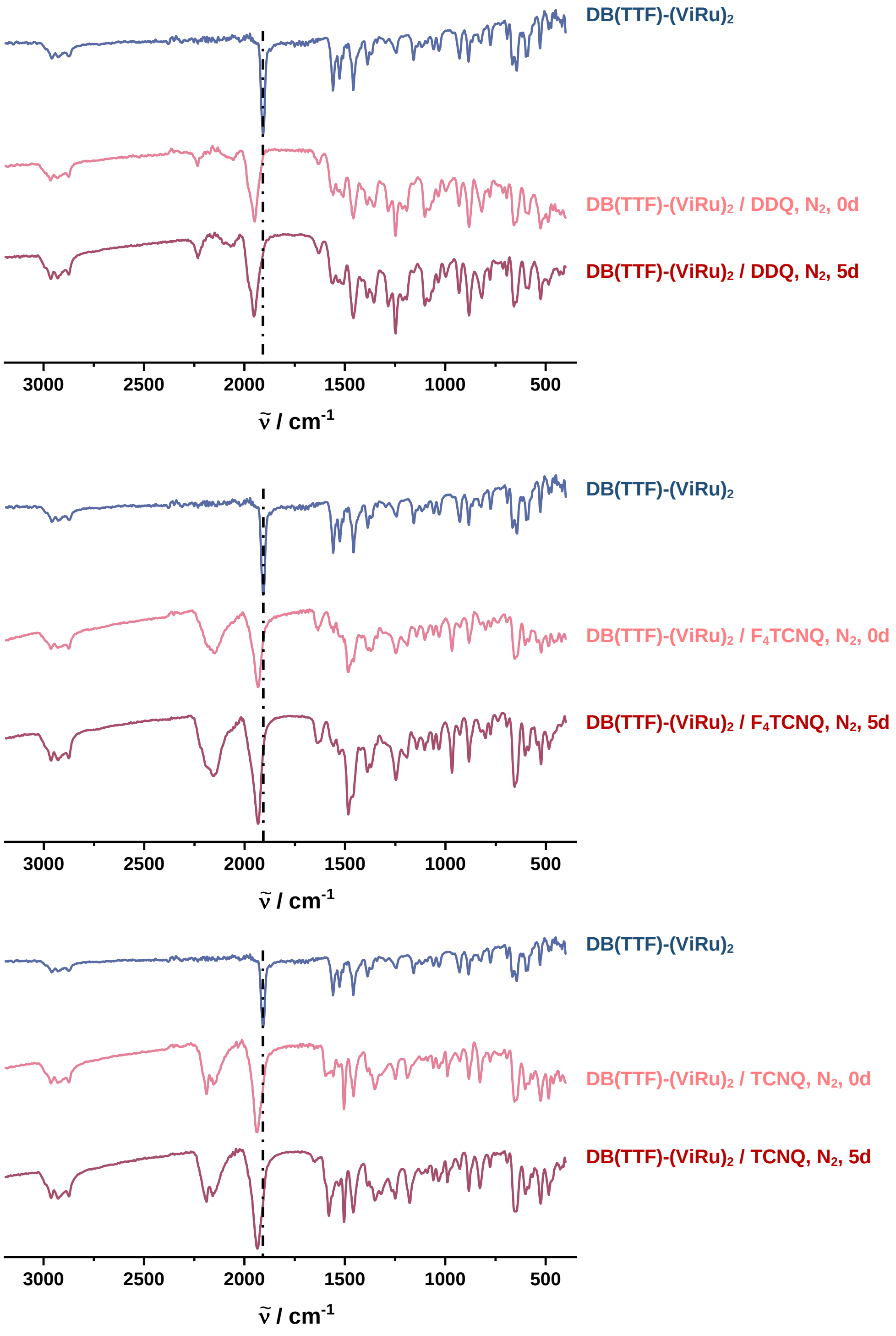

Figure S56. ATR IR spectra obtained for 1:1 mixtures of **DBTTF-(ViRu)$_2$** and DDQ (top), F_4TCNQ (middle) and TCNQ (bottom) measured directly after synthesis and after storage for 5 days under nitrogen atmosphere and comparison with the spectrum of **DBTTF-(ViRu)$_2$**.

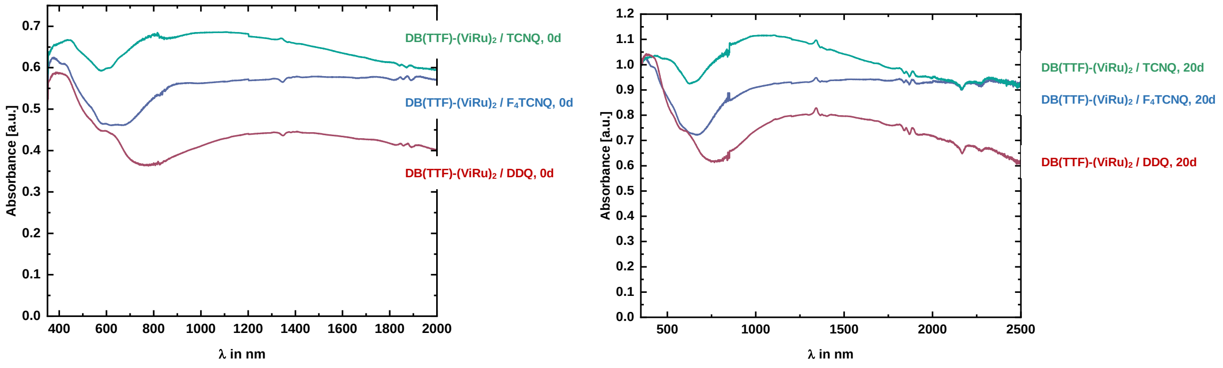

Figure S57. Solid state UV/Vis/NIR spectra obtained for 1:1 mixtures of **DBTTF-(ViRu)$_2$** and DDQ (red), F_4TCNQ (blue) or TCNQ (green) measured directly after synthesis (left) and after storing for 20 days under nitrogen atmosphere (right).

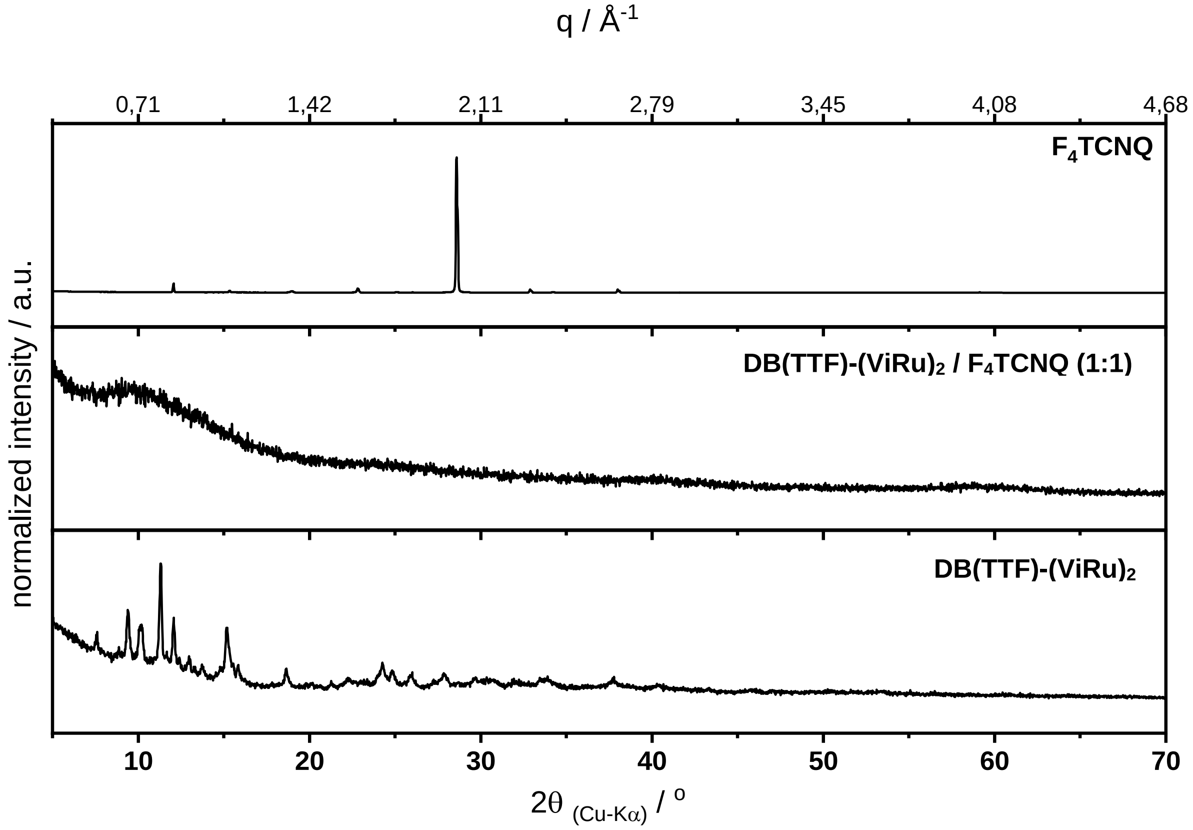

Figure S58. Powder X-ray diffraction spectra obtained for F_4TCNQ (top), a 1:1 mixture of F_4TCNQ and **DBTTF-(ViRu)$_2$** (middle) and **DBTTF-(ViRu)$_2$** (bottom).

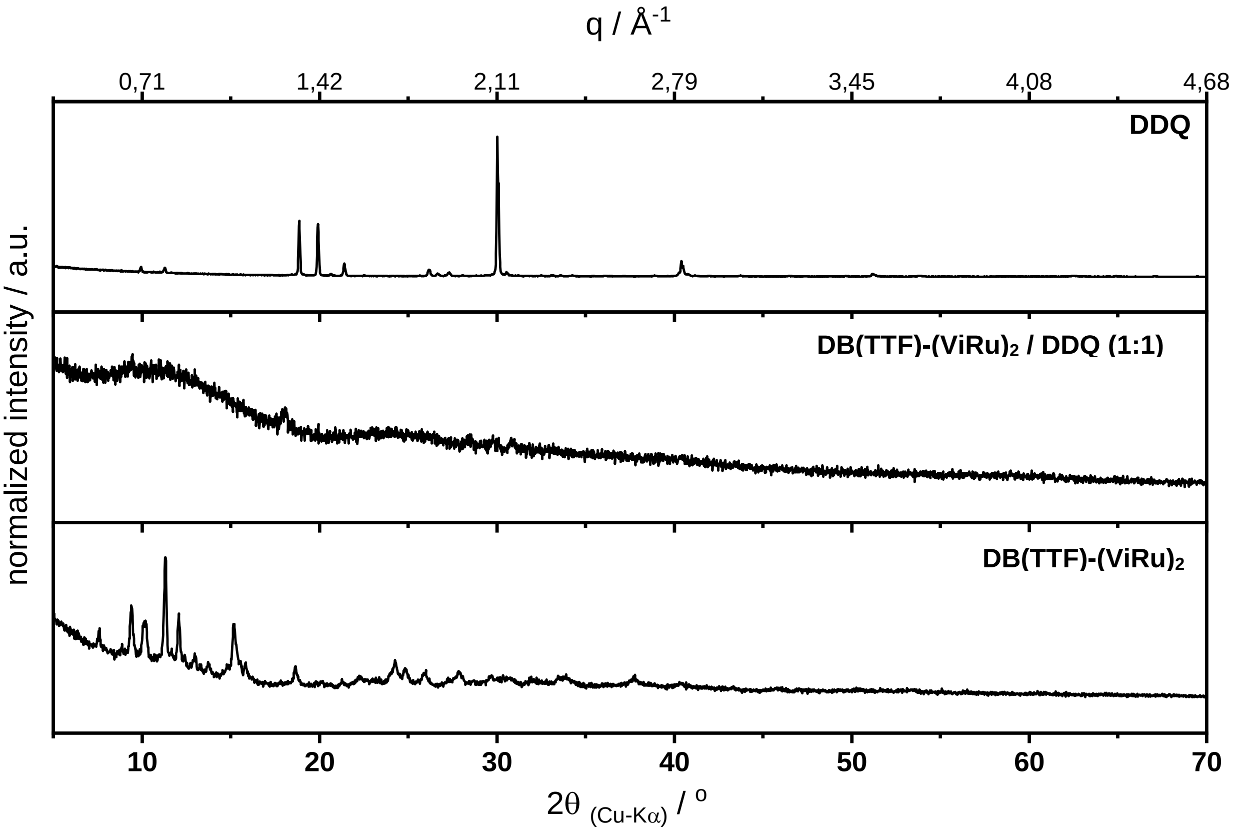

Figure S59. Powder X-ray diffraction spectra obtained for DDQ (top), a 1:1 mixture of DDQ and **DBTTF-(ViRu)$_2$** (middle) and **DBTTF-(ViRu)$_2$** (bottom).

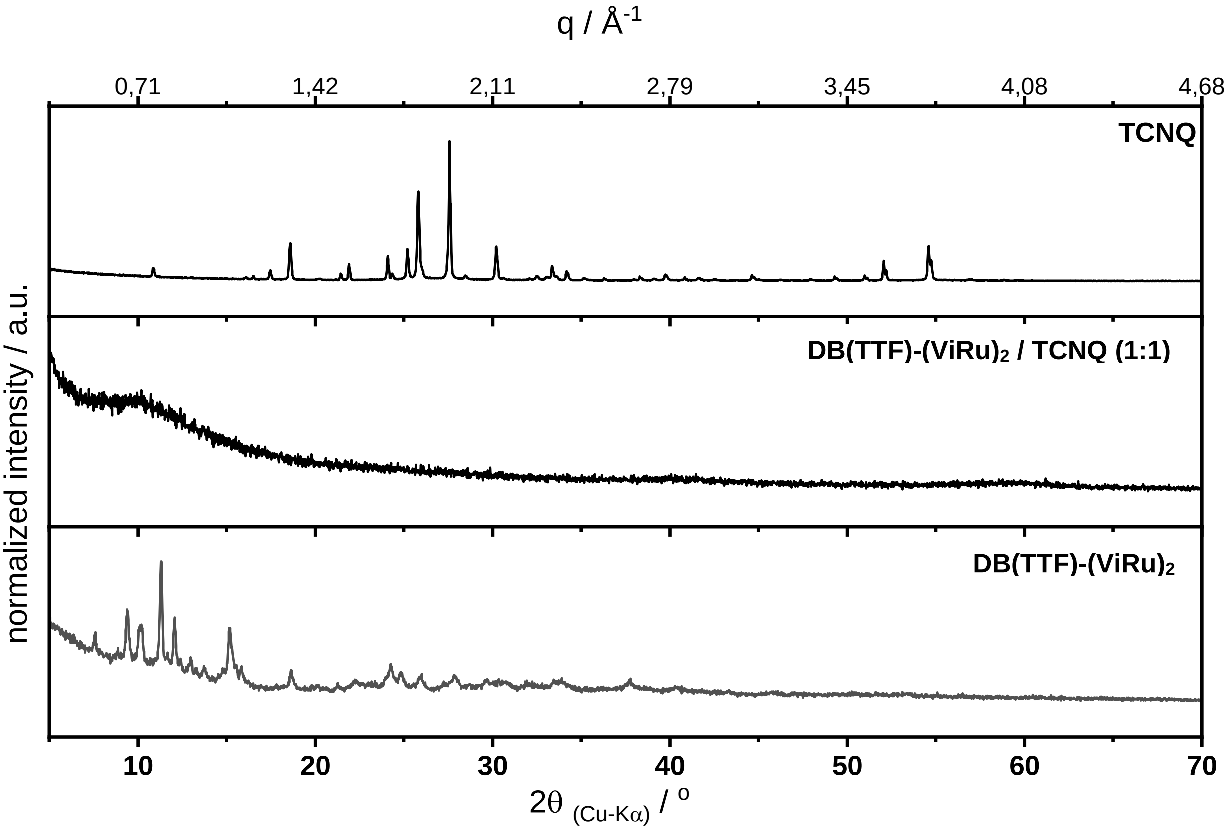

Figure S60. Powder X-ray diffraction spectra obtained for TCNQ (top), a 1:1 mixture of TCNQ and **DBTTF-(ViRu)$_2$** (middle) and **DBTTF-(ViRu)$_2$** (bottom).

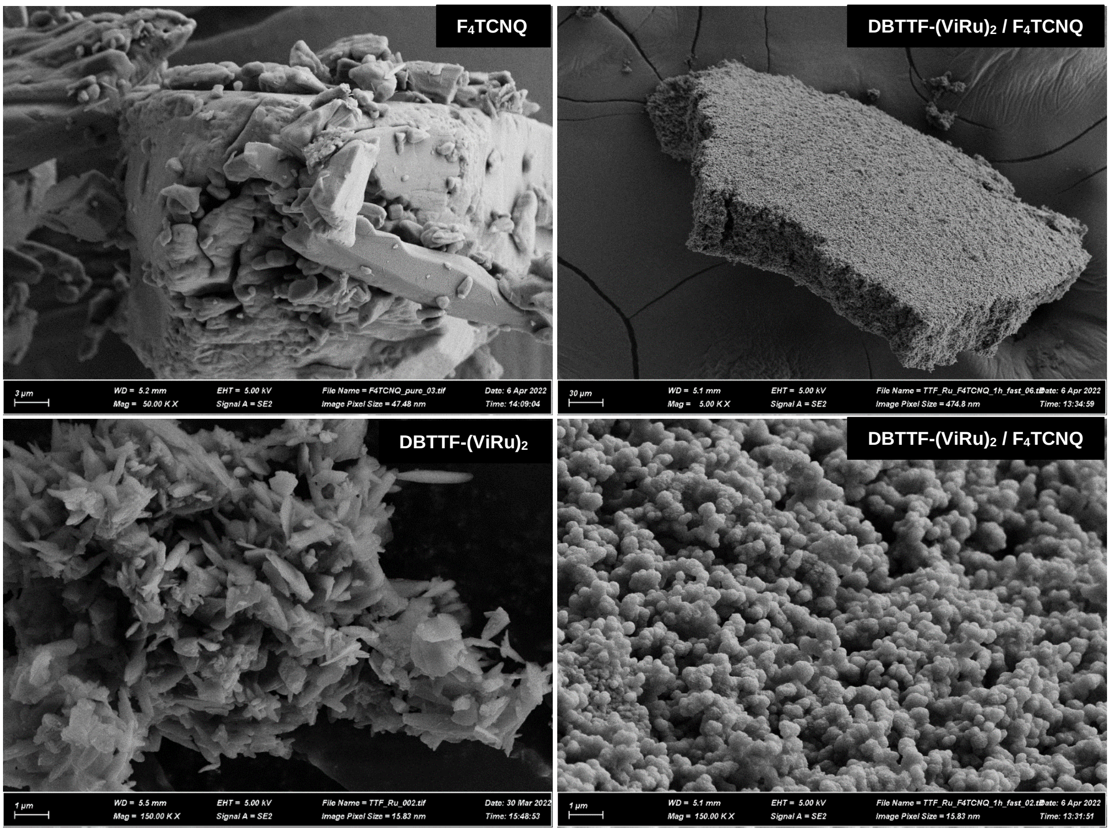

Figure S61. SEM images of F$_4$TCNQ (top left), **DBTTF-(ViRu)$_2$** (bottom left), and a 1:1 mixture of **DBTTF-(ViRu)$_2$** and F$_4$TCNQ (right).

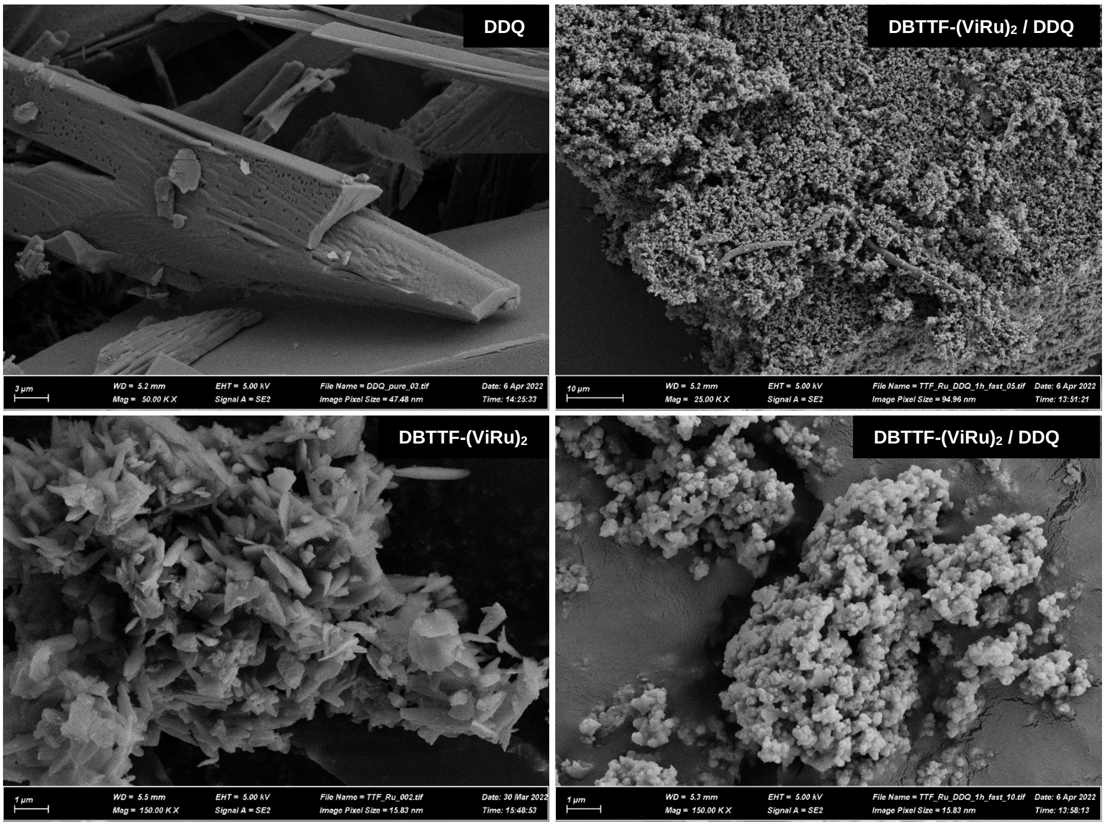

Figure S62. SEM images of DDQ (top left), **DBTTF-(ViRu)$_2$** (bottom left), and a 1:1 mixture of **DBTTF-(ViRu)$_2$** and DDQ (right).

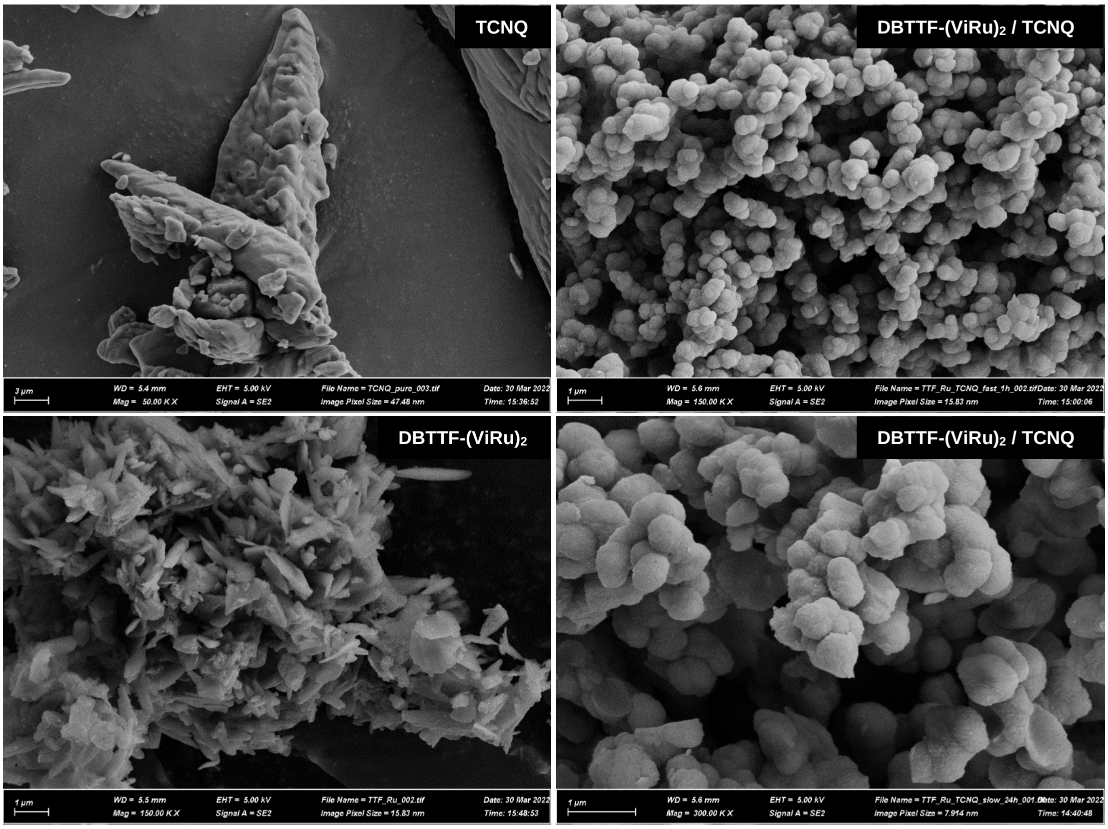

Figure S63. SEM images of TCNQ (top left), **DBTTF-(ViRu)$_2$** (bottom left), and a 1:1 mixture of **DBTTF-(ViRu)$_2$** and TCNQ (right).

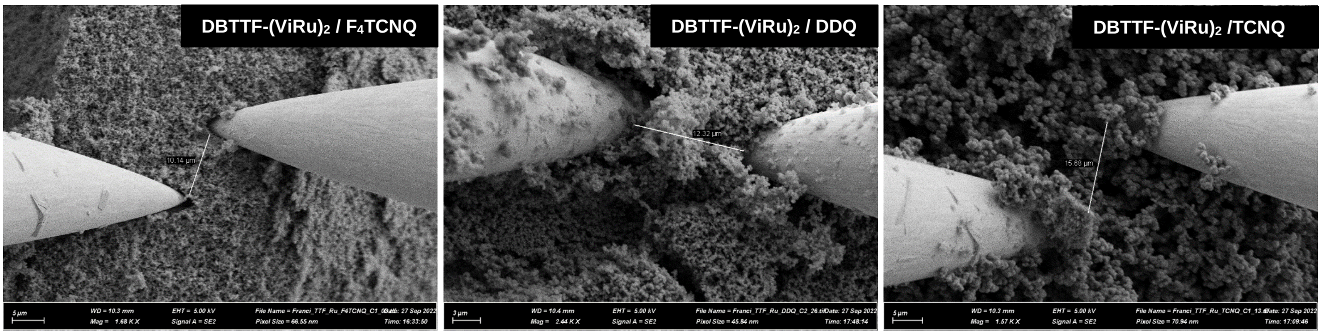

Figure S64. SEM images taken from conductivity measurements on 1:1 mixtures of **DBTTF-(ViRu)$_2$** and F_4TCNQ (left), DDQ (middle), and TCNQ (right).

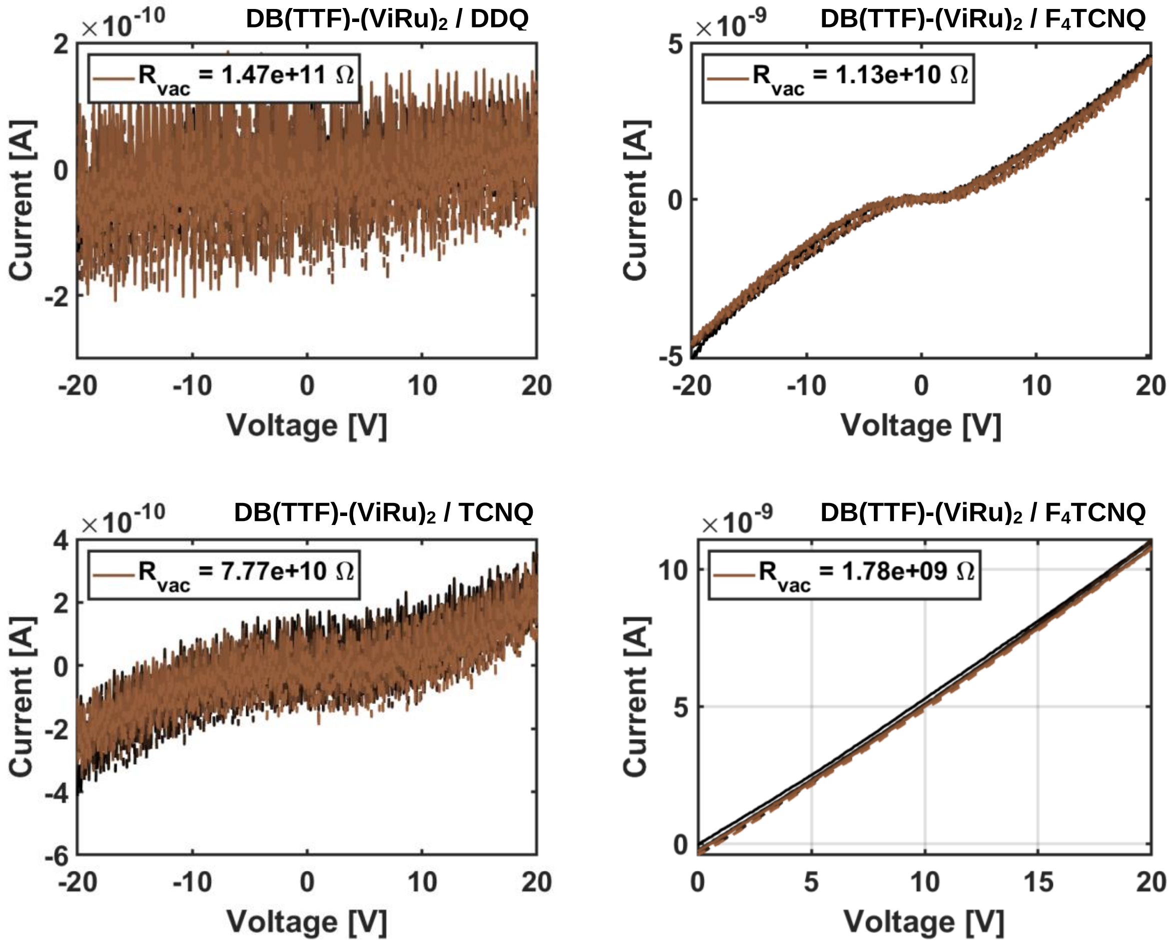

Figure S65. Conductivity measurements on 1:1 mixtures of **DBTTF-(ViRu)$_2$** and DDQ (top left), TCNQ (bottom left) , and F_4TCNQ using nanoprobing (top right) and fixation between gold electrodes (bottom right).

Atomic Coordinates for Geometry-Optimized Structures

Table S24. Atomic coordinates of **DBTTF-(ViRu)$_{2Z}$** from its pbe1pbe calculated DFT structure.

Ru	9.28526	0.35451	-0.05841
Cl	9.0642	0.73847	-2.47632
P	9.68497	-1.99401	-0.51498
S	0.86843	-1.71132	2.02679
S	2.10053	-1.70084	-0.67185
P	9.13341	2.75698	0.23653
O	10.07086	0.18652	2.80347
C	7.40454	-0.04822	0.37979
H	6.85037	-0.04745	-0.56781
C	2.57028	-1.26235	1.94346
C	3.15509	-1.25306	0.67073
C	4.50073	-0.95669	0.50469
H	4.92993	-0.96773	-0.49308
C	3.35397	-0.9746	3.05742
H	2.91668	-0.97634	4.05182
C	4.70208	-0.68235	2.88831
H	5.30603	-0.45636	3.76356
C	10.33171	-3.05481	0.88052
H	10.43438	-4.05836	0.44738
C	9.75405	0.21761	1.67851
C	5.30612	-0.65806	1.61834
C	11.70842	-2.63061	1.39305
H	12.49453	-2.78879	0.65014
H	11.96574	-3.23254	2.27328
H	11.73123	-1.57952	1.69663
C	0.61359	-1.67612	0.28039
C	8.19177	-4.4612	-0.96511
H	8.88604	-4.89375	-1.69159
H	7.19425	-4.85128	-1.20223
H	8.45743	-4.83804	0.02578
C	9.32719	-3.13441	2.03332
H	9.24321	-2.17907	2.55659
H	9.67097	-3.8812	2.75936
H	8.32363	-3.42373	1.70827
C	10.92993	-2.14228	-1.90327
H	10.34524	-1.81909	-2.77409
C	6.73318	-0.33473	1.51386
H	7.25945	-0.31421	2.46864
C	8.14393	-2.9333	-1.04743
H	7.40627	-2.58061	-0.31357
C	12.07037	-1.13204	-1.74314
H	11.69066	-0.1106	-1.63368
H	12.69942	-1.14975	-2.64177
H	12.71293	-1.35532	-0.88663

C	11.31459	3.46506	-1.45494
H	11.30678	2.45167	-1.86921
H	12.342	3.84619	-1.5037
H	10.6997	4.09511	-2.10479
C	10.8411	3.48968	-0.00214
H	10.77011	4.5383	0.31198
C	11.47456	-3.54508	-2.16865
H	12.03805	-3.93796	-1.31518
H	12.16226	-3.50914	-3.02265
H	10.68744	-4.26215	-2.41412
C	8.09227	3.67726	-1.01194
H	8.61548	3.42721	-1.94309
C	7.65799	-2.50349	-2.43167
H	7.59005	-1.41751	-2.53093
H	6.66608	-2.93435	-2.61459
H	8.32396	-2.87262	-3.22075
C	8.67701	3.31288	1.97313
H	9.15423	2.55245	2.60335
C	8.08246	5.19783	-0.85272
H	7.49768	5.51006	0.01877
H	7.6128	5.64756	-1.73624
H	9.08621	5.62544	-0.76198
C	11.85325	2.78496	0.90377
H	11.54602	2.76696	1.95477
H	12.82021	3.29907	0.84795
H	12.0122	1.74703	0.5861
C	7.17005	3.22578	2.22627
H	6.63374	4.03975	1.72726
H	6.98004	3.32358	3.30208
H	6.74832	2.27322	1.89312
C	6.67345	3.12765	-1.15878
H	6.68337	2.05219	-1.34831
H	6.19134	3.61546	-2.01508
H	6.05519	3.32142	-0.27818
C	9.21561	4.67413	2.41991
H	10.30838	4.72129	2.41525
H	8.88918	4.85683	3.45126
H	8.83488	5.49596	1.80677
Ru	-9.28524	0.3545	0.05838
Cl	-9.06421	0.73846	2.4763
P	-9.68498	-1.99401	0.51497
S	-0.86839	-1.71132	-2.02675
S	-2.10049	-1.70084	0.67189
P	-9.1334	2.75697	-0.23654
O	-10.0708	0.18641	-2.8035
C	-7.40451	-0.04825	-0.37978
H	-6.85035	-0.04746	0.56782

C	-2.57024	-1.26238	-1.94342
C	-3.15506	-1.25308	-0.67069
C	-4.5007	-0.95672	-0.50465
H	-4.92991	-0.96777	0.49311
C	-3.35393	-0.97464	-3.05738
H	-2.91664	-0.97639	-4.05178
C	-4.70205	-0.68241	-2.88828
H	-5.306	-0.45643	-3.76354
C	-10.33162	-3.05483	-0.88057
H	-10.43435	-4.05837	-0.44741
C	-9.75402	0.21759	-1.67854
C	-5.30609	-0.6581	-1.61831
C	-11.70828	-2.63062	-1.39324
H	-12.49446	-2.78881	-0.6504
H	-11.96552	-3.23255	-2.27349
H	-11.73106	-1.57953	-1.69681
C	-0.61355	-1.67612	-0.28034
C	-8.19188	-4.46121	0.96539
H	-8.88622	-4.89366	1.69185
H	-7.19439	-4.85129	1.20262
H	-8.45747	-4.83813	-0.02549
C	-9.327	-3.13446	-2.03328
H	-9.24299	-2.17915	-2.55658
H	-9.67071	-3.88129	-2.75932
H	-8.32347	-3.42376	-1.70814
C	-10.93005	-2.1422	1.90317
H	-10.3454	-1.81905	2.77402
C	-6.73315	-0.33478	-1.51384
H	-7.25942	-0.3143	-2.46862
C	-8.144	-2.9333	1.04757
H	-7.40628	-2.5807	0.31373
C	-12.07041	-1.13188	1.74296
H	-11.69062	-0.11047	1.6335
H	-12.69949	-1.14952	2.64156
H	-12.71294	-1.35513	0.88643
C	-11.31486	3.46493	1.45464
H	-11.30709	2.45152	1.86885
H	-12.34228	3.84603	1.50325
H	-10.70009	4.09497	2.10461
C	-10.84114	3.48963	0.00191
H	-10.77012	4.53827	-0.31215
C	-11.47477	-3.54497	2.16851
H	-12.03823	-3.93782	1.31501
H	-12.16252	-3.50899	3.02248
H	-10.6877	-4.26209	2.41402
C	-8.09245	3.67726	1.01208
H	-8.61581	3.42723	1.94315

C	-7.65813	-2.5034	2.4318
H	-7.59014	-1.41741	2.53097
H	-6.66625	-2.93428	2.61483
H	-8.32417	-2.87242	3.22088
C	-8.67682	3.31288	-1.97309
H	-9.1538	2.55234	-2.60335
C	-8.08261	5.19784	0.85285
H	-7.49767	5.51006	-0.01852
H	-7.61312	5.64757	1.73646
H	-9.08635	5.62545	0.76193
C	-11.85313	2.78493	-0.9042
H	-11.54574	2.76701	-1.95515
H	-12.82012	3.29901	-0.8485
H	-12.0121	1.74697	-0.58663
C	-7.1698	3.22604	-2.22597
H	-6.63371	4.04013	-1.72693
H	-6.97963	3.3238	-3.30176
H	-6.74795	2.27358	-1.89269
C	-6.67366	3.12766	1.15916
H	-6.6836	2.0522	1.34868
H	-6.19169	3.61546	2.01554
H	-6.05524	3.32144	0.27866
C	-9.21559	4.67401	-2.42003
H	-10.30837	4.72097	-2.41556
H	-8.88902	4.85671	-3.45133
H	-8.83512	5.49594	-1.80687

Table S25. Atomic coordinates of **DBTTF-(ViRu)$_{2Z}^{+}$** from its pbe1pbe calculated DFT structure.

Ru	9.53702	-0.00043	-0.06489
Cl	9.41226	0.22427	-2.50294
P	9.47105	-2.41267	-0.32712
S	0.90153	-0.00871	1.98439
S	2.07148	-0.00299	-0.7097
P	9.91287	2.3993	0.02601
O	10.23109	-0.09063	2.82635
C	7.62316	0.03183	0.33546
H	7.09247	0.08333	-0.62369
C	2.63808	-0.01542	1.90713
C	3.20825	-0.01215	0.62181
C	4.58362	-0.01874	0.44394
H	4.99323	-0.02192	-0.56076
C	3.46921	-0.02667	3.03042
H	3.04722	-0.0306	4.03059
C	4.83883	-0.03218	2.84687
H	5.48451	-0.0396	3.72065
C	9.85881	-3.46277	1.16763

H	9.75995	-4.49665	0.81223
C	9.94411	-0.08717	1.69494
C	5.43547	-0.02605	1.5623
C	11.2821	-3.29321	1.7
H	12.03438	-3.66653	1.0005
H	11.38741	-3.86758	2.62849
H	11.51799	-2.25018	1.93247
C	0.63641	-0.00539	0.27319
C	7.5331	-4.56886	-0.63377
H	8.17256	-5.19902	-1.25889
H	6.49508	-4.78535	-0.91469
H	7.65758	-4.88269	0.40556
C	8.83533	-3.24312	2.28546
H	8.96215	-2.265	2.7555
H	8.9823	-4.00365	3.06169
H	7.79942	-3.3147	1.94113
C	10.7	-2.89504	-1.65001
H	10.21531	-2.52454	-2.56221
C	6.88865	-0.03	1.47364
H	7.3975	-0.0786	2.4357
C	7.79108	-3.07641	-0.85819
H	7.10946	-2.51589	-0.20364
C	12.01125	-2.11651	-1.50441
H	11.83759	-1.03495	-1.47651
H	12.65034	-2.31745	-2.3729
H	12.57242	-2.39856	-0.60921
C	12.19388	2.47876	-1.6824
H	11.97092	1.46176	-2.02046
H	13.27873	2.62791	-1.7425
H	11.72774	3.17404	-2.38703
C	11.73841	2.71539	-0.24302
H	11.89795	3.77509	-0.00904
C	10.96093	-4.39253	-1.80479
H	11.40395	-4.83135	-0.90416
H	11.67024	-4.55117	-2.62632
H	10.05395	-4.95298	-2.04485
C	9.0914	3.41325	-1.31008
H	9.54611	2.98063	-2.20967
C	7.45639	-2.70391	-2.30268
H	7.61071	-1.64158	-2.50572
H	6.40649	-2.94988	-2.50253
H	8.06548	-3.27537	-3.01299
C	9.59193	3.17723	1.70576
H	9.90514	2.38965	2.40178
C	9.41149	4.90796	-1.27296
H	8.90622	5.4099	-0.44132
H	9.0525	5.37321	-2.1991

H	10.4838	5.1147	-1.19709
C	12.57141	1.87853	0.73057
H	12.27063	2.01262	1.77504
H	13.62761	2.16122	0.64965
H	12.49857	0.8089	0.49715
C	8.10069	3.42288	1.94599
H	7.74149	4.286	1.37594
H	7.93919	3.64456	3.00776
H	7.48988	2.5535	1.68559
C	7.58701	3.17328	-1.43378
H	7.36395	2.10975	-1.54196
H	7.21972	3.68586	-2.33142
H	7.0279	3.56365	-0.57915
C	10.40402	4.4292	2.04573
H	11.48218	4.24545	2.05563
H	10.12474	4.7622	3.05293
H	10.20382	5.25756	1.36042
Ru	-9.53701	-0.0004	0.06487
Cl	-9.41225	0.22428	2.50292
P	-9.47111	-2.41264	0.32709
S	-0.90151	-0.00859	-1.98432
S	-2.07147	-0.00321	0.70977
P	-9.91282	2.39933	-0.026
O	-10.23108	-0.09061	-2.82637
C	-7.62314	0.03181	-0.33545
H	-7.09246	0.08341	0.6237
C	-2.63805	-0.01538	-1.90707
C	-3.20823	-0.01227	-0.62176
C	-4.5836	-0.01895	-0.4439
H	-4.99322	-0.02228	0.5608
C	-3.46917	-0.02655	-3.03037
H	-3.04717	-0.03036	-4.03054
C	-4.83879	-0.03213	-2.84683
H	-5.48446	-0.03947	-3.72062
C	-9.85894	-3.46273	-1.16765
H	-9.76007	-4.4966	-0.81225
C	-9.94408	-0.08712	-1.69497
C	-5.43544	-0.02615	-1.56227
C	-11.28224	-3.29316	-1.69997
H	-12.03451	-3.66643	-1.00044
H	-11.38759	-3.86756	-2.62844
H	-11.5181	-2.25013	-1.93247
C	-0.63639	-0.00541	-0.27312
C	-7.53315	-4.56886	0.63349
H	-8.17267	-5.19911	1.25846
H	-6.49515	-4.7854	0.91445
H	-7.65755	-4.88254	-0.40591

C	-8.83548	-3.24309	-2.28551
H	-8.96237	-2.26501	-2.75559
H	-8.98243	-4.00367	-3.0617
H	-7.79956	-3.3146	-1.9412
C	-10.70003	-2.89498	1.65002
H	-10.21534	-2.52442	2.5622
C	-6.88863	-0.03013	-1.47362
H	-7.39746	-0.07881	-2.43569
C	-7.79113	-3.07644	0.85809
H	-7.1095	-2.51585	0.20362
C	-12.0113	-2.11648	1.50438
H	-11.83764	-1.03493	1.47647
H	-12.65041	-2.31744	2.37286
H	-12.57244	-2.39856	0.60917
C	-12.19408	2.47857	1.68209
H	-11.9712	1.46151	2.02001
H	-13.27894	2.62772	1.74204
H	-11.72805	3.17372	2.38689
C	-11.73839	2.71541	0.24281
H	-11.89788	3.77516	0.00896
C	-10.9609	-4.39247	1.80487
H	-11.4039	-4.83135	0.90426
H	-11.67021	-4.5511	2.6264
H	-10.0539	-4.95288	2.04495
C	-9.09152	3.41321	1.31024
H	-9.54644	2.98062	2.20975
C	-7.45644	-2.70411	2.30263
H	-7.61081	-1.64181	2.5058
H	-6.40653	-2.95006	2.50244
H	-8.0655	-3.2757	3.01286
C	-9.59168	3.17733	-1.70568
H	-9.90457	2.38968	-2.40177
C	-9.41147	4.90794	1.27308
H	-8.90596	5.40985	0.44156
H	-9.05264	5.37315	2.1993
H	-10.48374	5.11479	1.19696
C	-12.57126	1.87873	-0.73104
H	-12.27033	2.01299	-1.77544
H	-13.62748	2.16141	-0.65021
H	-12.49846	0.80906	-0.49778
C	-8.10044	3.42331	-1.94561
H	-7.74156	4.28653	-1.3755
H	-7.93877	3.64499	-3.00735
H	-7.48949	2.55408	-1.68505
C	-7.58717	3.17309	1.43425
H	-7.36423	2.10953	1.54244
H	-7.22002	3.68562	2.33197

H	-7.02785	3.56342	0.57974
C	-10.40398	4.42911	-2.04584
H	-11.48209	4.24512	-2.05595
H	-10.12457	4.76215	-3.05299
H	-10.20409	5.25753	-1.3605

Table S26. Atomic coordinates of **DBTTF-(ViRu)$_{2Z}^{2+}$ (S)** from its pbe1pbe calculated DFT structure.

Ru	9.50419	0.0148	-0.05321
Cl	9.35253	0.26424	-2.47413
P	9.51272	-2.40422	-0.35162
S	0.92987	-0.07389	1.95654
S	2.06807	-0.05087	-0.72422
P	9.91421	2.41799	0.04584
O	10.18982	-0.09947	2.84497
C	7.61837	0.02674	0.31747
H	7.08566	0.07771	-0.63963
C	2.64269	-0.07272	1.88529
C	3.21684	-0.05814	0.59019
C	4.58616	-0.0518	0.41271
H	4.99868	-0.04382	-0.58989
C	3.47212	-0.08729	3.01741
H	3.04732	-0.10106	4.01573
C	4.83409	-0.08295	2.83168
H	5.48225	-0.09341	3.70292
C	9.93128	-3.45346	1.13372
H	9.86984	-4.48464	0.76204
C	9.90719	-0.08662	1.71636
C	5.43597	-0.06003	1.53876
C	11.34614	-3.2436	1.67404
H	12.11304	-3.57603	0.97006
H	11.46782	-3.83372	2.59022
H	11.5459	-2.19872	1.93036
C	0.64833	-0.06205	0.26626
C	7.64711	-4.60783	-0.70092
H	8.30092	-5.19805	-1.34934
H	6.61434	-4.85245	-0.97723
H	7.79348	-4.94322	0.32862
C	8.8937	-3.28719	2.24761
H	8.97781	-2.31152	2.73235
H	9.06776	-4.05012	3.01537
H	7.86352	-3.39924	1.897
C	10.76019	-2.81208	-1.67891
H	10.26216	-2.44966	-2.58708
C	6.8711	-0.04704	1.46671
H	7.37998	-0.09086	2.42788
C	7.84936	-3.102	-0.89157

H	7.15319	-2.58157	-0.21924
C	12.03725	-1.98226	-1.51497
H	11.82032	-0.90862	-1.47795
H	12.68866	-2.14606	-2.38169
H	12.60415	-2.25105	-0.6195
C	12.17305	2.48259	-1.69215
H	11.94277	1.47105	-2.04081
H	13.25747	2.62783	-1.763
H	11.70194	3.1892	-2.38178
C	11.73896	2.7046	-0.24394
H	11.91236	3.75955	0.00154
C	11.082	-4.29596	-1.85022
H	11.55023	-4.72403	-0.95754
H	11.79153	-4.41392	-2.67799
H	10.19866	-4.89241	-2.09089
C	9.07775	3.43893	-1.27354
H	9.5161	3.00918	-2.18237
C	7.4938	-2.70933	-2.32543
H	7.59941	-1.63708	-2.50481
H	6.45515	-2.9975	-2.52643
H	8.12402	-3.23675	-3.0507
C	9.6174	3.17361	1.73847
H	9.94612	2.38209	2.42229
C	9.41279	4.93059	-1.22789
H	8.91972	5.43132	-0.38844
H	9.04865	5.40366	-2.14768
H	10.48747	5.12738	-1.16218
C	12.57108	1.84681	0.7121
H	12.28776	1.97869	1.76164
H	13.62966	2.11502	0.6184
H	12.48225	0.77987	0.47161
C	8.12972	3.41183	2.00489
H	7.75957	4.28173	1.45286
H	7.98491	3.6172	3.07194
H	7.51403	2.54594	1.74271
C	7.56943	3.2143	-1.37403
H	7.33223	2.15455	-1.48988
H	7.19446	3.73856	-2.26133
H	7.02801	3.60369	-0.5078
C	10.43325	4.42474	2.07337
H	11.51145	4.24202	2.06261
H	10.17137	4.74721	3.08833
H	10.21917	5.25856	1.39945
Ru	-9.50419	0.01479	0.05321
Cl	-9.35253	0.26419	2.47413
P	-9.51269	-2.40423	0.35159
S	-0.92987	-0.07374	-1.95655

S	-2.06807	-0.05094	0.72421
P	-9.91423	2.41797	-0.0458
O	-10.18984	-0.09941	-2.84496
C	-7.61837	0.02675	-0.31748
H	-7.08565	0.07768	0.63962
C	-2.64269	-0.07259	-1.88531
C	-3.21685	-0.05811	-0.5902
C	-4.58616	-0.0518	-0.41272
H	-4.99868	-0.04391	0.58988
C	-3.47212	-0.08708	-3.01742
H	-3.04732	-0.10077	-4.01575
C	-4.83409	-0.08277	-2.83169
H	-5.48225	-0.09317	-3.70294
C	-9.93119	-3.45348	-1.13377
H	-9.86973	-4.48466	-0.76209
C	-9.90719	-0.0866	-1.71636
C	-5.43597	-0.05996	-1.53877
C	-11.34604	-3.24365	-1.67413
H	-12.11295	-3.57612	-0.97018
H	-11.46767	-3.83376	-2.59032
H	-11.54582	-2.19877	-1.93043
C	-0.64833	-0.06203	-0.26627
C	-7.64706	-4.60782	0.70093
H	-8.3009	-5.19806	1.3493
H	-6.6143	-4.85244	0.9773
H	-7.79337	-4.9432	-0.32862
C	-8.89358	-3.28718	-2.24763
H	-8.97772	-2.31152	-2.73239
H	-9.06758	-4.05013	-3.01538
H	-7.8634	-3.39917	-1.89699
C	-10.76018	-2.81212	1.67884
H	-10.26218	-2.44971	2.58703
C	-6.8711	-0.04698	-1.46672
H	-7.37998	-0.09076	-2.4279
C	-7.84933	-3.102	0.89159
H	-7.15315	-2.58155	0.2193
C	-12.03724	-1.9823	1.51489
H	-11.82032	-0.90866	1.47788
H	-12.68867	-2.14611	2.38159
H	-12.60414	-2.25109	0.61941
C	-12.17311	2.48251	1.69215
H	-11.94283	1.47095	2.04078
H	-13.25753	2.62773	1.76298
H	-11.70201	3.1891	2.38181
C	-11.73899	2.70455	0.24396
H	-11.9124	3.75951	-0.0015
C	-11.08199	-4.296	1.85013

H	-11.55019	-4.72407	0.95743
H	-11.79155	-4.41398	2.67787
H	-10.19865	-4.89245	2.09082
C	-9.07781	3.4389	1.27361
H	-9.51616	3.00912	2.18243
C	-7.49384	-2.70933	2.32547
H	-7.59948	-1.63708	2.50485
H	-6.45519	-2.99748	2.52651
H	-8.12408	-3.23677	3.05071
C	-9.61741	3.17363	-1.73841
H	-9.9461	2.38212	-2.42226
C	-9.41288	4.93055	1.228
H	-8.9198	5.43132	0.38856
H	-9.04876	5.40361	2.14779
H	-10.48756	5.12733	1.16227
C	-12.57109	1.84678	-0.71213
H	-12.28776	1.9787	-1.76165
H	-13.62967	2.11498	-0.61844
H	-12.48224	0.77984	-0.47167
C	-8.12973	3.41189	-2.0048
H	-7.75961	4.28179	-1.45275
H	-7.9849	3.61728	-3.07184
H	-7.51403	2.54601	-1.74263
C	-7.56948	3.2143	1.37411
H	-7.33226	2.15455	1.48995
H	-7.19454	3.73855	2.26143
H	-7.02806	3.60371	0.5079
C	-10.43328	4.42475	-2.0733
H	-11.51148	4.242	-2.06257
H	-10.17139	4.74724	-3.08826
H	-10.21924	5.25857	-1.39937

Table S27. Atomic coordinates of **DBTTF-(ViRu)$_{2Z}^{2+}$ (T)** from its pbe1pbe calculated DFT structure.

Ru	9.47888	0.05975	-0.04223
Cl	9.33883	0.21221	-2.4581
P	9.65838	-2.37334	-0.26532
S	0.92013	-0.2897	1.98758
S	2.07403	-0.28407	-0.74008
P	9.83419	2.48419	-0.0413
O	10.15448	0.08443	2.86678
C	7.60993	0.00271	0.29481
H	7.07851	-0.00667	-0.66316
C	2.64905	-0.23017	1.88785
C	3.21179	-0.21897	0.59265
C	4.57969	-0.16456	0.41766
H	4.99098	-0.15169	-0.58573

C	3.4785	-0.1961	3.01487
H	3.05201	-0.20675	4.01269
C	4.84533	-0.14621	2.83723
H	5.49308	-0.11879	3.70834
C	10.11224	-3.33321	1.26709
H	10.11683	-4.37867	0.9321
C	9.87372	0.04196	1.74192
C	5.43244	-0.12411	1.54513
C	11.50357	-3.02056	1.81791
H	12.29934	-3.33823	1.13988
H	11.64197	-3.5646	2.7596
H	11.64063	-1.95669	2.03351
C	0.62925	-0.28667	0.25908
C	7.94254	-4.69273	-0.55542
H	8.65932	-5.2687	-1.14736
H	6.94008	-5.01756	-0.85832
H	8.06654	-4.96655	0.49486
C	9.04664	-3.18808	2.35747
H	9.0774	-2.19866	2.8202
H	9.24239	-3.92254	3.14704
H	8.02887	-3.35755	1.9937
C	10.95645	-2.72604	-1.55693
H	10.45299	-2.4222	-2.48335
C	6.86256	-0.05718	1.45876
H	7.38173	-0.04569	2.41521
C	8.05183	-3.1874	-0.81648
H	7.30315	-2.68182	-0.19127
C	12.17272	-1.81044	-1.39158
H	11.88736	-0.75223	-1.39648
H	12.85294	-1.95862	-2.23844
H	12.73452	-2.01278	-0.47589
C	12.09345	2.54325	-1.77832
H	11.88991	1.51739	-2.09995
H	13.17399	2.71317	-1.84977
H	11.60772	3.21941	-2.48786
C	11.65146	2.79675	-0.33779
H	11.79651	3.86302	-0.12494
C	11.37549	-4.19064	-1.67811
H	11.85875	-4.56031	-0.76763
H	12.10249	-4.28453	-2.49343
H	10.53656	-4.8504	-1.91234
C	8.965	3.41571	-1.40289
H	9.41952	2.96563	-2.29361
C	7.72061	-2.88994	-2.27886
H	7.75801	-1.82337	-2.51007
H	6.71094	-3.25831	-2.49415
H	8.40766	-3.40902	-2.95677

C	9.50648	3.28635	1.6228
H	9.86343	2.5347	2.33632
C	9.25414	4.91819	-1.41096
H	8.74044	5.43441	-0.59377
H	8.88005	5.34286	-2.34986
H	10.32161	5.15164	-1.34904
C	12.49897	1.99097	0.64972
H	12.21004	2.15313	1.69349
H	13.55093	2.28086	0.54903
H	12.43814	0.91439	0.44609
C	8.01169	3.48602	1.87997
H	7.61447	4.32235	1.29636
H	7.86008	3.72501	2.93865
H	7.42065	2.59382	1.65104
C	7.46448	3.14065	-1.49311
H	7.25825	2.0713	-1.57467
H	7.0748	3.62281	-2.39743
H	6.91099	3.54295	-0.64075
C	10.2811	4.57636	1.90626
H	11.36462	4.42886	1.90152
H	10.00851	4.92761	2.90848
H	10.03857	5.37606	1.20168
Ru	-9.47889	0.05971	0.04224
Cl	-9.33881	0.21209	2.45812
P	-9.65818	-2.37342	0.26522
S	-0.92015	-0.28927	-1.98763
S	-2.07406	-0.28418	0.74004
P	-9.83436	2.48413	0.04142
O	-10.15452	0.08444	-2.86676
C	-7.60994	0.00281	-0.2948
H	-7.07851	-0.00666	0.66316
C	-2.64907	-0.22975	-1.88789
C	-3.21182	-0.21882	-0.59268
C	-4.57972	-0.16444	-0.41769
H	-4.991	-0.15179	0.58571
C	-3.47852	-0.19545	-3.0149
H	-3.05203	-0.20589	-4.01272
C	-4.84535	-0.1456	-2.83726
H	-5.4931	-0.118	-3.70836
C	-10.11226	-3.33321	-1.26718
H	-10.11678	-4.37868	-0.93226
C	-9.87378	0.04195	-1.7419
C	-5.43246	-0.12377	-1.54514
C	-11.50368	-3.02051	-1.81773
H	-12.29934	-3.33838	-1.13965
H	-11.6422	-3.56432	-2.75953
H	-11.64085	-1.95659	-2.03306

C	-0.62928	-0.2866	-0.25912
C	-7.94212	-4.69269	0.55485
H	-8.65872	-5.26875	1.14694
H	-6.93956	-5.01746	0.85749
H	-8.06635	-4.96646	-0.49541
C	-9.04684	-3.18798	-2.35773
H	-9.07774	-2.19856	-2.82044
H	-9.24268	-3.92245	-3.14727
H	-8.02899	-3.35739	-1.99413
C	-10.95601	-2.72631	1.55703
H	-10.45248	-2.42236	2.48337
C	-6.86258	-0.0569	-1.45877
H	-7.38176	-0.04531	-2.41521
C	-8.05148	-3.18739	0.81605
H	-7.30293	-2.68171	0.19076
C	-12.17246	-1.81094	1.39181
H	-11.88729	-0.75267	1.39664
H	-12.85255	-1.95921	2.23876
H	-12.73434	-2.0134	0.4762
C	-12.09364	2.54294	1.77843
H	-11.89003	1.51708	2.10002
H	-13.1742	2.71278	1.84987
H	-11.60797	3.21912	2.48799
C	-11.65165	2.79653	0.33791
H	-11.7968	3.8628	0.1251
C	-11.37476	-4.19099	1.67832
H	-11.85807	-4.56079	0.76792
H	-12.10161	-4.28499	2.49375
H	-10.53566	-4.85058	1.91244
C	-8.96525	3.41566	1.40304
H	-9.41969	2.96548	2.29374
C	-7.72004	-2.89004	2.27839
H	-7.75751	-1.82349	2.50972
H	-6.7103	-3.25832	2.49349
H	-8.40693	-3.40925	2.95637
C	-9.5067	3.28637	-1.62265
H	-9.86363	2.53474	-2.3362
C	-9.25457	4.9181	1.41119
H	-8.74096	5.43443	0.59402
H	-8.88051	5.34277	2.3501
H	-10.32207	5.15142	1.34931
C	-12.4991	1.99072	-0.64964
H	-12.21019	2.15297	-1.6934
H	-13.55108	2.2805	-0.54893
H	-12.43816	0.91413	-0.44607
C	-8.01192	3.4861	-1.87981
H	-7.61471	4.3224	-1.29615

H	-7.86031	3.72515	-2.93848
H	-7.42084	2.5939	-1.65094
C	-7.46469	3.14079	1.4932
H	-7.25833	2.07146	1.57471
H	-7.07504	3.62295	2.39753
H	-6.91127	3.54318	0.64084
C	-10.28137	4.57636	-1.90605
H	-11.36488	4.42881	-1.90132
H	-10.00879	4.92767	-2.90825
H	-10.03888	5.37604	-1.20143

Table S28. Atomic coordinates of **DBTTF-(ViRu)$_{2Z}^{2+}$ (OSS)** from its pbe1pbe calculated DFT structure.

Ru	9.48381	0.054	-0.04346
Cl	9.3383	0.21649	-2.46134
P	9.64173	-2.37736	-0.27307
S	0.92524	-0.2583	1.97895
S	2.07327	-0.25067	-0.73978
P	9.83724	2.47657	-0.03211
O	10.16123	0.06556	2.86394
C	7.61271	0.00246	0.29906
H	7.0808	0.00041	-0.65895
C	2.64897	-0.21301	1.8852
C	3.21473	-0.20055	0.58909
C	4.58197	-0.15375	0.41432
H	4.99369	-0.13969	-0.58878
C	3.47853	-0.18862	3.01388
H	3.05206	-0.20052	4.01163
C	4.84401	-0.14624	2.83483
H	5.49198	-0.12593	3.70598
C	10.09554	-3.34696	1.2536
H	10.09275	-4.39139	0.91539
C	9.88041	0.02826	1.73824
C	5.43477	-0.12198	1.54212
C	11.49058	-3.04419	1.80062
H	12.28214	-3.36515	1.11915
H	11.62904	-3.59098	2.74076
H	11.63472	-1.98153	2.01769
C	0.63297	-0.25657	0.25936
C	7.90941	-4.68635	-0.56538
H	8.61909	-5.26489	-1.16341
H	6.90314	-5.00298	-0.8645
H	8.03703	-4.96575	0.48305
C	9.03502	-3.19876	2.3485
H	9.07272	-2.21038	2.81293
H	9.22958	-3.9361	3.13575

H	8.0151	-3.36172	1.98779
C	10.93252	-2.73697	-1.57048
H	10.42797	-2.42681	-2.49423
C	6.86429	-0.06109	1.4604
H	7.38121	-0.05683	2.41812
C	8.02871	-3.1808	-0.82019
H	7.28616	-2.67271	-0.18961
C	12.15572	-1.83012	-1.40722
H	11.87751	-0.76996	-1.40544
H	12.8309	-1.97885	-2.25808
H	12.72063	-2.04064	-0.49526
C	12.09409	2.54164	-1.77227
H	11.88835	1.51707	-2.09676
H	13.17479	2.71025	-1.8451
H	11.60806	3.22067	-2.47891
C	11.65442	2.79075	-0.33024
H	11.8013	3.85602	-0.11372
C	11.34149	-4.20382	-1.69789
H	11.82512	-4.57959	-0.79008
H	12.06517	-4.30047	-2.51588
H	10.4973	-4.85727	-1.93103
C	8.96864	3.41726	-1.38806
H	9.4211	2.97014	-2.28135
C	7.6932	-2.87427	-2.2797
H	7.7401	-1.80706	-2.50643
H	6.67922	-3.23229	-2.49246
H	8.3726	-3.3966	-2.96286
C	9.51413	3.27425	1.6352
H	9.87052	2.51896	2.34524
C	9.25963	4.91928	-1.39097
H	8.74831	5.43296	-0.57065
H	8.88452	5.3486	-2.32741
H	10.32757	5.15087	-1.32988
C	12.50326	1.98017	0.65218
H	12.21604	2.1377	1.69714
H	13.55536	2.26961	0.55108
H	12.44086	0.90454	0.44384
C	8.01995	3.47492	1.89532
H	7.6229	4.31364	1.31493
H	7.86994	3.71084	2.95499
H	7.42809	2.58383	1.66419
C	7.46767	3.14452	-1.47795
H	7.26055	2.07557	-1.5624
H	7.07736	3.63001	-2.38028
H	6.91554	3.54452	-0.62358
C	10.29086	4.56184	1.92325
H	11.37418	4.4128	1.91645

H	10.02015	4.90973	2.92721
H	10.04882	5.36472	1.22206
Ru	-9.48383	0.05399	0.04347
Cl	-9.33833	0.2165	2.46136
P	-9.64165	-2.37738	0.27307
S	-0.92526	-0.25825	-1.97894
S	-2.07328	-0.25065	0.73979
P	-9.83732	2.47655	0.03212
O	-10.16124	0.06549	-2.86393
C	-7.61273	0.00249	-0.29905
H	-7.08082	0.00042	0.65897
C	-2.64899	-0.21296	-1.88519
C	-3.21474	-0.20051	-0.58908
C	-4.58199	-0.1537	-0.4143
H	-4.99371	-0.13965	0.5888
C	-3.47855	-0.18855	-3.01386
H	-3.05208	-0.20045	-4.01162
C	-4.84403	-0.14616	-2.83481
H	-5.49199	-0.12585	-3.70597
C	-10.09546	-3.347	-1.25359
H	-10.09261	-4.39142	-0.91538
C	-9.88042	0.02823	-1.73823
C	-5.43479	-0.12191	-1.54211
C	-11.49053	-3.04429	-1.80058
H	-12.28206	-3.36519	-1.11905
H	-11.62901	-3.59117	-2.74066
H	-11.63468	-1.98165	-2.01776
C	-0.63299	-0.25656	-0.25935
C	-7.90921	-4.6863	0.56534
H	-8.61875	-5.26486	1.16351
H	-6.90287	-5.00285	0.86431
H	-8.03698	-4.96575	-0.48306
C	-9.03496	-3.19874	-2.34851
H	-9.07269	-2.21036	-2.81291
H	-9.22952	-3.93606	-3.13578
H	-8.01503	-3.36169	-1.98781
C	-10.93241	-2.73708	1.57049
H	-10.42788	-2.42691	2.49425
C	-6.86431	-0.06102	-1.46039
H	-7.38122	-0.05672	-2.41811
C	-8.02858	-3.18074	0.82013
H	-7.28608	-2.67263	0.18949
C	-12.15566	-1.83029	1.40723
H	-11.87751	-0.77011	1.40563
H	-12.83092	-1.97918	2.258
H	-12.72046	-2.04072	0.49518
C	-12.09413	2.54162	1.77234

H	-11.88835	1.51706	2.09684
H	-13.17483	2.7102	1.8452
H	-11.6081	3.22067	2.47895
C	-11.6545	2.79071	0.33029
H	-11.80141	3.85597	0.11376
C	-11.34131	-4.20395	1.69787
H	-11.82505	-4.57967	0.7901
H	-12.06488	-4.30067	2.51595
H	-10.49708	-4.85739	1.93085
C	-8.96869	3.41729	1.38802
H	-9.42108	2.97013	2.28133
C	-7.69299	-2.87417	2.27961
H	-7.73992	-1.80696	2.50633
H	-6.67899	-3.23216	2.49231
H	-8.37232	-3.39653	2.96281
C	-9.51426	3.27421	-1.63521
H	-9.87077	2.51896	-2.34523
C	-9.25978	4.91929	1.39094
H	-8.74855	5.43299	0.57057
H	-8.88462	5.34863	2.32734
H	-10.32773	5.1508	1.32993
C	-12.50335	1.98009	-0.65209
H	-12.21616	2.13762	-1.69706
H	-13.55545	2.26951	-0.55097
H	-12.44092	0.90446	-0.44373
C	-8.02007	3.47472	-1.89543
H	-7.62289	4.31338	-1.31504
H	-7.87012	3.71066	-2.95511
H	-7.4283	2.58356	-1.66437
C	-7.46771	3.14463	1.47781
H	-7.26052	2.07569	1.56227
H	-7.07736	3.63016	2.3801
H	-6.91566	3.54464	0.62339
C	-10.29087	4.56189	-1.9232
H	-11.37421	4.41296	-1.91634
H	-10.02019	4.90976	-2.92716
H	-10.0487	5.36473	-1.22201

Table S29. Atomic coordinates of **DBTTF-(ViRu)$_{2E}$** from its pbe1pbe calculated DFT structure.

Ru	-8.47134	-0.70226	-0.50669
Cl	-7.46379	-1.68616	-2.5212
P	-9.07783	1.24562	-1.81482
S	-1.61857	3.93155	2.84409
S	-1.61734	2.26341	0.39335
P	-8.03637	-2.80801	0.61708
O	-10.13482	0.05154	1.83831

C	-6.91275	0.26432	0.2208
H	-6.09138	0.12505	-0.49411
C	-3.11809	3.08274	2.47349
C	-3.12276	2.29437	1.31541
C	-4.26518	1.61484	0.91712
H	-4.23414	1.00103	0.02168
C	-4.27876	3.18458	3.23456
H	-4.29145	3.78501	4.13973
C	-5.42332	2.50847	2.82939
H	-6.32515	2.59277	3.43068
C	-10.26674	2.47302	-1.05864
H	-10.3753	3.25987	-1.8164
C	-9.47788	-0.20999	0.90723
C	-5.45014	1.71382	1.66899
C	-11.65909	1.90544	-0.78135
H	-12.19471	1.65053	-1.69949
H	-12.25577	2.66074	-0.25506
H	-11.62457	1.01507	-0.14603
C	-0.65945	3.07635	1.63403
C	-7.92386	3.77712	-2.69604
H	-8.46784	3.82298	-3.64428
H	-6.97358	4.30476	-2.84433
H	-8.49125	4.34003	-1.9507
C	-9.6838	3.10758	0.20722
H	-9.65733	2.3935	1.03362
H	-10.31931	3.94689	0.51493
H	-8.66874	3.49078	0.06822
C	-9.85736	0.67893	-3.41734
H	-8.99665	0.26454	-3.95754
C	-6.69717	1.0308	1.30954
H	-7.50902	1.18674	2.02036
C	-7.61477	2.3389	-2.27176
H	-7.06199	2.37974	-1.32332
C	-10.82916	-0.48288	-3.18893
H	-10.35224	-1.30496	-2.64428
H	-11.15281	-0.88109	-4.15857
H	-11.72592	-0.18047	-2.64069
C	-9.45569	-4.47783	-1.20251
H	-9.48868	-3.64967	-1.91783
H	-10.34265	-5.10307	-1.36234
H	-8.57781	-5.08683	-1.43864
C	-9.44864	-3.98045	0.24246
H	-9.30681	-4.84844	0.89803
C	-10.49632	1.77036	-4.27456
H	-11.33854	2.25424	-3.76793
H	-10.88607	1.3228	-5.19726
H	-9.78381	2.54657	-4.56486

C	-6.53033	-3.75395	0.04381
H	-6.77551	-3.91439	-1.01333
C	-6.70507	1.67694	-3.30713
H	-6.44415	0.65097	-3.03665
H	-5.78071	2.26007	-3.39823
H	-7.17602	1.65616	-4.29725
C	-8.05024	-2.7199	2.49472
H	-8.81027	-1.95481	2.69512
C	-6.32877	-5.11563	0.70901
H	-6.00001	-5.01345	1.74837
H	-5.54399	-5.66364	0.17304
H	-7.23004	-5.73703	0.69484
C	-10.78904	-3.3295	0.5907
H	-10.82385	-2.943	1.61473
H	-11.59556	-4.0648	0.48434
H	-11.01069	-2.49588	-0.08696
C	-6.726	-2.19526	3.0547
H	-5.94101	-2.95701	3.0031
H	-6.85981	-1.93816	4.11253
H	-6.38042	-1.29991	2.53021
C	-5.24074	-2.9334	0.06275
H	-5.36298	-1.99257	-0.47806
H	-4.4486	-3.50646	-0.43498
H	-4.89911	-2.71142	1.0773
C	-8.47805	-3.98862	3.23638
H	-9.4951	-4.30435	2.98693
H	-8.45848	-3.78556	4.31434
H	-7.80346	-4.82931	3.05043
Ru	8.46078	-0.77548	-0.46608
Cl	7.48494	-1.56967	-2.57781
P	7.58437	-2.75124	0.63084
S	1.64653	3.93637	2.84425
S	1.65017	2.26914	0.39325
P	9.50089	1.0109	-1.73614
O	10.1689	0.03618	1.82716
C	6.94275	0.28212	0.21801
H	6.12463	0.16245	-0.50378
C	3.14723	3.08807	2.47639
C	3.1537	2.2997	1.31836
C	4.29562	1.61744	0.92383
H	4.26481	1.00145	0.02991
C	4.30622	3.18791	3.24036
H	4.31765	3.78845	4.14547
C	5.45085	2.50978	2.83828
H	6.35155	2.59352	3.44135
C	8.19615	-3.14208	2.35247
H	7.66346	-4.05905	2.63629

C	9.4754	-0.29987	0.94797
C	5.47893	1.71422	1.6786
C	9.69448	-3.43659	2.42953
H	9.96409	-4.35874	1.90819
H	9.98231	-3.56038	3.48085
H	10.29823	-2.62153	2.01866
C	0.68968	3.07851	1.63408
C	5.12877	-3.68503	1.90218
H	5.23404	-4.73126	1.60003
H	4.05429	-3.48222	1.99048
H	5.56006	-3.57395	2.90017
C	7.81274	-2.04133	3.34558
H	8.39222	-1.13121	3.17412
H	8.029	-2.38452	4.36461
H	6.75324	-1.77279	3.3021
C	7.97898	-4.24982	-0.41681
H	7.30406	-4.11779	-1.272
C	6.72275	1.02282	1.32361
H	7.52695	1.15247	2.04836
C	5.71762	-2.72011	0.86978
H	5.55767	-1.69695	1.23634
C	9.40491	-4.18451	-0.97265
H	9.58195	-3.24934	-1.51463
H	9.55444	-5.00529	-1.68508
H	10.16611	-4.28166	-0.19327
C	10.89073	-0.63363	-3.60451
H	10.28305	-1.48852	-3.29106
H	11.86634	-1.01096	-3.93469
H	10.40385	-0.17932	-4.47274
C	11.1006	0.36339	-2.46562
H	11.62452	1.23635	-2.87393
C	7.69989	-5.60851	0.22399
H	8.31706	-5.78202	1.11231
H	7.93652	-6.40201	-0.49568
H	6.65219	-5.73008	0.51001
C	8.56125	1.61938	-3.23244
H	8.50049	0.7004	-3.8284
C	4.96735	-2.85698	-0.45525
H	5.3453	-2.17441	-1.22014
H	3.90442	-2.6431	-0.29022
H	5.03687	-3.87784	-0.84956
C	10.07332	2.47742	-0.70981
H	10.3847	2.00638	0.23046
C	9.27892	2.68819	-4.05715
H	9.29159	3.65477	-3.543
H	8.7421	2.83115	-5.00311
H	10.3109	2.41843	-4.30389

C	11.97454	-0.25121	-1.36997
H	12.1429	0.42673	-0.52656
H	12.95439	-0.51683	-1.78443
H	11.52372	-1.1695	-0.97376
C	8.92033	3.42681	-0.37561
H	8.63573	4.03063	-1.24368
H	9.24155	4.12038	0.41101
H	8.0363	2.8926	-0.0159
C	7.11945	2.02913	-2.93249
H	6.58159	1.23137	-2.41601
H	6.60213	2.2243	-3.88008
H	7.05802	2.93932	-2.32979
C	11.27494	3.2614	-1.24253
H	12.17581	2.6478	-1.33372
H	11.50499	4.06901	-0.5365
H	11.07583	3.72363	-2.21359

Table S30. Atomic coordinates of **DBTTF-(ViRu)$_{2E}^{2+}$ (S)** from its DFT pbe1pbe calculated structure.

Ru	8.52837	-5.54067	1.77496
Cl	7.47497	-7.72728	1.42816
P	7.92362	-5.66949	4.12158
S	1.60163	-0.19778	0.27495
S	1.58099	-3.1343	0.32672
P	9.3438	-5.69378	-0.51037
O	10.29423	-3.17026	2.11048
C	6.97681	-4.4071	1.4121
H	6.12529	-5.07348	1.22434
C	3.12497	-0.96634	0.60751
C	3.12644	-2.37214	0.6323
C	4.28744	-3.08741	0.88503
H	4.25434	-4.17197	0.89845
C	4.31315	-0.27124	0.84606
H	4.32967	0.81398	0.83425
C	5.46703	-0.98906	1.09694
H	6.39131	-0.44821	1.28064
C	8.67922	-4.42126	5.28736
H	8.25525	-4.66813	6.26933
C	9.58366	-4.09063	2.0079
C	5.49348	-2.40504	1.12176
C	10.2007	-4.51441	5.40605
H	10.52425	-5.44265	5.88389
H	10.56228	-3.68545	6.02668
H	10.69913	-4.43697	4.43486
C	0.68619	-1.65932	0.1117
C	5.67174	-5.08498	5.8764
H	5.90124	-5.8752	6.5972

H	4.58498	-4.93716	5.89551
H	6.12913	-4.15805	6.23094
C	8.25377	-2.99422	4.92933
H	8.74794	-2.64869	4.01834
H	8.54711	-2.31732	5.74072
H	7.1748	-2.88984	4.78199
C	8.41082	-7.35792	4.75688
H	7.68111	-8.01008	4.26044
C	6.76141	-3.06866	1.38944
H	7.59318	-2.39021	1.57543
C	6.08124	-5.46247	4.4502
H	5.82577	-4.61974	3.79326
C	9.79109	-7.7708	4.23641
H	9.84618	-7.70535	3.14378
H	9.98235	-8.81663	4.50609
H	10.59944	-7.16736	4.65862
C	10.71876	-8.17545	-0.23408
H	10.20343	-8.35308	0.71532
H	11.69514	-8.67299	-0.1901
H	10.13846	-8.66093	-1.02449
C	10.93153	-6.68534	-0.49791
H	11.36128	-6.57837	-1.50144
C	8.30421	-7.56094	6.26738
H	8.9734	-6.89576	6.82377
H	8.59139	-8.59067	6.51317
H	7.28757	-7.40919	6.63905
C	8.25608	-6.62128	-1.71253
H	8.21978	-7.61086	-1.24086
C	5.27581	-6.67514	3.98268
H	5.52726	-6.97431	2.96238
H	4.20691	-6.43327	4.02324
H	5.43895	-7.53875	4.63821
C	9.87375	-4.05581	-1.26169
H	10.29248	-3.51933	-0.40174
C	8.83316	-6.77368	-3.12031
H	8.81034	-5.82822	-3.67199
H	8.22171	-7.49129	-3.68093
H	9.86203	-7.1476	-3.12386
C	11.91822	-6.09587	0.51265
H	12.09265	-5.02472	0.36576
H	12.88495	-6.60547	0.42449
H	11.5641	-6.23854	1.54134
C	8.67726	-3.24186	-1.75965
H	8.28308	-3.64491	-2.69833
H	8.99983	-2.21266	-1.95794
H	7.86589	-3.20663	-1.02667
C	6.8141	-6.11642	-1.75856

H	6.37501	-6.08166	-0.75923
H	6.21603	-6.80881	-2.3637
H	6.73086	-5.12529	-2.21262
C	10.96647	-4.11458	-2.33155
H	11.90301	-4.53834	-1.95797
H	11.18496	-3.09176	-2.66233
H	10.66011	-4.68453	-3.21312
Ru	-8.53242	-5.52943	-1.76416
Cl	-7.48154	-7.71804	-1.42265
P	-7.94749	-5.66277	-4.11566
S	-1.56961	-0.19627	-0.40116
S	-1.58024	-3.13219	-0.32044
P	-9.3275	-5.67597	0.52856
O	-10.29713	-3.15669	-2.08914
C	-6.97536	-4.39823	-1.41764
H	-6.1247	-5.06591	-1.23076
C	-3.1005	-0.96286	-0.70228
C	-3.11659	-2.36832	-0.66513
C	-4.28411	-3.08223	-0.89015
H	-4.26218	-4.16661	-0.85471
C	-4.28169	-0.26678	-0.97086
H	-4.28784	0.81808	-1.00454
C	-5.44234	-0.98315	-1.19292
H	-6.36098	-0.44126	-1.40034
C	-8.71009	-4.41487	-5.2773
H	-8.29383	-4.66375	-6.26205
C	-9.58711	-4.07796	-1.99083
C	-5.48234	-2.39867	-1.16076
C	-10.23256	-4.50557	-5.38452
H	-10.56137	-5.4339	-5.85858
H	-10.59729	-3.67685	-6.00364
H	-10.72354	-4.42579	-4.40973
C	-0.66987	-1.65855	-0.17138
C	-5.70839	-5.08324	-5.88851
H	-5.94533	-5.87304	-6.60736
H	-4.62148	-4.93785	-5.91622
H	-6.16643	-4.15531	-6.23963
C	-8.27999	-2.98788	-4.92454
H	-8.76966	-2.63932	-4.01227
H	-8.5756	-2.31225	-5.73616
H	-7.20019	-2.88531	-4.78206
C	-8.44253	-7.35113	-4.74494
H	-7.71028	-8.00397	-4.25316
C	-6.75507	-3.06043	-1.40948
H	-7.58479	-2.38116	-1.60141
C	-6.10741	-5.45928	-4.45897
H	-5.84518	-4.61669	-3.80455

C	-9.81957	-7.76067	-4.21342
H	-9.86636	-7.69295	-3.12053
H	-10.01468	-8.80666	-4.47967
H	-10.62998	-7.15659	-4.63073
C	-10.71203	-8.15417	0.26978
H	-10.20809	-8.33475	-0.68518
H	-11.69013	-8.64924	0.23772
H	-10.12401	-8.63985	1.05434
C	-10.91795	-6.66307	0.53338
H	-11.33743	-6.55331	1.54096
C	-8.34753	-7.5563	-6.25591
H	-9.01971	-6.89073	-6.80823
H	-8.63817	-8.58587	-6.49825
H	-7.33338	-7.40663	-6.63518
C	-8.23096	-6.60407	1.72229
H	-8.20049	-7.59413	1.25123
C	-5.30059	-6.6731	-3.99683
H	-5.54447	-6.97076	-2.97425
H	-4.23162	-6.43322	-4.04605
H	-5.47052	-7.53702	-4.65021
C	-9.84607	-4.03497	1.28091
H	-10.27316	-3.50021	0.42398
C	-8.79592	-6.75409	3.13522
H	-8.76607	-5.80822	3.68584
H	-8.1811	-7.47259	3.691
H	-9.82554	-7.12574	3.14822
C	-11.91321	-6.07257	-0.46816
H	-12.08287	-5.00063	-0.32155
H	-12.8805	-6.57907	-0.36896
H	-11.57028	-6.21831	-1.50022
C	-8.64244	-3.2221	1.76307
H	-8.23906	-3.62286	2.69884
H	-8.96039	-2.19145	1.96125
H	-7.83908	-3.1914	1.02106
C	-6.78772	-6.10183	1.75553
H	-6.35726	-6.06778	0.75243
H	-6.18559	-6.79532	2.35536
H	-6.6987	-5.11089	2.2089
C	-10.92713	-4.08842	2.3628
H	-11.86859	-4.51116	2.0006
H	-11.13982	-3.06422	2.6931
H	-10.61244	-4.65662	3.24255

Table S31. Atomic coordinates of **DBTTF-(ViRu)$_{2E}^{2+}$ (S)** from its pbe1pbe calculated DFT structure.

Ru	8.49942	-5.50481	1.76237
Cl	7.39698	-7.66129	1.47808

P	7.9585	-5.59579	4.13688
S	1.59523	-0.2152	0.39109
S	1.58737	-3.1247	0.26613
P	9.33201	-5.76824	-0.51687
O	10.29096	-3.13626	2.01403
C	6.96815	-4.40359	1.39246
H	6.10882	-5.06439	1.22763
C	3.10992	-0.9692	0.66934
C	3.12736	-2.38463	0.61625
C	4.29071	-3.09714	0.82957
H	4.27174	-4.18044	0.78199
C	4.29364	-0.2656	0.93732
H	4.29488	0.81837	0.97921
C	5.44843	-0.98212	1.14495
H	6.36927	-0.44466	1.35184
C	8.75502	-4.33028	5.25328
H	8.35518	-4.56014	6.24942
C	9.57365	-4.04977	1.94442
C	5.48992	-2.4074	1.10433
C	10.27837	-4.42869	5.33555
H	10.61055	-5.35183	5.81691
H	10.65595	-3.5937	5.93781
H	10.75546	-4.36321	4.35298
C	0.69078	-1.65059	0.14443
C	5.76623	-4.96788	5.93991
H	6.02471	-5.74068	6.66952
H	4.68017	-4.82492	5.99327
H	6.23049	-4.03097	6.2567
C	8.32474	-2.90761	4.88341
H	8.80845	-2.57175	3.96317
H	8.63007	-2.22189	5.68241
H	7.24378	-2.80284	4.75099
C	8.463	-7.27878	4.76583
H	7.71703	-7.93449	4.29929
C	6.75216	-3.04902	1.35125
H	7.58612	-2.37208	1.52641
C	6.12502	-5.3765	4.50832
H	5.84935	-4.54685	3.84284
C	9.82616	-7.69432	4.20467
H	9.84167	-7.65279	3.10932
H	10.03163	-8.73387	4.48657
H	10.64656	-7.07965	4.58465
C	10.62187	-8.2826	-0.14093
H	10.09407	-8.41876	0.80805
H	11.58257	-8.80675	-0.07439
H	10.03439	-8.77251	-0.92308
C	10.88415	-6.80844	-0.44561

H	11.32618	-6.74497	-1.44745
C	8.40298	-7.46378	6.28131
H	9.09166	-6.79575	6.8093
H	8.69412	-8.49202	6.52737
H	7.39876	-7.30505	6.6825
C	8.21255	-6.69796	-1.68521
H	8.13809	-7.66769	-1.17843
C	5.30247	-6.59775	4.09621
H	5.51175	-6.91786	3.07305
H	4.2368	-6.35211	4.17467
H	5.48991	-7.44774	4.7624
C	9.91536	-4.16893	-1.30653
H	10.3624	-3.62896	-0.46355
C	8.79502	-6.91873	-3.08207
H	8.80244	-5.99394	-3.66778
H	8.1655	-7.63845	-3.61891
H	9.81205	-7.32287	-3.0648
C	11.87673	-6.21334	0.55585
H	12.09159	-5.15589	0.36873
H	12.82559	-6.75918	0.5005
H	11.50749	-6.30433	1.58512
C	8.7462	-3.32321	-1.81532
H	8.33216	-3.73112	-2.74303
H	9.10463	-2.31097	-2.03585
H	7.93875	-3.24146	-1.08109
C	6.79172	-6.14053	-1.76284
H	6.34591	-6.04429	-0.77043
H	6.17179	-6.83674	-2.34045
H	6.74979	-5.16931	-2.26301
C	10.99745	-4.29798	-2.38144
H	11.91795	-4.7512	-2.00287
H	11.25539	-3.29237	-2.73482
H	10.66206	-4.87328	-3.24851
Ru	-8.5003	-5.48393	-1.76981
Cl	-7.41906	-7.64719	-1.45739
P	-7.95246	-5.60737	-4.14098
S	-1.60341	-0.21059	-0.29063
S	-1.5655	-3.12197	-0.34323
P	-9.34073	-5.71134	0.5106
O	-10.27463	-3.10638	-2.05552
C	-6.96277	-4.38851	-1.40755
H	-6.10597	-5.05266	-1.2419
C	-3.11191	-0.96198	-0.60654
C	-3.11547	-2.37814	-0.63651
C	-4.27438	-3.0882	-0.87987
H	-4.24559	-4.1721	-0.89533
C	-4.30254	-0.25527	-0.8321

H	-4.31268	0.82938	-0.81501
C	-5.45208	-0.96964	-1.07375
H	-6.37853	-0.43006	-1.24748
C	-8.73482	-4.3482	-5.27432
H	-8.33314	-4.59226	-6.26632
C	-9.56361	-4.02373	-1.97261
C	-5.48223	-2.39548	-1.10418
C	-10.25862	-4.43587	-5.36124
H	-10.59579	-5.36135	-5.83461
H	-10.62763	-3.60422	-5.97333
H	-10.73897	-4.35708	-4.38122
C	-0.68362	-1.64896	-0.13387
C	-5.74793	-5.01758	-5.94237
H	-6.00915	-5.79717	-6.66368
H	-4.66059	-4.8835	-5.9927
H	-6.2037	-4.08111	-6.27251
C	-8.29428	-2.92503	-4.91875
H	-8.77539	-2.57586	-4.00212
H	-8.5941	-2.2454	-5.72502
H	-7.2126	-2.8271	-4.78707
C	-8.46704	-7.29361	-4.75324
H	-7.72797	-7.94962	-4.27624
C	-6.74368	-3.03458	-1.36159
H	-7.5782	-2.35554	-1.52561
C	-6.11589	-5.40599	-4.5075
H	-5.83702	-4.57006	-3.8512
C	-9.83559	-7.69275	-4.19332
H	-9.85626	-7.63618	-3.09875
H	-10.04645	-8.73465	-4.46226
H	-10.64999	-7.07786	-4.58571
C	-10.65816	-8.2149	0.16111
H	-10.12948	-8.36796	-0.78486
H	-11.62452	-8.72895	0.09788
H	-10.07838	-8.70243	0.95046
C	-10.9045	-6.73446	0.44833
H	-11.34751	-6.6545	1.44858
C	-8.40216	-7.49616	-6.26625
H	-9.08281	-6.82829	-6.80475
H	-8.70115	-8.52456	-6.50195
H	-7.39486	-7.35073	-6.6647
C	-8.23429	-6.63957	1.6925
H	-8.17223	-7.61736	1.19969
C	-5.30415	-6.62807	-4.07688
H	-5.5201	-6.93411	-3.0508
H	-4.23634	-6.39143	-4.15394
H	-5.49524	-7.48479	-4.73335
C	-9.90714	-4.09635	1.28096

H	-10.34246	-3.55897	0.43024
C	-8.82056	-6.83323	3.09183
H	-8.81778	-5.90028	3.66451
H	-8.20005	-7.55258	3.63954
H	-9.84223	-7.2257	3.07915
C	-11.8886	-6.14001	-0.56187
H	-12.09095	-5.07778	-0.38852
H	-12.84404	-6.67366	-0.50096
H	-11.51918	-6.24833	-1.58938
C	-8.72989	-3.26086	1.78814
H	-8.32688	-3.66442	2.72257
H	-9.07626	-2.24173	1.99593
H	-7.91711	-3.19757	1.05811
C	-6.80616	-6.09992	1.76248
H	-6.35678	-6.03277	0.76926
H	-6.19764	-6.79168	2.35733
H	-6.75172	-5.11835	2.24078
C	-10.99658	-4.20025	2.35114
H	-11.92121	-4.64444	1.97198
H	-11.24254	-3.18783	2.69352
H	-10.67372	-4.77176	3.22546

Table S32. Atomic coordinates of **DBTTF-(ViRu)$_{2E}$$^{2+}$ (T)** from its pbe1pbe calculated DFT structure.

Ru	8.50749	-5.4461	1.76553
Cl	7.44683	-7.60699	1.46823
P	7.98375	-5.58133	4.15436
S	1.59608	-0.21073	0.27215
S	1.58401	-3.17154	0.31181
P	9.41289	-5.72734	-0.49535
O	10.27234	-3.05348	2.04643
C	6.97505	-4.3903	1.3812
H	6.1215	-5.05694	1.21684
C	3.1185	-0.97849	0.57746
C	3.12633	-2.39048	0.60008
C	4.29324	-3.08711	0.83822
H	4.27485	-4.17156	0.85237
C	4.30329	-0.26614	0.79725
H	4.30233	0.81883	0.78151
C	5.46886	-0.96447	1.03384
H	6.39117	-0.41692	1.20406
C	8.76419	-4.31105	5.27425
H	8.36939	-4.56054	6.26778
C	9.56484	-3.96927	1.96415
C	5.49957	-2.38348	1.06191
C	10.28902	-4.38474	5.35436
H	10.63716	-5.30772	5.82435

H	10.65054	-3.55107	5.96774
H	10.7667	-4.29748	4.3738
C	0.66704	-1.68876	0.11379
C	5.78707	-5.01195	5.95967
H	6.06151	-5.78921	6.67835
H	4.699	-4.88943	6.01706
H	6.23472	-4.07043	6.28604
C	8.3056	-2.89248	4.92315
H	8.781	-2.53268	4.00759
H	8.60007	-2.21274	5.73091
H	7.22247	-2.80554	4.79624
C	8.5224	-7.26417	4.75
H	7.78653	-7.92625	4.27669
C	6.75596	-3.02482	1.32066
H	7.59461	-2.35038	1.48189
C	6.14536	-5.3962	4.52093
H	5.85535	-4.56193	3.86788
C	9.89162	-7.64409	4.17913
H	9.90357	-7.5866	3.08453
H	10.11655	-8.68387	4.44385
H	10.7014	-7.02025	4.56641
C	10.71218	-8.22925	-0.07183
H	10.16105	-8.37257	0.86245
H	11.67639	-8.74247	0.02038
H	10.15208	-8.72476	-0.8702
C	10.96917	-6.75293	-0.37017
H	11.43468	-6.68643	-1.36122
C	8.47013	-7.46923	6.26336
H	9.14736	-6.79559	6.79862
H	8.78237	-8.49488	6.49283
H	7.464	-7.33716	6.66903
C	8.3195	-6.67085	-1.67508
H	8.24428	-7.6397	-1.16708
C	5.3414	-6.62536	4.09638
H	5.5446	-6.92608	3.06645
H	4.27323	-6.39717	4.18835
H	5.54836	-7.48193	4.74787
C	9.99568	-4.12198	-1.2709
H	10.42177	-3.57648	-0.42096
C	8.9364	-6.88837	-3.05814
H	8.94467	-5.96592	-3.64719
H	8.32644	-7.61748	-3.6043
H	9.95719	-7.281	-3.0176
C	11.92981	-6.14269	0.65304
H	12.13777	-5.08374	0.46698
H	12.88553	-6.67784	0.62077
H	11.54003	-6.23613	1.67426

C	8.83131	-3.28848	-1.80934
H	8.44553	-3.70012	-2.74722
H	9.18538	-2.27292	-2.01953
H	8.00233	-3.21325	-1.09882
C	6.8967	-6.1244	-1.78505
H	6.42306	-6.03967	-0.80461
H	6.29925	-6.82343	-2.38206
H	6.85805	-5.15113	-2.28111
C	11.10196	-4.24864	-2.32175
H	12.01745	-4.69249	-1.92093
H	11.35901	-3.24227	-2.67283
H	10.78942	-4.8295	-3.19339
Ru	-8.50688	-5.47034	-1.75907
Cl	-7.44469	-7.62795	-1.44469
P	-7.98387	-5.6247	-4.14692
S	-1.59485	-0.21498	-0.34286
S	-1.59069	-3.17577	-0.30208
P	-9.41198	-5.73387	0.50403
O	-10.27428	-3.08209	-2.05984
C	-6.97591	-4.40948	-1.38276
H	-6.12248	-5.07373	-1.20842
C	-3.11842	-0.98671	-0.63203
C	-3.12973	-2.39881	-0.61765
C	-4.29721	-3.09869	-0.84311
H	-4.28114	-4.18319	-0.83004
C	-4.30121	-0.27754	-0.87197
H	-4.29811	0.80749	-0.88308
C	-5.46777	-0.97905	-1.09374
H	-6.3885	-0.43374	-1.27897
C	-8.76537	-4.36391	-5.27682
H	-8.36981	-4.62049	-6.26823
C	-9.56573	-3.99636	-1.96996
C	-5.50117	-2.39827	-1.08868
C	-10.29013	-4.43994	-5.35712
H	-10.63681	-5.3661	-5.82196
H	-10.65241	-3.61031	-5.97551
H	-10.76851	-4.3477	-4.37733
C	-0.6698	-1.69055	-0.14417
C	-5.78795	-5.06674	-5.95699
H	-6.06331	-5.84793	-6.67107
H	-4.69982	-4.94525	-6.01566
H	-6.23516	-4.12682	-6.28851
C	-8.30852	-2.94214	-4.93648
H	-8.78638	-2.57514	-4.02509
H	-8.6017	-2.26935	-5.7505
H	-7.22573	-2.85328	-4.8079
C	-8.5219	-7.31256	-4.72872

H	-7.78592	-7.97047	-4.24976
C	-6.75723	-3.04325	-1.33983
H	-7.59537	-2.37137	-1.51388
C	-6.14561	-5.44222	-4.51576
H	-5.85474	-4.60415	-3.86801
C	-9.8911	-7.68831	-4.15514
H	-9.90328	-7.6219	-3.06105
H	-10.11563	-8.73029	-4.41145
H	-10.70101	-7.06786	-4.54757
C	-10.71314	-8.23734	0.09747
H	-10.16224	-8.38718	-0.83593
H	-11.67764	-8.75061	0.00879
H	-10.15316	-8.72791	0.89897
C	-10.96915	-6.75891	0.38616
H	-11.4344	-6.68553	1.37686
C	-8.46889	-7.52993	-6.2403
H	-9.14665	-6.86144	-6.78133
H	-8.77987	-8.55777	-6.46155
H	-7.46273	-7.39999	-6.64658
C	-8.31941	-6.66962	1.69073
H	-8.24477	-7.64218	1.18974
C	-5.34198	-6.66912	-4.08405
H	-5.54487	-6.96318	-3.05212
H	-4.27376	-6.44197	-4.1779
H	-5.54956	-7.52963	-4.73013
C	-9.99309	-4.12217	1.26738
H	-10.4188	-3.58279	0.41334
C	-8.93642	-6.87684	3.07534
H	-8.94463	-5.95011	3.65765
H	-8.32648	-7.60196	3.62682
H	-9.9572	-7.26977	3.0377
C	-11.92952	-6.15474	-0.64092
H	-12.13728	-5.09461	-0.46143
H	-12.88537	-6.68947	-0.60551
H	-11.53955	-6.25445	-1.66148
C	-8.82771	-3.28576	1.79902
H	-8.44206	-3.69056	2.73993
H	-9.18065	-2.26823	2.00145
H	-7.99897	-3.21694	1.08754
C	-6.89619	-6.12342	1.79689
H	-6.42222	-6.04638	0.81597
H	-6.29943	-6.81846	2.39922
H	-6.85684	-5.14653	2.28571
C	-11.09921	-4.2398	2.31941
H	-12.0151	-4.68614	1.92227
H	-11.35543	-3.23056	2.66276
H	-10.78687	-4.81416	3.19541

Table S33. Atomic coordinates of **DBTTF-(ViRu)$_{2E}$$^{2+}$ (OSS)** from its pbe1pbe calculated DFT structure.

Ru	-8.69073	0.18022	-0.72272
Cl	-7.60907	0.18496	-2.89564
P	-8.51146	2.62373	-0.69584
S	-1.62883	-0.74552	4.44115
S	-1.61474	-0.29001	1.52464
P	-9.25126	-2.16433	-1.15134
O	-10.46162	0.04193	1.67673
C	-7.11539	-0.0529	0.31846
H	-6.24771	-0.02944	-0.35009
C	-3.17682	-0.59629	3.68937
C	-3.18725	-0.38043	2.29198
C	-4.37579	-0.24925	1.60516
H	-4.35859	-0.08022	0.53416
C	-4.38325	-0.67532	4.39656
H	-4.38179	-0.84261	5.46852
C	-5.56881	-0.53896	3.70751
H	-6.50758	-0.5989	4.25037
C	-9.45952	3.53123	0.62897
H	-9.23357	4.58899	0.44046
C	-9.75675	0.12516	0.75842
C	-5.60366	-0.32739	2.30243
C	-10.97652	3.36249	0.53895
H	-11.39221	3.82073	-0.36171
H	-11.44022	3.85817	1.39986
H	-11.28479	2.31288	0.56219
C	-0.68494	-0.55365	2.98549
C	-6.59037	4.69346	-0.02426
H	-6.87625	5.38355	-0.82295
H	-5.53049	4.87379	0.19126
H	-7.15185	4.9572	0.87503
C	-8.95089	3.18016	2.02988
H	-9.22872	2.16303	2.31677
H	-9.41045	3.86013	2.75636
H	-7.8656	3.2739	2.13099
C	-9.11442	3.2628	-2.34206
H	-8.30272	2.97068	-3.02027
C	-6.88607	-0.20433	1.67411
H	-7.73747	-0.24726	2.35045
C	-6.74706	3.22853	-0.44223
H	-6.40611	2.60289	0.39396
C	-10.37096	2.52017	-2.80545
H	-10.21528	1.43604	-2.83229
H	-10.61629	2.83117	-3.82772
H	-11.24133	2.72994	-2.17795
C	-10.61809	-1.78334	-3.62398

H	-10.20376	-0.7743	-3.71122
H	-11.59005	-1.79622	-4.13075
H	-9.95644	-2.46483	-4.16663
C	-10.818	-2.2027	-2.16821
H	-11.13745	-3.25207	-2.16191
C	-9.30901	4.77646	-2.42317
H	-10.09156	5.12973	-1.74355
H	-9.61956	5.03927	-3.4413
H	-8.39357	5.33203	-2.20785
C	-8.00758	-3.10781	-2.17178
H	-8.01526	-2.52984	-3.10367
C	-5.84841	2.93484	-1.64326
H	-5.90238	1.89313	-1.96599
H	-4.81091	3.16331	-1.37266
H	-6.10871	3.56689	-2.49985
C	-9.70025	-3.12443	0.39661
H	-10.23343	-2.38282	1.00274
C	-8.41933	-4.54764	-2.48518
H	-8.34062	-5.19301	-1.60462
H	-7.7391	-4.95017	-3.24501
H	-9.43637	-4.62827	-2.88145
C	-11.91113	-1.36621	-1.49885
H	-12.07983	-1.6414	-0.45243
H	-12.85699	-1.50639	-2.03422
H	-11.67513	-0.29519	-1.53235
C	-8.46175	-3.55169	1.18675
H	-7.95282	-4.39406	0.70796
H	-8.77074	-3.88174	2.1851
H	-7.74042	-2.73825	1.31152
C	-6.57857	-3.04716	-1.63351
H	-6.25127	-2.01652	-1.48037
H	-5.90769	-3.50163	-2.37209
H	-6.45884	-3.59812	-0.69701
C	-10.65021	-4.3086	0.19876
H	-11.62071	-4.00887	-0.20613
H	-10.83748	-4.76666	1.17721
H	-10.22943	-5.08148	-0.44983
Ru	8.69359	0.09395	-0.73479
Cl	7.59671	0.58549	-2.84441
P	8.74083	-2.26756	-1.37243
S	1.62738	-0.87759	4.41719
S	1.6104	-0.38487	1.50693
P	9.02178	2.50921	-0.49396
O	10.47403	-0.26427	1.6343
C	7.11257	-0.1102	0.3045
H	6.24497	-0.01191	-0.35731
C	3.1747	-0.75353	3.66006

C	3.1841	-0.51072	2.26685
C	4.37228	-0.38557	1.57817
H	4.35449	-0.19271	0.51113
C	4.38211	-0.87998	4.35863
H	4.38217	-1.06973	5.42675
C	5.56715	-0.75542	3.66688
H	6.50668	-0.848	4.20378
C	9.75948	-3.41712	-0.31528
H	9.6124	-4.40162	-0.77824
C	9.76693	-0.1587	0.71972
C	5.60089	-0.50256	2.26855
C	11.2596	-3.12293	-0.32608
H	11.71048	-3.29889	-1.30568
H	11.75646	-3.79237	0.38593
H	11.48869	-2.09697	-0.02214
C	0.682	-0.60033	2.97678
C	7.02751	-4.60723	-1.29147
H	7.46024	-5.06625	-2.1848
H	5.98312	-4.93615	-1.23371
H	7.54144	-5.01131	-0.41623
C	9.2275	-3.48163	1.1194
H	9.46293	-2.57103	1.67562
H	9.71025	-4.31567	1.64155
H	8.14611	-3.63859	1.17393
C	9.40699	-2.36623	-3.11189
H	8.58125	-1.94746	-3.70091
C	6.88352	-0.37135	1.64281
H	7.73586	-0.48344	2.30997
C	7.04055	-3.07714	-1.35919
H	6.60397	-2.69586	-0.42601
C	10.6057	-1.43319	-3.30591
H	10.366	-0.40138	-3.02386
H	10.88064	-1.41561	-4.36704
H	11.48609	-1.74999	-2.74058
C	10.38865	2.95471	-2.95729
H	10.07585	1.97225	-3.32364
H	11.34818	3.20245	-3.42603
H	9.65584	3.68964	-3.30297
C	10.56405	2.97648	-1.43958
H	10.78206	4.00942	-1.14159
C	9.7094	-3.77623	-3.6173
H	10.49238	-4.26958	-3.03184
H	10.06709	-3.71344	-4.65184
H	8.82587	-4.41933	-3.61729
C	7.67962	3.57519	-1.22931
H	7.73316	3.28653	-2.28582
C	6.16305	-2.60144	-2.51731

H	6.12608	-1.51291	-2.59765
H	5.14363	-2.97466	-2.36529
H	6.51653	-3.001	-3.47475
C	9.39827	3.04508	1.26454
H	10.00451	2.21794	1.65161
C	7.94475	5.07795	-1.11778
H	7.80612	5.43884	-0.09372
H	7.22469	5.61039	-1.75033
H	8.94724	5.36403	-1.45116
C	11.73825	2.09405	-1.00962
H	11.8924	2.08394	0.07451
H	12.66115	2.46222	-1.47171
H	11.6008	1.05688	-1.34029
C	8.13515	3.12403	2.12434
H	7.54419	4.01382	1.88576
H	8.42449	3.19756	3.17884
H	7.49423	2.24389	2.0141
C	6.26967	3.22671	-0.7539
H	6.04384	2.16975	-0.91102
H	5.54954	3.80994	-1.33992
H	6.10959	3.4688	0.29994
C	10.23289	4.32004	1.41248
H	11.22113	4.23453	0.95215
H	10.39076	4.50582	2.48152
H	9.73388	5.19966	0.99751

Table S34. Atomic coordinates of **DBTTF-(ViRu)$_{2E}^{2+}$ (S)** from its M062X calculated DFT structure.

Ru	-1.08547	1.72949	0.01272
Cl	-1.25867	2.00112	-2.48159
P	-1.24062	-0.66465	-0.28015
S	-9.66133	1.97436	1.96358
S	-8.49153	1.76745	-0.67353
P	-0.65354	4.11432	0.13494
O	-0.41172	1.60240	2.89499
C	-2.97482	1.80664	0.40783
H	-3.51258	1.85893	-0.54733
C	-7.94845	1.91959	1.92682
C	-7.36034	1.81649	0.64512
C	-5.98455	1.75650	0.48012
H	-5.56625	1.66027	-0.51551
C	-7.13190	1.96429	3.06998
H	-7.56727	2.04078	4.05990
C	-5.77072	1.91006	2.89565
H	-5.12861	1.94690	3.76982
C	-0.85764	-1.72797	1.19633
H	-0.97139	-2.76218	0.84960
C	-0.68281	1.61689	1.76943
C	-5.15618	1.81169	1.61121
C	0.57311	-1.56357	1.71822
H	1.31053	-1.98809	1.03329
H	0.66966	-2.08994	2.67381
H	0.83358	-0.51445	1.89267
C	-9.90101	1.87082	0.28961
C	-3.19598	-2.78084	-0.58376
H	-2.53731	-3.40002	-1.19831
H	-4.22495	-2.99947	-0.88887
H	-3.08893	-3.09685	0.45604
C	-1.87794	-1.47483	2.31487
H	-1.71449	-0.50201	2.78490
H	-1.76078	-2.23920	3.08995
H	-2.91417	-1.50568	1.96523
C	-0.02863	-1.12704	-1.61492
H	-0.50784	-0.74573	-2.52452
C	-3.70734	1.77229	1.55121
H	-3.20526	1.74051	2.51690
C	-2.93560	-1.28412	-0.79221
H	-3.60762	-0.72751	-0.12443
C	1.27853	-0.34016	-1.43884
H	1.09671	0.74108	-1.41121
H	1.93603	-0.53181	-2.29355
H	1.81904	-0.62354	-0.53208
C	1.60830	4.13274	-1.57215
H	1.31826	3.13611	-1.92059
H	2.69836	4.21530	-1.63718
H	1.17771	4.86646	-2.25932
C	1.17377	4.37314	-0.12446
H	1.38525	5.41789	0.12898
C	0.23681	-2.62500	-1.77520
H	0.65098	-3.06779	-0.86359
H	0.96737	-2.77904	-2.57645
H	-0.66672	-3.17701	-2.04363
C	-1.45540	5.15665	-1.18069
H	-1.00615	4.74066	-2.08955
C	-3.27166	-0.88894	-2.23286
H	-3.11288	0.17529	-2.41942
H	-4.32028	-1.13136	-2.43540
H	-2.66007	-1.45254	-2.94663

C	-0.96118	4.87492	1.81919
H	-0.70221	4.06837	2.51473
C	-1.11393	6.64699	-1.09971
H	-1.61478	7.12817	-0.25401
H	-1.46016	7.14414	-2.01202
H	-0.03820	6.82855	-1.01087
C	1.95713	3.46686	0.83320
H	1.62763	3.56171	1.87373
H	3.02226	3.71570	0.79322
H	1.85383	2.41253	0.54352
C	-2.44446	5.19873	2.03302
H	-2.73914	6.08808	1.46754
H	-2.61558	5.41247	3.09318
H	-3.09638	4.36906	1.74362
C	-2.96529	4.93109	-1.30462
H	-3.19080	3.87329	-1.45596
H	-3.33254	5.48089	-2.17814
H	-3.51513	5.29008	-0.43125
C	-0.08706	6.08232	2.17772
H	0.97497	5.82834	2.22444
H	-0.38048	6.44393	3.16910
H	-0.21204	6.90908	1.47359
C	-11.21726	1.87391	-0.28090
S	-11.45693	1.77445	-1.95506
S	-12.62690	1.97498	0.68244
C	-13.16944	1.82859	-1.91841
C	-13.75795	1.92925	-0.63646
C	-13.98609	1.78538	-3.06168
C	-15.13358	1.98841	-0.47142
H	-13.55077	1.71037	-4.05173
C	-15.34706	1.84132	-2.88734
H	-15.55139	2.07818	0.52464
C	-15.96205	1.93984	-1.60280
H	-15.98893	1.80797	-3.76181
C	-17.41033	1.98873	-1.54541
C	-18.14759	2.02430	-0.40459
H	-17.90974	1.99780	-2.51293
Ru	-20.04213	2.05116	-0.02791
H	-17.61730	1.99188	0.55591
C	-20.43392	2.04002	-1.79102
O	-20.69521	2.09179	-2.91775
H	-17.80424	3.99789	1.50383
C	-17.96967	5.07410	1.41818
C	-19.46376	5.39393	1.30949
H	-17.57327	5.54751	2.32310
H	-17.39843	5.45398	0.56758
P	-20.32609	4.46121	-0.04966
H	-19.93687	4.96425	2.19985
C	-19.71483	6.90434	1.29716
C	-22.13522	4.81662	0.22134
C	-19.97230	5.27397	-1.69932
H	-19.18575	7.39225	0.47269
H	-19.33910	7.33792	2.23001
H	-20.77754	7.15477	1.21970
C	-22.58946	4.53664	1.65571
H	-22.28303	5.88279	0.01666
C	-22.96760	4.00451	-0.77878
H	-20.28292	4.51709	-2.42868
C	-18.47150	5.51165	-1.90223
C	-20.76750	6.54945	-2.00126
H	-22.36480	3.50852	1.95753

H	-23.67228	4.68563	1.72359
H	-22.11535	5.20851	2.37673
H	-22.63185	4.13206	-1.81384
H	-24.01687	4.31135	-0.72488
H	-22.92501	2.93273	-0.54231
H	-18.12234	6.35629	-1.30039
H	-18.28550	5.75788	-2.95273
H	-17.87378	4.63113	-1.64807
H	-21.84359	6.36635	-2.05651
H	-20.45131	6.93568	-2.97623
H	-20.58992	7.33406	-1.26122
H	-22.32140	1.16851	1.28182
C	-22.57630	0.10128	1.27479
C	-21.32943	-0.77683	1.45656
H	-23.26490	-0.06744	2.10956
H	-23.11330	-0.11915	0.34877
P	-20.05265	-0.35587	0.16961
H	-20.85084	-0.45616	2.38962
C	-21.69354	-2.25873	1.56111
C	-20.47187	-1.32767	-1.35864
C	-18.42516	-1.12218	0.70387
H	-22.10643	-2.64698	0.62436
H	-22.45574	-2.39119	2.33632
H	-20.83389	-2.87423	1.83678
H	-20.42333	-2.38126	-1.05827
C	-21.88060	-1.05620	-1.89492
C	-19.41801	-1.08177	-2.44778
C	-18.25237	-2.61297	0.38786
H	-17.68459	-0.56255	0.11611
C	-18.14203	-0.86493	2.18689
H	-22.65369	-1.47525	-1.24692
H	-21.98657	-1.52385	-2.87962
H	-22.07934	0.01385	-2.01533
H	-19.53837	-0.09173	-2.89402
H	-19.54309	-1.82069	-3.24595
H	-18.39129	-1.15566	-2.07660
H	-18.99640	-3.22899	0.89989
H	-17.26620	-2.93044	0.74312
H	-18.30323	-2.83563	-0.67999
H	-18.24999	0.18880	2.45241
H	-17.12063	-1.18534	2.41751
H	-18.81974	-1.44752	2.82128
Cl	-19.87212	2.20888	2.47566

Table S35. Atomic coordinates of **DBTTF-(ViRu)$_{2E}^{2+}$ (T)** from its M062X calculated DFT structure.

Ru	-0.99666	1.77848	-0.01235
Cl	-1.16762	2.10966	-2.50568
P	-1.20983	-0.60281	-0.35419
S	-9.58027	2.13448	2.05343
S	-8.40868	1.56535	-0.57669
P	-0.56152	4.15432	0.17449
O	-0.27615	1.56847	2.85048
C	-2.89362	1.87404	0.43212
H	-3.45004	1.95769	-0.51090
C	-7.84490	2.05412	1.99169
C	-7.27636	1.78608	0.73598
C	-5.89919	1.69243	0.57175
H	-5.48963	1.45678	-0.40502
C	-7.01712	2.23156	3.10594
H	-7.44293	2.43401	4.08266
C	-5.65004	2.14623	2.93433
H	-5.00166	2.28850	3.79339
C	-0.83972	-1.71152	1.09292
H	-0.98721	-2.73478	0.72703
C	-0.56727	1.61587	1.72890
C	-5.05727	1.88726	1.67381
C	0.60132	-1.59790	1.59977
H	1.31756	-2.03331	0.89917
H	0.69437	-2.14108	2.54642
H	0.89382	-0.55928	1.78569
C	-9.83280	1.81241	0.37729
C	-3.22461	-2.66389	-0.67907
H	-2.58685	-3.28994	-1.30858
H	-4.26121	-2.84867	-0.98107
H	-3.12007	-3.00155	0.35425
C	-1.83793	-1.45230	2.22973
H	-1.64313	-0.49187	2.71295
H	-1.73138	-2.23337	2.98977
H	-2.87880	-1.44843	1.89293
C	-0.02739	-1.07570	-1.71297
H	-0.50461	-0.65793	-2.60751
C	-3.59723	1.82837	1.58252
H	-3.07840	1.78564	2.53981
C	-2.92410	-1.17137	-0.86070
H	-3.57449	-0.61079	-0.17536
C	1.30708	-0.33589	-1.53737
H	1.16024	0.74894	-1.47467
H	1.94437	-0.52451	-2.40808
H	1.85243	-0.66260	-0.64825
C	1.71062	4.21229	-1.51537
H	1.41591	3.22497	-1.88636
H	2.80165	4.28925	-1.57308
H	1.28814	4.96301	-2.18917
C	1.26826	4.42317	-0.06531
H	1.47874	5.46227	0.21118
C	0.18807	-2.57692	-1.91237
H	0.58961	-3.05655	-1.01365
H	0.91076	-2.73555	-2.72003
H	-0.73427	-3.09154	-2.19109
C	-1.34568	5.23437	-1.12201
H	-0.89436	4.83292	-2.03639
C	-3.26240	-0.73745	-2.28968
H	-3.07003	0.32468	-2.45668
H	-4.32058	-0.94081	-2.48618
H	-2.67694	-1.30677	-3.02089

C	-0.88280	4.87944	1.87295
H	-0.64982	4.05060	2.55175
C	-0.99242	6.71975	-1.00954
H	-1.48864	7.18678	-0.15321
H	-1.33482	7.24002	-1.91055
H	0.08483	6.89072	-0.91713
C	2.04879	3.49692	0.87521
H	1.70945	3.56250	1.91480
H	3.11298	3.75235	0.85093
H	1.95232	2.45030	0.55729
C	-2.36272	5.22751	2.07452
H	-2.63118	6.13724	1.52852
H	-2.54435	5.41783	3.13764
H	-3.02619	4.41900	1.75476
C	-2.85635	5.02356	-1.26186
H	-3.09030	3.96884	-1.42161
H	-3.21084	5.58292	-2.13474
H	-3.41071	5.38141	-0.39097
C	0.00584	6.06262	2.27401
H	1.06323	5.79136	2.32826
H	-0.29549	6.40574	3.26967
H	-0.09687	6.90784	1.58808
C	-11.10597	1.75672	-0.18103
S	-11.33899	1.44955	-1.87454
S	-12.53765	1.99223	0.76414
C	-13.08249	1.53993	-1.82390
C	-13.65484	1.79238	-0.56995
C	-13.88981	1.38313	-2.95316
C	-15.03315	1.88312	-0.41960
H	-13.44908	1.19283	-3.92541
C	-15.26069	1.48105	-2.80557
H	-15.45506	2.08998	0.55780
C	-15.85312	1.72259	-1.54661
H	-15.89886	1.36501	-3.67541
C	-17.30410	1.80592	-1.47735
C	-18.04942	1.89045	-0.32483
H	-17.80861	1.79350	-2.44170
Ru	-19.92002	2.01996	-0.05305
H	-17.54725	1.85784	0.64681
C	-20.25802	2.01498	-1.85997
O	-20.49554	2.07101	-2.98146
H	-17.74522	3.95222	1.51272
C	-17.91635	5.02700	1.42502
C	-19.40910	5.34954	1.30929
H	-17.53090	5.49971	2.33413
H	-17.33958	5.41174	0.58096
P	-20.27382	4.45176	-0.06748
H	-19.89665	4.92250	2.19252
C	-19.65399	6.86221	1.28617
C	-22.08803	4.76675	0.17901
C	-19.88830	5.23382	-1.72086
H	-19.10353	7.34419	0.47278
H	-19.29158	7.28948	2.22635
H	-20.71300	7.12096	1.19114
C	-22.55511	4.51092	1.61384
H	-22.23237	5.83066	-0.04039
C	-22.89427	3.94675	-0.83609
H	-20.22269	4.48833	-2.44991
C	-18.38130	5.43048	-1.91725
C	-20.65424	6.52909	-2.01936
H	-22.32569	3.49528	1.94880

H	-23.63960	4.65031	1.66076
H	-22.09929	5.20843	2.32174
H	-22.56199	4.10541	-1.86776
H	-23.94973	4.22864	-0.77883
H	-22.83278	2.87182	-0.62133
H	-18.01437	6.27217	-1.32284
H	-18.18576	5.65852	-2.96915
H	-17.80146	4.54044	-1.65146
H	-21.73373	6.37005	-2.07656
H	-20.32727	6.90287	-2.99488
H	-20.45659	7.31033	-1.28196
H	-22.37777	1.41617	1.18540
C	-22.75346	0.38595	1.17111
C	-21.62514	-0.62485	1.41310
H	-23.48810	0.30786	1.97878
H	-23.27543	0.21935	0.22563
P	-20.26219	-0.39430	0.17459
H	-21.14639	-0.35198	2.36067
C	-22.15529	-2.05742	1.50793
C	-20.72268	-1.30540	-1.37444
C	-18.73748	-1.30240	0.78495
H	-22.58277	-2.40130	0.56099
H	-22.94966	-2.09219	2.26019
H	-21.38108	-2.76441	1.81446
H	-20.80688	-2.35206	-1.05762
C	-22.07071	-0.88599	-1.96837
C	-19.60051	-1.20588	-2.41793
C	-18.72496	-2.80606	0.47758
H	-17.91833	-0.83114	0.22694
C	-18.49768	-1.07328	2.28015
H	-22.90969	-1.20099	-1.34453
H	-22.18992	-1.36295	-2.94638
H	-22.14676	0.19563	-2.12096
H	-19.60156	-0.23115	-2.91134
H	-19.76609	-1.95995	-3.19328
H	-18.60347	-1.37083	-1.99859
H	-19.54571	-3.33196	0.97192
H	-17.79013	-3.22175	0.86681
H	-18.76437	-3.03199	-0.58950
H	-18.48406	-0.01491	2.54522
H	-17.53421	-1.51537	2.55165
H	-19.26766	-1.56956	2.88086
Cl	-19.83096	2.17733	2.40485

Table S36. Atomic coordinates of **DBTTF-(ViRu)$_{2E}^{2+}$ (OSS)** from its M062X calculated DFT structure.

Ru	3.05549	23.79785	13.41241
Cl	4.36602	22.82380	15.27999
P	1.02439	23.10972	14.57493
S	2.59902	16.85549	8.07155
S	3.82203	16.92638	10.78831
P	5.13272	24.85269	12.67096
O	1.65460	25.46673	11.38379
C	3.05474	22.27800	12.30669
H	3.53992	21.45243	12.83854
C	2.57116	18.38839	8.89215
C	3.15325	18.43657	10.17660
C	3.16211	19.60963	10.90046
H	3.61692	19.62452	11.88485
C	1.99177	19.53429	8.33572
H	1.54548	19.49790	7.34814
C	1.99885	20.70829	9.06131
H	1.55179	21.60176	8.63708
C	-0.56905	23.77556	13.89563
H	-1.34959	23.34371	14.53338
C	2.16344	24.79679	12.16901
C	2.57867	20.77751	10.35257
C	-0.68737	25.30087	13.97548
H	-0.82625	25.64766	15.00184
H	-1.56054	25.62168	13.39812
H	0.18719	25.81233	13.55953
C	3.52973	16.00835	9.30748
C	-0.70741	20.82446	14.85207
H	-1.06924	21.15666	15.82867
H	-0.74522	19.73039	14.84480
H	-1.40257	21.18119	14.08951
C	-0.78799	23.27753	12.45958
H	-0.15704	23.81947	11.75105
H	-1.82824	23.45798	12.17161
H	-0.58771	22.20862	12.33803
C	1.19309	23.69407	16.32960
H	1.95957	23.02529	16.73820
C	2.53996	22.03475	11.04158
H	2.06273	22.84986	10.50024
C	0.74377	21.25397	14.59894
H	1.02003	20.93816	13.58419
C	1.77088	25.11507	16.37140
H	2.72265	25.17860	15.82943
H	1.98028	25.38980	17.41021
H	1.08687	25.86049	15.95857
C	5.68805	26.26009	15.06692
H	4.96826	25.62337	15.59059
H	5.70385	27.23673	15.56117
H	6.67866	25.81243	15.18370
C	5.31462	26.45600	13.59563
H	6.13182	26.99527	13.10373
C	-0.08089	23.57413	17.16830
H	-0.90593	24.15888	16.74952
H	0.11650	23.95672	18.17490
H	-0.41013	22.53739	17.26953
C	6.67683	23.89165	13.04934
H	6.65460	23.86162	14.14420
C	1.67944	20.55163	15.58760
H	2.72603	20.82243	15.43647
H	1.57532	19.46928	15.46278
H	1.40797	20.79123	16.62163

C	5.10387	25.36401	10.87205
H	4.06410	25.66382	10.70083
C	7.95974	24.59694	12.59749
H	8.06867	24.57061	11.50912
H	8.82006	24.07317	13.02629
H	8.00968	25.63979	12.92560
C	4.02940	27.28074	13.45421
H	3.70867	27.39318	12.41287
H	4.18770	28.28220	13.86536
H	3.20346	26.82447	14.01580
C	5.40092	24.18198	9.94266
H	6.46558	23.93075	9.95327
H	5.13800	24.45927	8.91736
H	4.83169	23.28464	10.20616
C	6.63797	22.43833	12.56778
H	5.75324	21.91918	12.94206
H	7.51853	21.91893	12.95980
H	6.66430	22.35931	11.47869
C	5.98597	26.56851	10.52063
H	5.66770	27.48061	11.03155
H	5.90634	26.75202	9.44425
H	7.04003	26.39351	10.74967
Ru	5.71748	7.50665	4.41170
Cl	4.34241	8.44928	2.57405
P	3.72492	6.36498	5.22777
S	4.91208	13.90369	10.35755
S	3.65262	13.82196	7.65785
P	7.67492	8.30416	3.18092
O	7.60444	6.05945	6.20257
C	5.50280	8.92758	5.62186
H	4.81300	9.66048	5.18950
C	5.26023	12.50566	9.38399
C	4.67639	12.45912	8.10002
C	4.89694	11.38403	7.26604
H	4.44678	11.37397	6.27929
C	6.06975	11.45482	9.83028
H	6.52020	11.49104	10.81595
C	6.28937	10.37777	8.99588
H	6.91860	9.55991	9.33216
C	3.98789	5.09573	6.55663
H	2.98321	4.72163	6.78754
C	6.84692	6.61621	5.53882
C	5.71364	10.31284	7.70247
C	4.84276	3.90200	6.12039
H	4.32224	3.26568	5.40151
H	5.07076	3.28897	6.99843
H	5.79666	4.20750	5.67786
C	3.97310	14.74935	9.12704
C	1.43850	6.84007	6.91754
H	0.82909	6.09856	6.39442
H	0.75985	7.60952	7.29944
H	1.90225	6.35656	7.77958
C	4.56311	5.75232	7.81977
H	5.62467	5.98172	7.69872
H	4.47581	5.05492	8.65860
H	4.04953	6.67733	8.09870
C	2.95806	5.51606	3.76447
H	2.55004	6.35023	3.18184
C	5.99863	9.16210	6.89616
H	6.66774	8.42979	7.34487
C	2.45035	7.51675	5.98362

H	3.05815	8.20735	6.58338
C	4.03915	4.85261	2.90028
H	4.80664	5.57355	2.59221
H	3.58227	4.46624	1.98353
H	4.53081	4.01858	3.40696
C	7.15114	6.95691	0.74269
H	6.12146	6.78877	1.07287
H	7.43923	6.13106	0.08440
H	7.16850	7.87575	0.15026
C	8.12671	7.01357	1.92041
H	9.11197	7.30047	1.53587
C	1.83065	4.54050	4.10821
H	2.17313	3.72915	4.75820
H	1.45696	4.08703	3.18446
H	0.98853	5.03867	4.59381
C	7.40302	9.86514	2.21007
H	6.63767	9.54869	1.49292
C	1.71507	8.33299	4.91644
H	2.39829	8.85103	4.24101
H	1.08360	9.07556	5.41420
H	1.05929	7.69285	4.31595
C	9.20590	8.45283	4.24546
H	9.11527	7.62022	4.95158
C	8.64448	10.32624	1.43960
H	9.40230	10.74015	2.11163
H	8.35368	11.12023	0.74442
H	9.10174	9.52338	0.85307
C	8.24727	5.64724	2.60637
H	8.90453	5.66733	3.48252
H	8.65247	4.91545	1.90112
H	7.26375	5.27998	2.92828
C	9.20417	9.74932	5.06280
H	9.43364	10.61315	4.43217
H	9.97935	9.68714	5.83258
H	8.24674	9.93058	5.56184
C	6.79351	11.00762	3.02813
H	5.85252	10.70419	3.49163
H	6.57643	11.84111	2.35193
H	7.47008	11.37659	3.80241
C	10.53779	8.27533	3.50594
H	10.64619	7.27518	3.07908
H	11.35235	8.41397	4.22420
H	10.67194	9.00873	2.70719

References

(1) J. Nakayama, E. Seki, M. Hoshino. General synthesis of dibenzotetrathiafulvalenes. *J. Chem. Soc., Perkin Trans. 1* **1978** (5), 468–471. DOI: 10.1039/P19780000468.

(2) M. Krejčik, M. Daněk, F. Hartl. Simple construction of an infrared optically transparent thin-layer electrochemical cell: Applications to the redox reactions of ferrocene, $Mn_2(CO)_{10}$ and $Mn(CO)_3$(3,5-di-*t*-butyl-catecholate)$^-$. *J. Electroanal. Chem.* **1991**, *317* (1-2), 179–187. DOI: 10.1016/0022-0728(91)85012-E.

(3) Stoll, S.; Schweiger, A. EasySpin, a comprehensive software package for spectral simulation and analysis in EPR. *J. Magn. Reson.* **2006**, *178* (1), 42–55. DOI: 10.1016/j.jmr.2005.08.013.

(4) M. J. Frisch, G. W. Trucks, H. B. Schlegel, G. E. Scuseria, M. A. Robb, J. R. Cheeseman, G. Scalmani, V. Barone, G. A. Petersson, H. Nakatsuji, X. Li, M. Caricato, A. Marenich, J. Bloino, B. G. Janesko, R. Gomperts, B. Mennucci, H. P. Hratchian, J. V. Ortiz, A. F. Izmaylov, J. L. Sonnenberg, D. Williams-Young, F. Ding, F. Lipparini, F. Egidi, J. Goings, B. Peng, A. Petrone, T. Henderson, D. Ranasinghe, V. G. Zakrzewski, J. Gao, N. Rega, G. Zheng, W. Liang, M. Hada, M. Ehara, K. Toyota, R. Fukuda, J. Hasegawa, M. Ishida, T. Nakajima, Y. Honda, O. Kitao, H. Nakai, T. Vreven, K. Throssell, J. J. A. Montgomery, J. E. Peralta, F. Ogliaro, M. Bearpark, J. J. Heyd, E. Brothers, K. N. Kudin, V. N. Staroverov, T. Keith, R. Kobayashi, J. Normand, K. Raghavachari, A. Rendell, J. C. Burant, S. S. Iyengar, J. Tomasi, M. Cossi, J. M. Millam, M. Klene, C. Adamo, R. Cammi, J. W. Ochterski, R. L. Martin, K. Morokuma, O. Farkas, J. B. Foresman, D. J. Fox. GAUSSIAN09. *Gaussian 09, revision D.01; Gaussian, Inc.: Wallingford, CT, 2009.*

(5) Grimme, S.; Antony, J.; Ehrlich, S.; Krieg, H. A consistent and accurate ab initio parametrization of density functional dispersion correction (DFT-D) for the 94 elements H-Pu. *J. Chem. Phys.* **2010**, *132* (15), 154104. DOI: 10.1063/1.3382344.

(6) M. Cossi, N. Rega, G. Scalmani,V. Barone. Energies, structures, and electronic properties of molecules in solution with the C-PCM solvation model. *J. Comput. Chem.* **2003**, 669–681. DOI: 10.1002/jcc.10189.

(7) P. C. Hariharan and J. A. Pople. The influence of polarization functions on molecular orbital hydrogenation energies. *Theoret. Chim. Acta.*, *1972*, 213–222. DOI: 10.1007/BF00533485.

(8) J. P. Perdew; K. Burke; M. Ernzerhof. Generalized Gradient Approximation Made Simple. *Phys. Rev. Lett.* **1996**, *77*, 3865-3868. DOI: 10.1103/PhysRevLett.77.3865.

(9) Adamo, C.; Barone, V. Toward reliable density functional methods without adjustable parameters: The PBE0 model. *J. Chem. Phys.* **1999**, *110* (13), 6158–6170. DOI: 10.1063/1.478522.

(10) Zhao, Y.; Truhlar, D. G. The M06 suite of density functionals for main group thermochemistry, thermochemical kinetics, noncovalent interactions, excited states, and transition elements: two new functionals and systematic testing of four M06-class functionals and 12 other functionals. *Theoret. Chim. Acta* **2008**, *120* (1-3), 215–241. DOI: 10.1007/s00214-007-0310-x.

(11) O'Boyle, N. M.; Tenderholt, A. L.; Langner, K. M. cclib: A library for package-independent computational chemistry algorithms. *J. Comput. Chem.* **2008**, *29* (5), 839–845. DOI: 10.1002/jcc.20823.

(12) M. D. Hanwell; D. E. Curtis; D. C. Lonie; T. Vandermeersch; E. Zurek; G. R. Hutchison. Avogadro: an advanced semantic chemical editor, visualization, and analysis platform. *J. Cheminf.* **2012** (4), 1–17. DOI: 10.1186/1758-2946-4-17.

(13) O. Tange. Gnu parallel-the command-line power tool. *USENIX Magazine* **2011**, *36* (36), 42–47.

(14) Humphrey, W.; Dalke, A.; Schulten, K. VMD: visual molecular dynamics. *J. Mol. Graphics* **1996**, *14* (1), 33-38. DOI: 10.1016/0263-7855(96)00018-5.

(15) Dolomanov, O. V.; Bourhis, L. J.; Gildea, R. J.; Howard, J. A. K.; Puschmann, H. OLEX2 : a complete structure solution, refinement and analysis program. *J. Appl. Cryst.* **2009**, *42* (2), 339–341. DOI: 10.1107/S0021889808042726.

(16) Sheldrick, G. M. Crystal structure refinement with SHELXL. *Acta Cryst. C* **2015**, *71* (1), 3–8. DOI: 10.1107/S2053229614024218.

(17) Farrugia, L. J. WinGX and ORTEP for Windows : an update. *J. Appl. Cryst.* **2012**, *45* (4), 849–854. DOI: 10.1107/S0021889812029111.

(18) Spek, A. L. Single-crystal structure validation with the program PLATON. *J. Appl. Cryst.* **2003**, *36* (1), 7–13. DOI: 10.1107/S0021889802022112.

(19) Macrae, C. F.; Sovago, I.; Cottrell, S. J.; Galek, P. T. A.; McCabe, P.; Pidcock, E.; Platings, M.; Shields, G. P.; Stevens, J. S.; Towler, M.; Wood, P. A. Mercury 4.0: from visualization to analysis, design and prediction. *J. Appl. Cryst.* **2020**, *53* (1), 226–235. DOI: 10.1107/S1600576719014092.
